# Stakeholder Consensus on an Interdisciplinary Terminology to Enable the Development and Uptake of Medication Adherence Technologies Across Health Systems: Web-Based Real-Time Delphi Study

**DOI:** 10.2196/59738

**Published:** 2025-03-25

**Authors:** Alexandra Lelia Dima, Urska Nabergoj Makovec, Janette Ribaut, Frederik Haupenthal, Pilar Barnestein-Fonseca, Catherine Goetzinger, Sean Grant, Cristina Jácome, Dins Smits, Ivana Tadic, Job van Boven, Ioanna Tsiligianni, Maria Teresa Herdeiro, Fátima Roque, European Network to Advance Best Practices and Technology on Medication Adherence (ENABLE)

**Affiliations:** 1 Avedis Donabedian Research Institute (FAD) Universitat Autònoma de Barcelona (UAB) Barcelona Spain; 2 Avaluació de tecnologies sanitàries en atenció primària i salut mental (PRISMA) Institut de Recerca Sant Joan de Déu Esplugues de Llobregat Spain; 3 Centro de Investigación Biomédica en Red de Epidemiología y Salud Pública CIBERESP Madrid Spain; 4 Department of Social Pharmacy Faculty of Pharmacy University of Ljubljana Ljubljana Slovenia; 5 Institute of Nursing Science, Department Public Health Faculty of Medicine University of Basel Basel Switzerland; 6 Department of Theragnostic Hematology University Hospital of Basel Basel Switzerland; 7 Division of Nephrology and Dialysis Department of Medicine III Medical University of Vienna Vienna Austria; 8 CUDECA Institute for Training and Research in Palliative Care CUDECA Hospice Foundation Málaga Spain; 9 Observatoire national de la santé Strassen Luxembourg; 10 Prevention Science Institute University of Oregon Eugene, OR United States; 11 CINTESIS@RISE, MEDCIDS Faculty of Medicine University of Porto Porto Portugal; 12 Faculty of Health and Sports Sciences Institute of Public Health Riga Stradins University Riga Latvia; 13 Clinical Pharmacy Innsbruck University Innsbruck Austria; 14 Department of Clinical Pharmacy and Pharmacology, Medication Adherence Expertise Center of the Northern Netherlands (MAECON) University Medical Center Groningen University of Groningen Groningen The Netherlands; 15 Department of Social Medicine School of Medicine University of Crete Crete Greece; 16 Institute of Biomedicine Medical Sciences Department University of Aveiro Aveiro Portugal; 17 Research Unit for Inland Development Polytechnic of Guarda Guarda Portugal; 18 University Medical Center Groningen University of Groningen Groningen The Netherlands; 19 See Acknowledgements

**Keywords:** health technology, medication adherence, Delphi study, stakeholder engagement, digital health, behavioral science, implementation science

## Abstract

**Background:**

Technology-mediated medication adherence interventions have proven useful, yet implementation in clinical practice is low. The European Network to Advance Best Practices and Technology on Medication Adherence (ENABLE) European Cooperation in Science and Technology Action (CA19132) online repository of medication adherence technologies (MATechs) aims to provide an open access, searchable knowledge management platform to facilitate innovation and support medication adherence management across health systems. To provide a solid foundation for optimal use and collaboration, the repository requires a shared interdisciplinary terminology.

**Objective:**

We consulted stakeholders on their views and level of agreement with the terminology proposed to inform the ENABLE repository structure.

**Methods:**

A real-time web-based Delphi study was conducted with stakeholders from 39 countries active in research, clinical practice, patient representation, policy making, and technology development. Participants rated terms and definitions of MATech and of 21 attribute clusters on product and provider information, medication adherence descriptors, and evaluation and implementation. Relevance, clarity, and completeness criteria were rated on 9-point scales, and free-text comments were provided interactively. Participants could reconsider their ratings based on real-time aggregated feedback and revisit the survey throughout the study period. We quantified agreement and process indicators for the complete sample and per stakeholder group and performed content analysis on comments. Consensus was considered reached for ratings with a disagreement index of <1. Median ratings guided decisions on whether attributes were considered mandatory, optional, or not relevant. We used the results to improve the terminology and repository structure.

**Results:**

Of 250 stakeholders invited, 117 (46.8%) rated the MATech definition, of whom 83 (70.9%) rated all attributes. Consensus was reached for all items. The definition was considered appropriate and clear (median ratings 7.02, IPR 6.10-7.69, and 7.26, IPR 6.73-7.90, respectively). Most attributes were considered relevant, mandatory, and sufficiently clear to remain unchanged except for *ISO certification* (considered optional; median relevance rating 6.34, IPR 5.50-7.24) and *medication adherence phase*, *medication adherence measurement*, and *medication adherence intervention* (candidates for optional changes; median clarity ratings 6.07, IPR 4.86-7.17; 6.37, IPR 4.80-6.67; and 5.67, IPR 4.66-6.61, respectively). Subgroup analyses found several attribute clusters considered moderately clear by some stakeholder groups. Results were consistent across stakeholder groups and time, yet response variation was found within some stakeholder groups for selected clusters, suggesting targets for further discussion. Comments highlighted issues for further debate and provided suggestions informing modifications to improve comprehensiveness, relevance, and clarity.

**Conclusions:**

By reaching agreement on a comprehensive MATech terminology developed following state-of-the-art methodology, this study represents a key step in the ENABLE initiative to develop an information architecture capable of structuring and facilitating the development and implementation of MATech across Europe. The debates and challenges highlighted in stakeholders’ comments outline a potential road map for further development of the terminology and the ENABLE repository.

**International Registered Report Identifier (IRRID):**

RR2-10.1136/bmjopen-2021-059674

## Introduction

### Background

Technology-supported interventions for enhancing medication adherence have shown promise [1-3]. Addressing the prevalent low adherence to medication is likely to improve the use of limited resources and population health [4]. However, the sustainable implementation of health technologies in routine practice is lagging, in part due to the lack of accessibility to users in real-life settings [5]. Many repositories have been developed to improve accessibility and, thus, facilitate uptake of digital health [6], including digital apps [7] and medication adherence interventions [8]. Beyond accessibility, despite positive expectations from different stakeholders, digital health uptake is slowed down by misalignment of priorities and lack of a shared understanding of successful implementation, requiring dedicated efforts to increase communication and coordinated action from early development to routine use [9]. For medication adherence technologies (MATechs), additional barriers are the lack of awareness among stakeholders of the high prevalence and negative impact of suboptimal adherence and the lack of coordinated action for large-scale adoption of adherence-enhancing solutions across health systems [10].

The European Network to Advance Best Practices and Technology on Medication Adherence (ENABLE) European Cooperation in Science and Technology Action (CA19132) [11] was a 4-year project (2020-2024) funded by the European Commission bringing together >250 members from 40 countries to (1) raise awareness of medication adherence and related solutions, (2) expand multidisciplinary knowledge on medication adherence at multiple levels, (3) accelerate knowledge translation to clinical settings, and (4) encourage collaboration toward the implementation of MATech across health systems. Within ENABLE, a work package has been dedicated to identifying and showcasing MATech developed or available in participating countries by developing and maintaining a web-based repository where different stakeholders would be able to search for technologies that fit their needs, preferences, and context. The ENABLE repository aims to improve the accessibility of technologies though proactive and continued stakeholder involvement in building a community of practice that would sustain collaborative efforts throughout the process of technology development and implementation.

Managing the knowledge necessary for developing and using appropriate MATech for each health care context is challenging due to the complexity and heterogeneity of the relevant information and of its potential use contexts. This challenge is well known in medical informatics, which has a long history of developing standardized terminologies, classifications, and ontologies for knowledge management in health care [12]. These commonly agreed upon collections of “entities” (objects and phenomena in the world of health care) and their “attributes” (or properties) are used to encode specific types of information and, thus, structure knowledge in ways that allow for automated inference and learning. Such fundaments of information architecture are essential not only for health care delivery but also as a common language for multi-stakeholder communication about health care planning or improvement. Known examples are the World Health Organization (WHO) International Classification of Diseases [13]; International Classification of Functioning, Disability, and Health [14]; and International Classification of Health Interventions [15], as well as the Systematized Nomenclature of Medicine [16]. While well-established terminologies are intended to be comprehensive and evolve with the evolution of health sciences and clinical practice, newer terminologies continue to be proposed focusing on specific knowledge areas and aiming for interoperability with established terms. The recently developed Classification of Digital Health Interventions [17] and the Behaviour Change Intervention Ontology [18] are 2 examples relevant for MATech. The ENABLE work continues these efforts and has already established a definition of MATech and 385 attributes that represent the foundation of the ENABLE repository and MATech-related activities [19] within the broader context of an ENABLE terminology for intervention, technology, and practice established across working groups [20].

### Objectives

This paper reports on stakeholders’ views on the proposed MATech definition and attributes on which the ENABLE repository is built. We aimed to examine (1) the level of agreement on the relevance, clarity, and completeness of the proposed definition of MATech; (2) the level of agreement on the relevance, clarity, and completeness of the proposed framework of attributes; and (3) the stakeholders’ needs and requirements regarding MATech description.

## Methods

### Study Design and Process

An international interdisciplinary steering committee (SC) was formed by 11 ENABLE members to guide the development of the terminology and repository. The SC prepared the study protocol, proposed relevant attributes for MATech characterization, provided the rationale and methodology for the stakeholder consultation, managed the consultation process, and performed the data analysis and interpretation. The real-time Delphi stakeholder consultation was conducted on the eDelphi platform (Metodix Ltd), which allowed for anonymous data collection and management in line with European regulations on data protection. The real-time Delphi method is appropriate for capturing the views of large groups of experts and encouraging debate to work toward consensus on specific topics. The proposed terminology and the study protocol were improved based on feedback requested from 30 ENABLE members on face validity, user experience, and clarity of the instructions. The description of the ENABLE terminology and its development, the real-time Delphi survey design, and supporting materials are available in the published study protocol [19].

### Ethical Considerations

Ethics approval for the ENABLE Action, including this study, was granted by the Málaga Regional Research Ethics Committee (Comite de Etica de la Investigacion Provincial de Malaga) (CEI29.04.2021.MS1) on April 29, 2021, and a data protection assessment was performed by the Data Protection Officer at the University of Basel, which assessed the protocol as compliant with data protection and security standards. Participants were provided with study information and gave informed consent electronically on the study entry page. Anonymity was ensured via the eDelphi platform. No compensation was offered for participation. More details are reported in the published study protocol [19].

### Sampling and Sample Size

Purposive sampling was performed to identify at least 195 panelists from 39 countries participating in ENABLE at the time, aiming to represent 5 stakeholder groups in each country: adherence and eHealth research, clinical care, patient representation, policy and decision-making (PDM), and technology development. The sampling aimed to ensure variation in expertise, countries, and other characteristics such as years of expertise and gender. ENABLE Management Committee members in each country were requested to recommend at least 5 panelists among persons in their network whom they considered to have experience in medication adherence in at least one of the stakeholder groups mentioned previously; all recommended persons were invited to take part.

### Survey and Supporting Materials

The repository proposed to focus on MATech, defined as “devices, procedures or systems developed based on evidence to support patients to take their medications as agreed with health care providers (i.e., to initiate, implement and persist with the medication regimen)” following established definitions of related terms [21-24]. A framework of attributes was developed to describe MATech, covering 3 domains: *product and provider information* (domain 1), *medication adherence descriptors* (domain 2), and *evaluation and implementation* (domain 3). The survey consisted of several sections. First, information on the aim of the consultation, rules of engagement, and instructions for the survey and informed consent were provided. Second, participants were required to provide information on their sociodemographic characteristics and professional background, including gender, age, country, level of education, field of professional expertise (eg, medicine, pharmacy, nursing, psychology, or data science), stakeholder group (research, practice, PDM, patient representation, or technology development), and years of experience related to the field of medication adherence. Third, participants were asked to rate their level of agreement with and perceived clarity of the MATech definition and provide comments or revision suggestions as free text. Fourth, they were asked to familiarize themselves with the proposed terminology and provide general feedback on its completeness as free text. Next, attributes were presented to participants to rate their level of agreement with and clarity of each cluster and provide comments and suggestions. To facilitate response, attributes were grouped into 21 thematic clusters following the structure of the attribute framework. The questions addressing relevance and clarity were displayed in a Live2D format, a 2D grid where participants could rate relevance (on the x-axis) and clarity (on the y-axis) simultaneously on 9-point Likert scales, where 1 represented extremely irrelevant/unclear and 9 represented extremely relevant/clear. Participants provided their rating by placing a dot on the grid, which triggered the display in real time of all ratings of previous participants. They were encouraged to reconsider their ratings based on this information and rerate if needed. Participants were also able to view each other’s comments and were encouraged to interact in the free-text comment area for each cluster.

### Data Collection

Participants’ email addresses were entered into the survey management system, which generated automatic email invitations with personalized links. Before starting the survey, every participant provided informed consent electronically. Participants were able to access and answer the survey at any time during the study period between October 25, 2021, and January 15, 2022. To reach the full potential of the real-time approach, participants were encouraged to revisit the survey several times to follow and engage in discussions and revisit their ratings if their opinion changed. Participation was moderated by the study team to encourage interaction and discussions. Summaries of the study progress and active debates and encouragements to participate were provided weekly via email through the platform (Multimedia Appendix 1). The comment areas were monitored for any technical questions, which were answered in the same interactive area accessible to all, and the answers were included in the weekly summaries if appropriate. To monitor and analyze the evolution of survey responses in time, data were downloaded daily from day 7 to the end of the study period (78 days). This was necessary to bypass the platform limitation of overwriting previous data of individual participants if they decided to reconsider their answers. Data collection was stopped when three predetermined criteria were met: (1) a total response rate of ≥30%, (2) ≥10 complete responses in each stakeholder group, and (3) representation of two-thirds of the ENABLE countries. A survey response was considered complete if background questions and at least 75% of the questions on the terminology were answered.

### Data Analysis

Quantitative analyses were conducted in R (R Foundation for Statistical Computing) [25], and qualitative analyses were conducted in NVivo Pro (version 12; QSR International) [26]. Descriptive statistics were used for the background characteristics of the sample and to summarize responses on the terminology.

#### Level of Agreement on Relevance and Clarity

The level of agreement with the definition, completeness, and relevance and clarity of every attribute cluster was quantified using the interpercentile range adjusted for symmetry (IPRAS) analysis technique from the RAND/UCLA Appropriateness Method [27]. The disagreement index (DI) was calculated as a ratio between the interpercentile range (IPR) from the 30th to the 70th percentile and the IPRAS, and DI>1 (ie, IPR>IPRAS) was considered to indicate disagreement [27]. Levels of agreement were determined based on median ratings rounded to the nearest integer, and the DI was used to steer decisions about each cluster (Table 1).

**Table 1:** Criteria for levels of agreement and decisions about the attribute clusters.

Criterion	Relevance	Clarity
Median 7-9 and DI^a^<1	Attributes are relevant and mandatory.	Attributes are sufficiently clear to remain unchanged.
Median 4-6 or DI>1	Attributes are optional.	Proposed changes to the attributes are optional.
Median 1-3 and DI<1	Attributes are not relevant and candidates for exclusion.	Attributes are not clear and candidates for rephrasing.

^a^DI: disagreement index.

#### Subgroup Analyses

The descriptive analyses were repeated per stakeholder group to examine variation in opinions among stakeholders applying the criteria described previously and considering each participant in all the groups they identified themselves with. The reliability of the ratings per question within the stakeholder group was determined via intraclass correlation coefficients (ICCs) based on a 2-way random model considering type (average measures) and definition of relationship (consistency), with ICC>0.70 indicating moderate to good reliability [19]. To examine differences per item, 2-level models were performed for each item considering participants nested within stakeholder group. For these models, participants identifying with more than one group were allocated to the group least represented to maximize the equal distribution of participants in the groups.

#### Analysis of Process Indicators

The coefficient of quartile variation (CQV) was used to describe response stability (ie, the consistency of stakeholders’ responses) and was calculated at the end of the study for all participants (CQV_total_) and within the same stakeholder group (CQV_sub_), with CQV_total_<30% and CQV_sub_<15% indicating a stable (consistent) response [28]. Longitudinal changes in ratings for each question from individual participants, color coded by stakeholder group, and the evolution of median values of ratings for each stakeholder group were explored visually to gain deeper insights into the evolution of responses during the survey period.

#### Qualitative Analysis of Participants’ Interactive Feedback

Comments provided in free text were analyzed qualitatively using deductive content analysis based on the structure of the attribute framework [19]. Coding was performed independently by 2 researchers (UNM [pharmacy background and experienced in qualitative analysis] and FH [medicine background and trained new user of qualitative methods]). Both coders had in-depth knowledge of the terminology and had lead roles in managing the consultation process. Discrepancies were discussed and harmonized through an iterative process, initially between the 2 coders and then in discussions with other SC members (AD, JR, TH, and FR). This process generated a revised thematic tree, which was presented in a narrative and table format. Proposed modifications to the terminology were considered if suggested by median ratings or DIs and reflected in participants’ free-text comments. The qualitative results guided improvements in the attribute framework (eg, rewording labels and definitions and excluding or adding attributes) and the subsequent development of the ENABLE repository.

## Results

### Participation Rates and Sample Characteristics

From 250 invited panelists, 174 (69.6%) started the survey, 117 (46.8%) panelists from 29 countries answered the questions on background information and the definition of MATech, and 83 (33.2%) panelists from 27 countries completed the survey (see details in the data analysis report in Multimedia Appendix 2). On average, 3 (SD 2) panelists represented each of the 29 countries, with a minimum of 1 per country (2 countries) and a maximum of 10 per country (1 country). The countries represented in the real-time Delphi study were Albania, Belgium, Bosnia and Herzegovina, Bulgaria, Croatia, Cyprus, Czech Republic, Denmark, Finland, France, Germany, Greece, Hungary, Italy, Latvia, Malta, the Netherlands, Norway, Poland, Portugal, Romania, Serbia, Slovenia, Spain, Sweden, Switzerland, and the United Kingdom. The panel consisted predominantly of women (74/117, 63.2%), participants with PhD-level education (64/117, 54.7%), health care professionals (HCPs; 85/117, 72.6%), and participants with background in research or health care practice (74/117, 63.2% and 62/117, 53%, respectively); 55.6% (65/117) of the participants identified with 1 background, whereas others identified with up to 5 backgrounds (13/117, 11.1%; Table 2).

**Table 2:** Characteristics of panelists who completed medication adherence technology (MATech) definition ratings and panelists who completed the survey.

		Completed MATech definition rating (n=117), n (%)	Completed the survey (n=83), n (%)
**Sex**			
	Female	74 (63.2)	54 (65.1)
	Male	43 (36.8)	29 (34.9)
**Age (years)**			
	18-30	4 (3.4)	2 (2.4)
	31-40	28 (23.9)	22 (26.5)
	41-50	43 (36.8)	28 (33.7)
	51-60	35 (29.9)	26 (31.3)
	61-70	7 (6)	5 (6)
**Educational level**			
	Bachelor’s degree	4 (3.4)	3 (3.6)
	Doctorate (PhD)	64 (54.7)	42 (50.6)
	High school diploma	4 (3.4)	3 (3.6)
	Master’s degree	28 (23.9)	23 (27.7)
	Specialty degree (health care)	17 (14.5)	12 (14.5)
**Expertise**			
	Computer science or software engineering	5 (4.3)	4 (4.8)
	Data science or statistics	5 (4.3)	4 (4.8)
	Economics or management	11 (9.4)	8 (9.6)
	Medicine	26 (22.2)	16 (19.3)
	Nursing	11 (9.4)	11 (13.3)
	Pharmacy	48 (41)	33 (39.8)
	Psychology	3 (2.6)	2 (2.4)
	Sociology	1 (0.9)	0 (0)
	Other	7 (6)	5 (6)
**Background^a^**			
	Research or education	74 (63.2)	50 (60.2)
	HCP^b^	62 (53)	45 (54.2)
	PDM^c^	29 (24.8)	19 (22.9)
	Patient representative	26 (22.2)	18 (21.7)
	eHealth developer	32 (27.4)	24 (28.9)
**Number of backgrounds**			
	1	65 (55.6)	49 (59)
	2	27 (23.1)	16 (19.3)
	3	9 (7.8)	7 (8.4)
	4	3 (2.6)	1 (1.2)
	5	13 (11.1)	10 (12)

^a^Multiple answers possible for this question.

^b^HCP: health care professional.

^c^PDM: policy and decision-making.

### Level of Agreement on Relevance and Clarity

**Figure 1:**
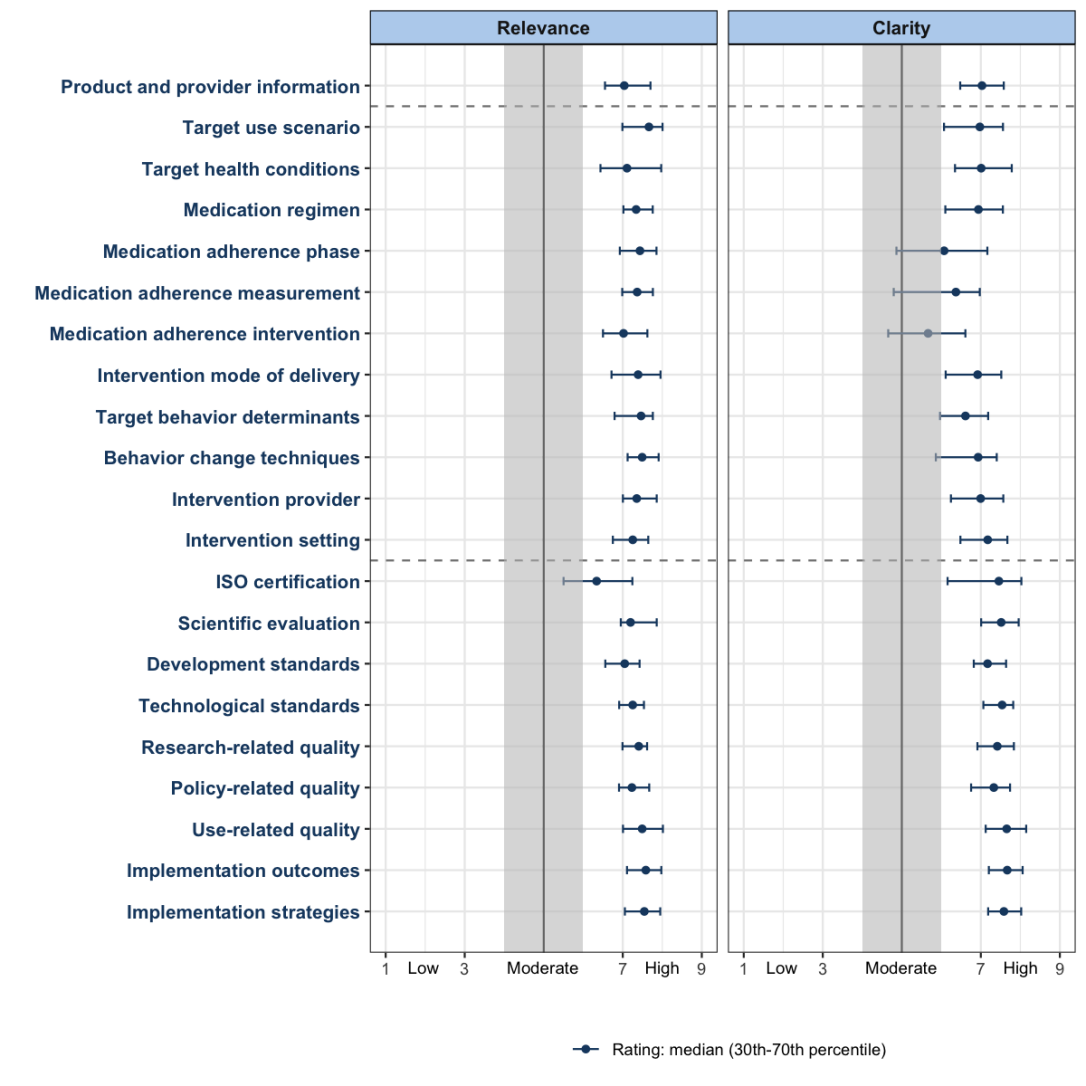
Median ratings with interpercentile range for relevance and clarity per attribute cluster (N=83). ISO: International Organization for Standardization.

No disagreements we identified among the 46.8% (117/250) of the participants who contributed to definition and attribute rating. The median level of agreement with the proposed MATech definition was 7.02 (IPR 6.10-7.69), and the median clarity rating was 7.26 (IPR 6.73-7.90). Thus, the definition was considered relevant and sufficiently clear. The median ratings of relevance and clarity for attribute clusters based on 33.2% (83/250) of the participants are presented visually in Figure 1. A total of 95% (20/21) of attribute clusters were considered relevant (Table S1 in Multimedia Appendix 3), whereas 1 attribute (*ISO standard*) received a moderately relevant rating (median 6.34, IPR 5.50-7.24). *Target use scenario* received the highest rating (median 7.66, IPR 6.99-8.01). Thus, most attributes were considered relevant and mandatory for inclusion in the repository except for *ISO standard*, which was considered optional. In total, 86% (18/21) of the attribute clusters were considered clear (Table S2 in Multimedia Appendix 3). A total of 14% (3/21) of the attribute clusters were rated as moderately clear: *medication adherence phase* (median 6.07, IPR 4.86-7.17), *medication adherence measurement* (median 6.37, IPR 4.80-6.67), and *medication adherence intervention* (median 5.67, IPR 4.66-6.61). *Implementation outcomes* received the highest clarity rating (median 7.67, IPR 7.20-8.06). Thus, most attributes were considered sufficiently clear to remain unchanged, whereas for 3 attributes, changes could be considered if appropriate to improve clarity.

### Subgroup Analyses

No disagreements were identified at the subgroup level among researchers, HCPs, PDM stakeholders, patient representatives, and technology developers. For the MATech definition, levels of agreement and clarity ratings within the subgroups were above the established thresholds for sufficient relevance and clarity (Table 3). For attribute clusters (Figure S1 in Multimedia Appendix 3), relevance ratings within all subgroups were above the established thresholds for being considered relevant and sufficiently clear except for *ISO certification* (considered optional by all subgroups) and *intervention setting* (considered optional only by technology developers). Clarity ratings within subgroups confirmed the full sample results for *medication adherence phase* and *medication adherence intervention*, which were perceived as moderately clear by all subgroups. *Medication adherence measurement* ratings were above the clarity threshold for subgroups, suggesting that participants identifying with multiple subgroups rated this attribute higher on clarity. Moreover, *target behavior determinants* was considered moderately clear by patient representatives, and *intervention provider* was considered moderately clear by PDM stakeholders, patient representatives, and technology developers.

Across attribute clusters, the ICC value for the median ratings of the 5 subgroups was 0.95 for relevance and 0.97 for clarity, indicating good interrater reliability between subgroups (considered as “raters”). To describe the differences specific to each attribute cluster, 41% (34/83) of the participants, who identified with more than one group, were allocated to the least represented group. This decision resulted in 33% (27/83) of the participants in the research group, 22% (18/83) in the HCP group, 10% (8/83) in the PDM group, 22% (18/83) in the patient representative group, and 14% (12/83) in the technology developer group. For most attribute clusters, the proportion of variance explained by the stakeholder group was either 0 (model almost or near singular) or <0.1, with only 2 items with an ICC of >0.15 (ie, *target health conditions* [relevance; ICC=0.19] and *intervention setting* [relevance; ICC=0.205]). Thus, ≥80% of the variance was within groups, and therefore, the differences across groups did not justify further investigation per item (Multimedia Appendix 4).

**Table 3:** Descriptive statistics for level of agreement with and clarity of medication adherence technology definition per stakeholder subgroup.

Subgroup and criterion		Median	30th-70th percentile	Interpercentile range	Interpercentile range adjusted for symmetry	Disagreement index
**Researchers (n=50)**						
	Agreement	7.05	6.10-7.77	1.67	5.25	0.32
	Clarity	7.48	6.91-8.01	1.10	6.04	0.18
**Health care professionals (n=45)**						
	Agreement	7.01	6.11-7.45	1.34	5.02	0.27
	Clarity	7.38	6.51-7.86	1.35	5.63	0.24
**Policy and decision-making(n=19)**						
	Agreement	6.86	6.19-7.40	1.22	5.05	0.24
	Clarity	7.55	7.14-7.97	0.84	6.19	0.14
**Patient representatives (n=18)**						
	Agreement	6.74	5.99-7.20	1.20	4.74	0.25
	Clarity	7.09	6.63-7.74	1.12	5.63	0.20
**Technology developers (n=24)**						
	Agreement	6.74	6.01-7.41	1.40	4.92	0.28
	Clarity	7.60	7.01-8.01	1.00	6.11	0.16

### Process Indicators

For the whole sample, CQV values for the MATech definition were 13.23 for agreement and 12.74 for clarity, indicating consistency (<30%); consistent responses were found for all attribute clusters. Subgroup analyses found CQV values below the established threshold for most subgroups (<15%), with some exceptions. For the MATech definition, response variation was found among HCPs and technology developers. For relevance (Table 4), response variation was found among technology developers regarding *medication adherence intervention* and among researchers, HCPs, PDM stakeholders, and technology developers regarding the *ISO certification.* For clarity (Table 5), response variation was found among one or more stakeholder groups regarding 48% (10/21) of the attribute clusters. Notably, *target health conditions* was below the stability threshold for patient representatives and technology developers; *medication adherence phase* was below the stability threshold for researchers, HCPs, patient representatives, and technology developers; *medication adherence measurement* was below the stability threshold for researchers, PDM stakeholders, and patient representatives; *medication adherence intervention* was below the stability threshold for researchers and patient representatives; *target behavior determinants* was below the stability threshold for technology developers; *intervention setting* was below the stability threshold for researchers, HCPs, patient representatives, and technology developers; and *ISO certification* was below the stability threshold for researchers and patient representatives. Moreover, *target use scenario*, *medication regimen*, *intervention mode of delivery*, and *behavior change techniques* were below the stability threshold for patient representatives only.

**Figure 2:**
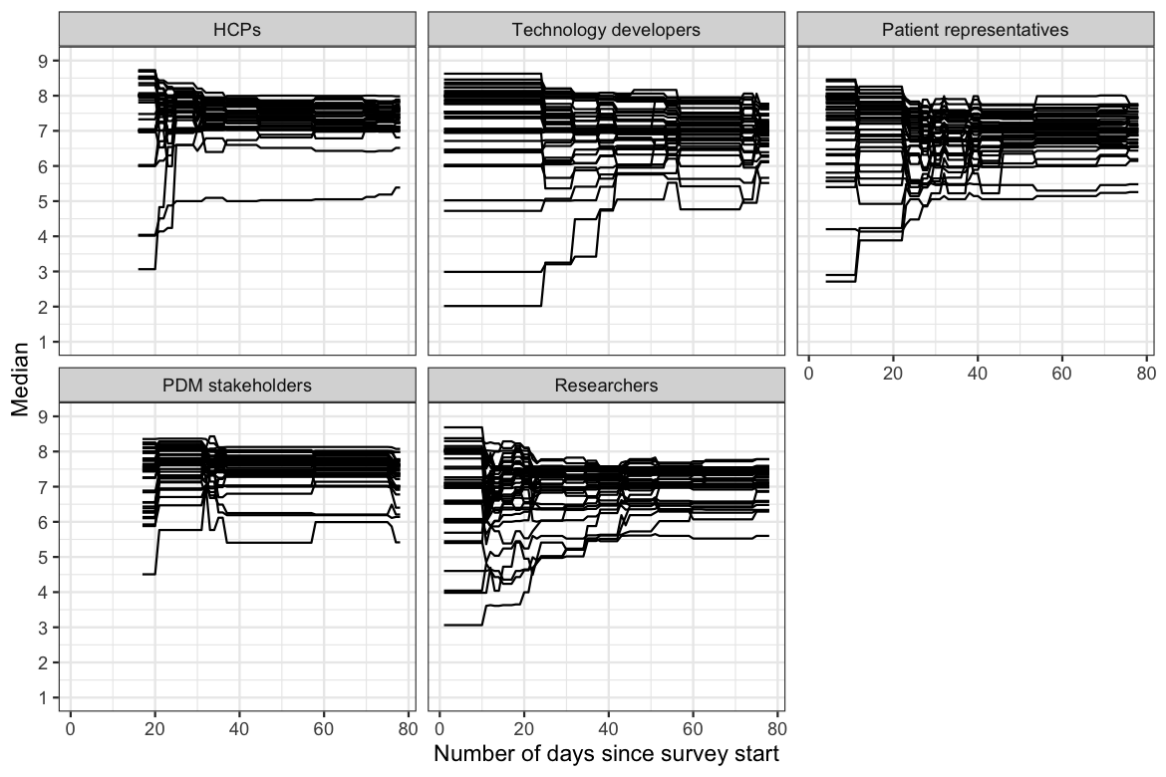
Evolution of median ratings of relevance and clarity per stakeholder group. Each line represents the median value of the ratings for 1 attribute cluster and property (relevance or clarity) calculated on each day of the survey based on the responses of the participants to date. HCP: health care professional; PDM: policy and decision-making.

Most participants did not change their ratings throughout the real-time Delphi survey (Multimedia Appendix 4). The visualization of the evolution of median ratings for relevance and clarity for each attribute cluster per stakeholder group (Figure 2) shows a stabilization of the median values in the second half of the real-time Delphi period at levels above the threshold values.

**Table 4:** Coefficient of quartile variation (CQV) overall and per subgroup for relevance (N=83). Values below the relevance threshold are italicized.

Rating item	CQV total	CQV for researchers	CQV for health care professionals	CQV for policy and decision-making	CQV for patient representatives	CQV for technology developers
Medication adherence technology definition (agreement)	13.23	14.15	16.54	6.05	12.05	17.62
Product and provider information	11.14	10.09	6.99	6.54	13.75	9.77
Target use scenario	7.88	8.69	6.66	6.72	8.63	10.62
Target health conditions	11.35	12.62	8.42	4.29	18.47	13.03
Medication regimen	7.38	6.32	6.57	3.07	11.64	11.77
MA^a^ phase	8.47	8.07	6.68	5.44	10.64	8.16
MA measurement	6.73	4.98	4.61	10.37	6.41	8.28
MA intervention	11.23	9.56	5.01	9.56	9.19	*19.95* ^b^
Intervention mode of delivery	10.08	6.47	7.34	8.17	10.65	8.96
Target behavior determinants	7.08	6.33	2.88	8.05	7.06	3.42
Behavior change technique	6.71	6.04	6.72	5.29	10.05	11.32
Intervention provider	8.38	4.84	4.92	3.71	6.94	8.74
Intervention setting	10.55	8.97	7.42	7.73	8.84	7.88
ISO certification	18.84	*19.62*	*18.55*	*19.64*	11.64	*31.10*
Scientific evaluation	7.52	9.32	7.55	5.44	12.28	7.53
Development standards	10.99	8.89	6.67	3.03	11.23	9.74
Technological standards	6.17	6.37	4.06	3.61	5.24	6.47
Research-related quality	5.87	6.02	5.43	3.35	8.45	5.01
Policy-related quality	7.00	6.08	5.10	6.89	9.26	5.93
Use-related quality	7.61	7.25	6.86	4.44	9.19	9.40
Implementation outcomes	6.41	6.46	5.31	4.66	6.28	9.54
Implementation strategies	6.78	7.76	4.96	7.37	6.19	6.05

^a^MA: medication adherence.

^b^Values below the relevance threshold are italicized.

**Table 5:** Coefficient of quartile variation (CQV) overall and per subgroup for clarity (N=83). Values below the clarity threshold are italicized.

Rating item	CQV total	CQV for researchers	CQV for health care professionals	CQV for policy and decision-making	CQV for patient representatives	CQV for technology developers
Medication adherence technology definition (agreement)	12.74	12.01	13.76	6.58	10.43	14.50
Product and provider information	13.65	12.53	8.68	4.06	10.03	11.43
Target use scenario	13.1	13.18	13.83	8.69	*15.62* ^a^	10.61
Target health conditions	13.14	8.74	7.69	13.84	*16.37*	*21.60*
Medication regimen	12.77	12.75	9.02	7.95	*18.72*	14.91
MA^b^ phase	25.03	*21.49*	*23.47*	6.84	*28.13*	*37.23*
MA measurement	21.26	*20.51*	12.31	*18.74*	*29.43*	14.92
MA intervention	13.12	*19.95*	9.1	9.39	*27.3*	13.34
Intervention mode of delivery	12.61	7.9	11.27	11.65	*19.54*	14.21
Target behavior determinants	14.24	14.34	6.15	11.75	14.64	*20.96*
Behavior change technique	11.97	12.49	9.32	13.19	*19.29*	8.38
Intervention provider	10.75	8.42	7.23	6.57	14.55	14.64
Intervention setting	23.72	*22.19*	*20.55*	14.68	*18.61*	*26.34*
ISO certification	14.91	*18.71*	12.4	6.45	*17.57*	9.73
Scientific evaluation	7.41	6.64	7.6	12.2	12.4	14.3
Development standards	7.77	9.27	8.33	8.17	4.19	8.41
Technological standards	6.33	6.7	3.25	2.58	6.69	12.32
Research-related quality	10.02	8.59	4.35	5.02	14.35	7.54
Policy-related quality	8.28	8.64	4.17	8.59	9.42	8.4
Use-related quality	7.9	8.02	4.22	4.84	10.33	8.04
Implementation outcomes	6.97	6.54	7.66	3.87	7.45	5.35
Implementation strategies	6.87	7.28	6.2	6.57	3.83	7.3

^a^Values below the clarity threshold are italicized.

^b^MA: medication adherence.

### Qualitative Analysis of Participants’ Interactive Feedback

#### Overview

We examined the feedback provided in the interactive comment area of each survey page, pertaining to 6 predefined topics: general feedback on the survey and on the terminology, specific feedback on the MATech definition, and feedback on the 3 attribute domains (*product and provider information*, *medication adherence descriptors*, and *evaluation and implementation*). In addition to identifying suggestions for improving the terminology, we aimed to understand stakeholders’ needs and expectations regarding MATech description that would guide further steps in the development of the ENABLE repository and community of practice. Further details and representative quotes are presented in Multimedia Appendix 5.

#### General Feedback on the Terminology

Several participants noted the complexity and comprehensiveness of the terminology. Some highlighted challenges related to its use for the repository (eg, anticipating how the framework would need to be translated in a data collection tool and expressing concerns on the feasibility of data collection and long-term maintenance of the repository). Some comments pointed to the importance of representing patient perspectives in the terminology (eg, by including patient-related determinants of adherence and technology use such as medication burden, affordability, or inequities of access).

#### Feedback on the MATech Definition

Many participants commented on the MATech definition, with several aspects generating debate. Referring to the term “technology,” some participants considered that MATech should be understood as digital tools, whereas others considered that a broader scope (eg, including also analog tools and services) was appropriate. Different opinions were expressed on whether “medication adherence” stood for taking medicines as *prescribed by* or as *agreed with* an HCP. Debate ensued on the meaning of the term “evidence based,” especially concerning the strength of the evidence needed and finding a balance between inclusiveness and evidence standards (ie, between including technologies under development for which limited evidence is available vs limiting the repository to technologies that meet higher thresholds of evidence).

#### Feedback on Product and Provider Information

In this first domain, 2 general questions were raised. Some participants were concerned about how the product maturity (versioning) would be handled in the repository and how products that comprised a combination of technologies could be described. The implementation of these terms in the repository would need to consider these different scenarios.

Some comments referred to the need to consider the intended use of the product as a first question and adapt the description depending on the response to this question.

#### Feedback on Medication Adherence Descriptors

In this second domain, there was debate over the distinction between monitoring and support as 2 elements of adherence management. Some comments recommended to follow the Ascertaining Barriers to Compliance medication adherence taxonomy [21] without adaptations. Others questioned whether an MATech could only monitor the patient without also intervening and affecting their behavior. Inclusion of behavior change techniques following the capability, opportunity, motivation, behavior model was viewed positively by some participants, whereas others questioned whether these models are widely known and simple enough for their use in practice, for example, by HCPs. Suggestions were given on clarifying how certain types of MATech could be described according to the proposed attributes (eg, modes of delivery and provider and setting).

#### Feedback on Evaluation and Implementation

The third domain was the least commented on. The need for an evidence base, especially scientific evidence, was challenged and considered conditional on the intended use of the technology. Participants expressed that the implementation outcomes and strategies should be more visible in the terminology. Some overlap was noted in the proposed attributes. Several comments referred to the lesser relevance of the ISO certification.

#### Stakeholders’ Needs and Expectations

Some comments highlighted contrasting needs and expectations from participants regarding the MATech description. Some expected more detailed descriptions to provide comprehensive information for different potential users, whereas others preferred more succinct descriptions feasible for MATech providers to enter into the platform. Several comments expressed expectations for continued testing and adaptation of the terminology to evolving technologies. Ensuring accurate information, continuously evaluating MATech, and updating MATech descriptions regularly (eg, every year) were highlighted as important expected features for the repository to provide value to potential users in the context of a rapidly changing field.

## Discussion

### Principal Findings

**Figure 3:**
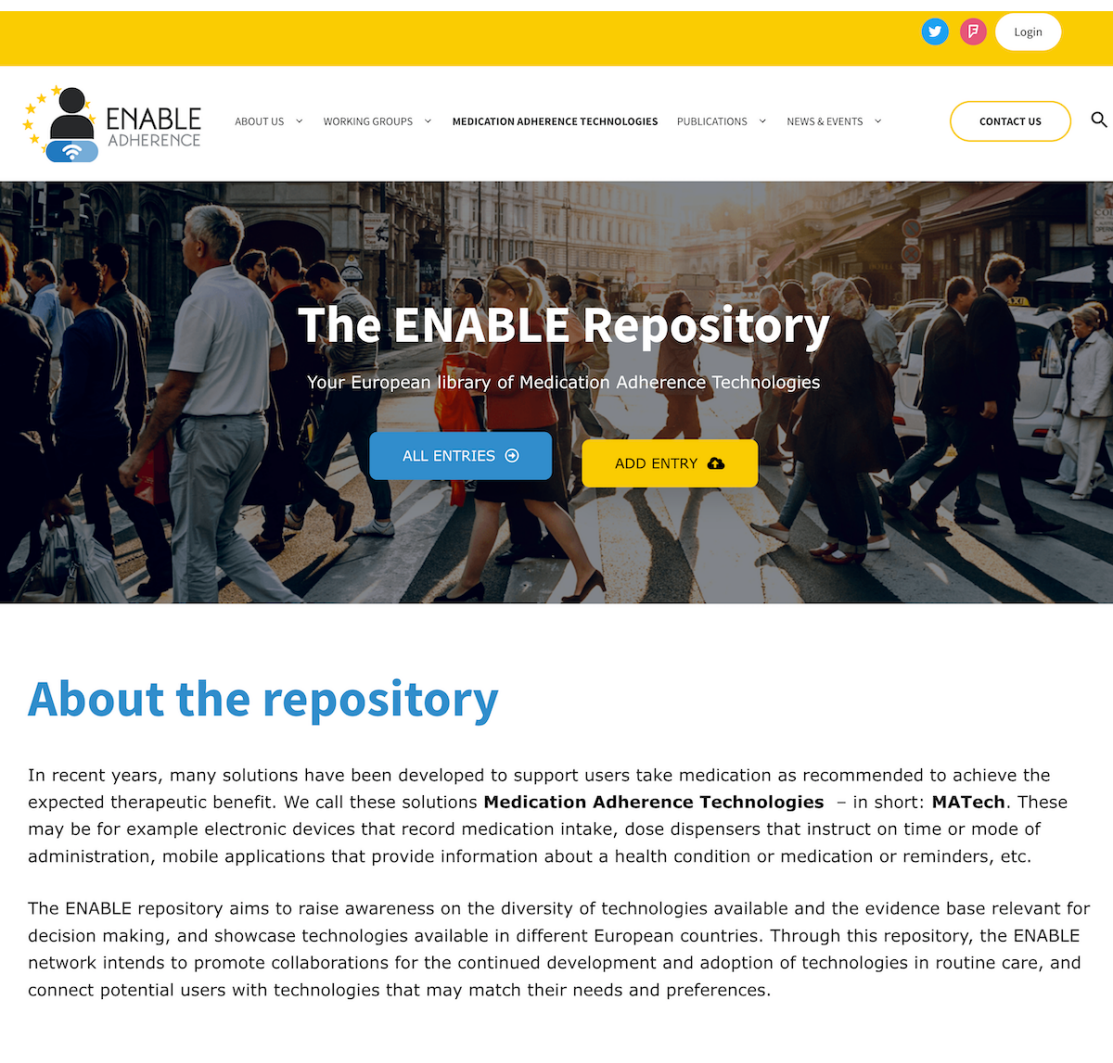
Screenshot of the European Network to Advance Best Practices and Technology on Medication Adherence (ENABLE) repository as available at manuscript submission date.

The ENABLE European Cooperation in Science and Technology Action offered the opportunity to develop a comprehensive interdisciplinary terminology regarding MATech aligned with existing classifications and ontologies and to consult stakeholders from diverse backgrounds and geographical locations on the terminology’s relevance and clarity. We found agreement among stakeholders on the MATech definition and attribute clusters, which indicates an interdisciplinary network ready for collaboration on this conceptual basis. Most terms were considered relevant and clear, and several moderate ratings and related comments suggested further improvements, which informed a new version of the terminology. This initial consensus represents a strong foundation for current efforts to advance the development and use of technologies in medication adherence management across health systems, in which the ENABLE network and collaborating organizations have already made important progress [10,11,20]. This conceptual foundation was an important step in the development of the ENABLE repository (Figure 3 [29]). However, the variation in opinions within stakeholder groups and debates that ensued in the interactive feedback areas over several topics point to the need to continue engaging stakeholders in constructive and inclusive discussions to align conceptual representations and expectations on how they can guide concrete action.

### Interpretation and Comparison to Prior Work

It was agreed that the MATech definition was appropriate and clear; however, several debates ensued regarding “technology,” “medication adherence,” and “evidence.” These debates have practical implications for the ENABLE repository and network. First, the term “health technology,” which we adopted from the WHO definition as “the application of organized knowledge and skills in the form of devices, medicines, vaccines, procedures and systems developed to solve a health problem and improve quality of lives” [23], was considered by some participants as too broad for the scope of the repository. At the same time, the attribute framework was criticized for not explicitly including types of technologies considered of high potential, such as bio- and nanotechnologies. Considering this feedback, it was agreed that the scope of the ENABLE repository would focus initially on digital technologies, with the possibility of further extension to other types of technologies in the future. Nevertheless, these different perspectives highlight the challenges inherent in structuring and describing health innovation. The discussion that ensued on the term “medication adherence” as taking medicines “as prescribed” or “as agreed” reflects a long-standing challenge of harmonizing descriptive versus prescriptive definitions of medication adherence. From a descriptive viewpoint, the process of medication taking starts once a prescription has been issued; is operationalized as the comparison between 2 time series (ie, of prescribed and actual dosing); and is distinct from its determinants, including the process of shared decision-making leading to an agreement on the treatment regimen between the patient and their health care team. These distinctions are essential for accurate measurement in research and evaluation [30]. From a prescriptive viewpoint, adhering to a medication regimen would require as a precondition an appropriately prescribed treatment resulting from a process of decision-making that involves mutual agreement on a course of treatment for which responsibility is shared between the patient and health care team [24]. Thus, integrating treatment agreement into the broader process of medical treatment is essential for a person-centered approach to medication taking. Harmonizing these perspectives requires further consensus work with the involvement of different stakeholders. Third, the role of scientific evidence was debated, with some participants underlining the necessity of including only MATech supported by scientific evidence in the repository and others promoting a more inclusive approach to MATech selection. Following this feedback, we adopted the technology readiness level (TRL) as a framework to guide eligibility, including MATech with at least a small-scale prototype validated in a local environment; however, challenges remained regarding mapping candidate MATech onto the TRL framework [31]. On the basis of these considerations and further work within the ENABLE network on a higher-level ENABLE terminology including medication adherence–enhancing interventions, reimbursement, and best practice, the MATech definition has evolved to “evidence-based health technologies used in the management of medication adherence by different stakeholders” [20]. Therefore, the initial version of the repository includes MATech that (1) are presented as aiming to address medication adherence management (by either obtaining information or offering support), (2) have a digital component as per the DigitalHealthEurope definition [32], and (3) meet at least TRL 4. These criteria are likely to evolve depending on the interest in and uses of the repository by different stakeholder groups.

Most attribute clusters were also considered relevant and clear except for *ISO qualification*, which was considered moderately relevant, and the *medication adherence phase*, *medication adherence measurement*, and *medication adherence intervention* attributes, which were considered moderately clear. The comments received guided improvements; for example, the *ISO qualification* attribute was changed to a broader and more flexible attribute of *certification label*, and the definitions of the medication adherence–related attributes were modified to improve clarity and refer to the Ascertaining Barriers to Compliance taxonomy [21], which informed their selection. The comments on each attribute domain were taken into account by modifying labels and definitions of some terms and restructuring others or in the formulation of the MATech data collection form. While these modifications were considered sufficient for the initial development of the repository, it is necessary to continue improving this terminology and allow it to evolve in synchrony with similar efforts in related fields. In particular, the methodology and evaluation criteria for health technology are evolving rapidly. For example, a recent Delphi study on the assessment of patient-facing eHealth tools reached consensus on 46 criteria classified as “foundational” (eg, technical aspects, clinical utility, and safety) and “contextual” (eg, data protection compliance, safety regulatory compliance, interoperability, and data integration) [33]. While this extensive assessment specific to different use scenarios can be applicable to MATech as well, a briefer description specific to medication adherence would be more appropriate for an initial overview of an MATech at a given moment, which could be followed up with more extensive examination. To facilitate the repository development and its function of boosting research collaborations within the ENABLE network, we subsequently chose to include only a selection of attributes in the MATech description and develop support materials for participating countries to search, contact, and involve MATech developers in describing their products on the ENABLE website. To date, 18 countries have started the MATech search process, and 8 have included at least one MATech in the repository. User feedback will continue to be gathered to inform the iterative development of the terminology and repository.

The subgroup analyses and process indicators showed limited variation in responses between and within groups and a stabilization of responses halfway through the survey. This stability and convergence toward above-threshold ratings for relevance and clarity indicate a strong basis for multi-stakeholder action on this topic. However, there were some attribute clusters that showed moderate clarity only for some stakeholder groups (ie, *target behavior determinants* for patient representatives and *intervention provider* for PDM stakeholders, patient representatives, and technology developers). While these ratings suggest only optional modifications, it is important to interpret these findings as a recommendation to reword and explain professional terminology in all stakeholder-facing materials. Participants recommended repeatedly adapting the technical language to diverse audiences, and this advice has been considered in the development of repository-related materials and needs to guide further developments.

Developing a consensus terminology to structure the description and facilitate the search of available solutions has been used in developing repositories in related areas, such as the taxonomy of self-management interventions on which the Comparing Effectiveness of Self-Management Interventions in 4 High-Priority Chronic Diseases in Europe (COMPAR-EU) platform was built [34]. These frameworks prove useful in systematic searches and descriptions of published research evidence and available digital products, as performed in the COMPAR-EU project [35] and in ENABLE [36]. Other repositories may not rely on an extensive conceptual development and rather adopt a limited number of descriptive terms to serve for initial explorations of the available offer in a specific topic or geographical area. This is the case the Radar Digital Health Uptake [6], the different country-specific repositories cataloged on the European mHealth Hub [7], and Interventienet for medication adherence interventions [8]. As the digital health field will continue to develop, it becomes increasingly relevant to work on the integration and continuity of these platforms. Ontological approaches may provide part of the solution for the technical integration, whereas consensus methodologies such as the Delphi method could be used to reach agreement on policy priorities and their implementation.

### Strengths and Limitations

This real-time Delphi study succeeded in engaging a diverse stakeholder panel regarding geographical location, professional experience, and sociodemographic characteristics and in reaching a consensus on a comprehensive terminology to inform research on and adoption of MATech. The real-time web-based format facilitated interactions and flexible participation of panelists at their chosen time and provided valuable feedback for further improvements. The subgroup and process analyses allowed for a more in-depth understanding of the variation in opinions and, thus, potential avenues for future stakeholder engagement. However, 2 main limitations are important to consider. First, some limitations of the tool did not permit a full analysis of the change in opinions and might have overestimated the level of consensus. For example, once a panelist provided an initial response to a question, they were able to see the responses of others and readjust their response in light of this information, in which case the initial response was overwritten. Nevertheless, survey data were downloaded every day to capture temporal changes. Therefore, while we could not capture changes between initial and reconsidered responses if such changes occurred within the same day, changes occurring in consecutive days were identified. Second, participation rates were <50%, and few participants engaged with the survey multiple times during the survey period. While we attempted to encourage participation via pretesting of materials and weekly summaries emailed to all participants, we could not overcome other likely barriers such as the complexity of the participation task and the novelty of the real-time web-based Delphi format. Thus, our findings likely represent a selected population, and future work on the ENABLE terminology would need to involve a wider audience and apply different methods to communicate and ask for feedback about medication adherence–related terms and their different practical applications.

### Future Directions

This initial work opens several avenues for potential development of the ENABLE terminology. An important next step is to develop it into an ontology of MATech research and deployment in health systems in line with methodological recommendations [37]. While our terminology closely followed ontology development principles and steps [19], further work remains to be done, such as testing interrater reliability, specifying relationships between entities, and disseminating and maintaining the future ontology. This work would need to be integrated into the sustainability of the ENABLE repository and community of practice. The ENABLE Action has been encouraging the development of national centers and networks of medication adherence in collaboration with stakeholder organizations [10]. These centers and the pan-European community that has developed during the ENABLE project could adopt and further improve this terminology and conduct translations into different languages following recent examples of other terminologies [38,39]. The repository is intended as a common platform for developing and implementing digital adherence technologies in Europe and needs to be supported by policies that acknowledge medication adherence as a priority for coordinated action, inform of available solutions, and incentivize and support their deployment [40].

Beyond its use to inform the repository, the ENABLE terminology can be applied to assess stakeholder needs and develop training, for example, training HCPs in selecting and using MATech in their practice or MATech developers in assessing user needs and health system requirements for technology adoption. Part of the attribute framework was already used in developing content for an interdisciplinary ENABLE training school in 2023, and other materials are under development. In the long term, the more consistent use of MATech-related terms and definitions has the potential to lead to better standardization of research and implementation efforts, as well as of recording practices in real-life settings, by providing the information architecture to collect real-world data for the development, evaluation, and implementation of MATech in line with current developments to use real-world data for agile regulatory approval [41]. As medication adherence is a transversal topic in health services worldwide, we can envisage the possibility of using future iterations of this taxonomy for standardized operationalization of medication adherence measurement and intervention into digital systems, for example, within the innovative Digital Adaptation Kits recently piloted by the WHO in several clinical areas [42]. Given its built-in interoperability, the ENABLE terminology can also be expanded to encompass terms describing other related medication use processes (eg, medication review and prescribing practices) and other self-management and health behaviors (eg, dietary recommendations, physical exercise, sedentariness, sleep, and alcohol and tobacco use). Continuing its development with an active engagement of patients and other stakeholders would allow for the setting of priorities in line with users’ expectations and, thus, maximizing its utility. Challenges remain on aligning stakeholders and maintaining a community of practice that would apply and evolve this taxonomy to serve the effective and efficient development and use of MATech in health systems.

### Conclusions

This paper reports reaching an agreement among diverse stakeholders from 27 countries on a comprehensive MATech terminology developed following state-of-the-art methodologies. Therefore, this study represents a key contribution toward the ambitious goal of building an information architecture to facilitate the development and implementation of MATech in health systems. Further improvements to this terminology, together with knowledge mobilization activities such as interdisciplinary networking and training across stakeholder groups, would ensure a shared conceptual and practical foundation for efficient innovation in the field of medication adherence.

## Appendix

Multimedia Appendix 1 Delphi survey bulletins.

Multimedia Appendix 2 Delphi survey data and analysis script.

Multimedia Appendix 3 Relevance and clarity ratings for the full sample and stakeholder subgroups.

Multimedia Appendix 4 Quantitative data report.

Multimedia Appendix 5 Qualitative data report.

## Abbreviations

COMPAR-EU: Comparing Effectiveness of Self-Management Interventions in 4 High-Priority Chronic Diseases in Europe
CQV: coefficient of quartile variation
DI: disagreement index
ENABLE: European Network to Advance Best Practices and Technology on Medication Adherence
HCP: health care professional
ICC: intraclass correlation coefficient
IPR: interpercentile range
IPRAS: interpercentile range adjusted for symmetry
MATech: medication adherence technology
PDM: policy and decision-making
SC: steering committee
TRL: technology readiness level
WHO: World Health Organization
