# Supplementary material for: Stakeholder Consensus on an Interdisciplinary Terminology to Enable the Development and Uptake of Medication Adherence Technologies Across Health Systems: Web-Based Real-Time Delphi Study
Source: J Med Internet Res. 2025 Mar 25;27:e59738. doi: 10.2196/59738 (PMC11979531; doi:10.2196/59738)

# ENABLE Delphi study bulletins

**ENABLE Delphi bulletin week 15-21 November**

**Progress overview:**

Over **200 panelists** from **over 30 European countries** are participating in this process, from **5 areas of expertise: researchers, clinicians, policy makers, patient representatives, technologies developers**. The platform manages responses anonymously and gives us general feedback on participation; **25 panelists have already completed the survey once,** and many others have accessed the platform and initiated the survey.

We know that completing this survey requires quality thinking time, which is a valuable resource in our busy lives, often in short supply given the many responsibilities. We will be waiting patiently for you all to find this time in the next weeks. The survey will be **active for the next 5 weeks**, and you are invited to connect at least two times, first to provide your review and second to reconsider your ratings in light of new input by other members. If you have questions about your participation or difficulties accessing the email invitation, the eDelphi platform, or supplementary materials, please contact us at *wg2costenable@gmail.com.* Some panelists reported difficulties with accessing the links to materials, these are now fixed.

**Discussion points:**

For this bulletin, we summarize some points of active debate on the first question of the survey: the **definition of medication adherence technologies**. This definition was elaborated based on 3 established definitions, of 'health technology', 'medication adherence' and 'best practice', and it will be important for deciding what technologies are within the scope of the repository – an open question, with no obvious answers. The discussions among panelists go at the heart of the matter:

a) should we say **'as agreed'** or **'as prescribed'** – one panelist proposes 'as prescribed' as in the ABC definition, as agreement was considered part of the prescription process in the elaboration of the original definition and missalignment of definitions might lead to confusion; other panelists prefer 'as agreed' for different reasons including valuing the patient perspective in adherence, considering over-the-counter medication (not prescribed), considering agreement over dose adjustment depending on side effects or evolution of symptoms.

b) What are **'procedures'** and **'systems'**; should we rather say **'interventions'**? – several panelists highlighted the ambiguity of these terms, and the need for further precisions; this choice of words is seen as essential for the scope of the future repository. 'Procedure' is seen by one panelist as broadening too much the scope of the repository. One panelist proposes the term 'intervention' to replace 'procedure'.

c) Should we say **'to support patients to take medication',** or… one panelist points to the need to mention also the information collection (measurement?) aspect of technologies; another proposes to use the term 'to enhance' or 'to optimize' medication adherence.

d) What should be the **role of evidence** in the repository? – some panelists consider too restrictive to limit the repository only to 'evidence-based' technologies. Some highlight the difficulty of setting evidence 'thresholds'. One panelist suggests mentioning evidence in the label (Evidence Based MATech) if the focus is such. Two panelists indicate that some practices or apps could be in use without evidence before any evaluation being performed.

e) Several other interesting points are raised on how to **take into account the patient's understanding of their treatment**, broadening the scope to **consider also adherence to lifestyle recommendations,** etc.

What is your view on these topics? Your thoughts will be very valuable for us to steer the next steps in this process, particularly on specifying the repository scope and the criteria for including technologies. You will find further explanations in the supplementary material here. The discussions on the platform will help us set up further work on these important choices. Do provide arguments for your statements, such as examples, or references to policy or research documents.

Many thanks for your time reading this bulletin! We will be back with further summaries next week, and read survey responses regularly to discuss them among our team and adjust course in light of your advice.

Best regards,

Alexandra Dima and Urska Nabergoj-Makovec

On behalf of the ENABLE WG2 Repository Development Team

**ENABLE Delphi bulletin week 22-28 November**

***Progress overview:***

Until now, **60 panellists from 27 countries have started the survey, and 30 panellists have already completed it** at least once. Some of you have just recently received our invitation, as we continue to extend the reach of this consultation and resend invitations that might have gone unnoticed. Thank you for dedicating time to this consultation, your comments on the platform are very valuable for us and will guide the development of the repository and user training.

The **survey will be active for the next 4 weeks**, in which you are invited to connect at least two times, first to provide your review and second to reconsider your ratings in light of new input by other members. For any **questions about your participation** or the information provided on the eDelphi platform, we are available at [**wg2enablecost@gmail.com**](mailto:wg2costenable@gmail.com).

***Discussion points:***

We summarize here new feedback on the MATech definition, **‘completeness of proposed framework’** and the 1_st_ domain, **Product and Provider Information.** The next bulletins will focus on the 2_nd_ and 3_rd_ domains of the attribute framework, which have already attracted valuable comments.

**MATech definition:** Discussions regarding the clarity and appropriateness of the definition continue, and several suggestions have been added for alternative wording and delimiting, narrowing or widening the scope of the repository. Please add your comments, we will perform a detailed analysis of this discussion which will inform decisions on the focus of the repository within the ENABLE network!

**Framework completeness**: Several participants noted that the framework appears complete and welcomed the detailed information provided. Some panellists highlighted missing attributes, such as reimbursement authority, additional routes of administration, cost details, patient expectations, professional behaviours and behaviour determinants, which we will consider carefully for inclusion in the framework. Some raised concerns regarding the fit of the framework for technologies other that products with electronic components, such as bio- and nano-technologies under development.

Some panellists noted that the framework is too complex and difficult to review in the format used by the supporting documents. Therefore, for panellists who prefer to review attribute groups sequentially we advise to go directly to the next section of the survey; those who prefer starting with an overview of the framework may want to explore the supporting documents at this time. We appreciate further feedback to improve the presentation and comprehensiveness of the framework.

**Product and provider information (D1.1.)**. This domain includes basic information on the product and its manufacturer/developer. The current discussions refer to the clarity and completeness of these attributes.

a) What is a **‘product’**? Some panellists pointed out that a product can also be a combination of devices, procedures and/or systems. In this case, it becomes unclear which provider information should be described in the repository. Some panellists raised concerns about whether ‘procedures’ and ‘systems’ could be described as ‘products´. Should the repository include only technologies that can be described as products? Should it allow description of technologies with multiple components? These are open questions on which we would welcome all suggestions, for improved definitions or ways of describing technologies!

b) A**dditional attributes proposed.** Several panellists recommended to include attributes for providers working in a partnership or consortium, or developers of open-source/ community-driven products. A panellist proposed the addition of a ‘product ancestry’ attribute for products that are ‘improved versions’ or a ‘fusion’ of previous products.

What is your view on these topics? We are grateful for your active discussion and input. Your thoughts will be very valuable for us to steer the next steps in this process. To facilitate comprehension of your perspective, please provide arguments for your statements, such as examples, or references to policy or research documents.

Many thanks for your time reading this bulletin. We will review comments regularly, discuss them among our team, and summarize them during the next weeks to keep you all updated with the progress.

Best regards,

Frederik Haupenthal, Alexandra Dima and Urska Nabergoj-Makovec

**ENABLE Delphi bulletin week 29 Nov - 5 Dec**

***Progress overview:***

The participation to the survey continues to increase. Until now, **86 panellists from 28 countries have started the survey, and 46 panellists have already completed it** at least once. Some of you have just recently received our invitation, as we continue to extend the reach of this consultation and resend invitations that might have gone unnoticed. Thank you for dedicating time to this consultation, your comments on the platform are very valuable for us and will guide the development of the repository and user training.

The **survey will stay active for the next 3 weeks**. You are invited to connect at least two times, first to provide your review and second to reconsider your ratings in light of new input by other members. For any **questions about your participation** or the information provided on the eDelphi platform, we are available at [**wg2enablecost@gmail.com**](mailto:wg2costenable@gmail.com).

***Discussion points:***

This bulletin focuses on the second domain, `**Medication adherence descriptors**`. Several attribute groups are under lively discussion, which will be key for structuring a comprehensive repository that will be of practical value for users. We highlight below several points made on ‘**Target use scenarios’ (D2.1), health condition (D2.2),** ‘**medication regimen**’ **(D2.3)** and ‘**phases of medication adherence’ (D.2.4.1).**

a)     Who might be using technologies for medication adherence?

Regarding ‘**target use scenarios’** (D2.1), some participants advised that, in the adherence support scenario, the ***professional health, social care provider and health (system) manager*** attributes should further detail specific professions or types of user. Others indicated that the ‘**caregiver**’ attribute should specify if the caregiver is a professional or a family member and proposed to detail professions as in the section describing intervention providers (D.2.4.2B4). Several panellists suggested additional attributes: caregiver health literacy, patient time since diagnosis, healthcare provider years of experience. One panellist questioned the label ‘person in the healthcare environment’ for the self-management scenario, which usually happens outside this environment. We welcome your views and suggestions on the level of detail appropriate for this attribute group and missing attributes.

b)     For which **health condition** is the technology applicable?

Some panellists noted that technologies are not limited to a specifically targeted health condition and questioned the appropriateness of this categorisation for adherence research. In contrast, it was also argued that adherence interventions are usually developed for specific health conditions therefore these attributes would be useful. Some panellists suggested that there needs to be the possibility to specify that a technology is applicable to multiple conditions – we confirm that the platform will give the option of selecting multiple categories. One panellist questioned the use of the term ‘health condition’ as it is sometimes an euphemism for ‘disease’. Others proposed the use of the ATC classification instead, or additional attributes like ‘vaccines’, ‘addiction’ or ‘pregnancy’, or more detailed definitions. What are your opinions about the specification of health conditions for adherence technologies?

c)     How should the ‘**medication regimen**’ be described?

The current description provides multiple attributes regarding the medication regimen the patient aims to follow. However, several panellists indicated some missing attributes, such as ‘palliative’ intention and ‘as-required’ type. It was noted that short-/or long-term treatments should be defined by a specific timeframe, and an ‘additional recommendation’ added, e.g. intake with food, inhaler technique. One panellist pointed out that selecting multiple medications monitored’ would mean specifying a ‘prescribed dosing frequency’ for each medication. What are your views on how this section could be efficiently described?

d)     How should the ‘**phases of medication adherence’** be defined?

The attributes and definitions proposed were formulated following the ABC taxonomy and rephrasing to fit the attribute definition format across the framework. For several panellists the wording did not sufficiently match the ABC definitions. Several recommended following more closely the original formulation for improved comprehension and standardisation. Do you think the definitions should follow exactly the ABC taxonomy? Please let us know your views.

This summary includes several examples of how the eDelphi process contributes to improving the repository: your comments have already been of great value in changing the perspective on the proposed structure and revealing the need for improvement in several descriptions. They continue to be very valuable for us to steer the next steps in this process. To facilitate comprehension of your perspective, please provide arguments for your statements, such as examples, or references to policy or research documents.

 Many thanks for your time reading this bulletin. We will review comments regularly, discuss them among our team, and summarize them during the next weeks to keep you all updated with the progress.

 Best regards,

Frederik Haupenthal and Alexandra Dima

**ENABLE Delphi bulletin week 6 - 12 December**

***Progress overview:***

To date, **102 panellists from 30 countries have started the survey and 59 have already completed it** at least once. Thank you for taking time to contribute to this consultation, we appreciate that the end of the year is a busy period. We continue to invite new participants who expressed interest, and we plan to keep the **survey active until the end of the year**, to give everyone time to connect and reflect on the proposed repository structure and the ongoing discussions. You are invited to connect at least two times, first to provide your review and second to reconsider your ratings in light of other participants’ input. For any **questions about your participation** or the information provided on the eDelphi platform, we are available at [wg2enablecost@gmail.com](mailto:wg2enablecost@gmail.com).

***Discussion points:***

This bulletin will continue to summarize discussions on `**Medication adherence descriptors`** and highlights important points made on ‘**Monitoring/measurement’ (D2.4.2.A)** and ‘**Support/intervention**‘ (**D2.4.2.B)** and related attribute groups

a)     Are monitoring and support types of adherence management?

According to the ABC taxonomy, the management of adherence is the ‘process of monitoring and supporting patient’s adherence´. Thus, adherence technologies may be applied to either monitor or support adherence, or both. The repository proposes to make this distinction by including attributes referring to ‘Monitoring/measurement` and ‘Support/intervention’. Several panellists commented that the definitions of both types were confusing and insufficiently in line with ABC definitions. The terms ‘support’ and ‘intervention’ were perceived as synonymous and using both terms was confusing for for some panellists. What is your view on the use of these two terms for describing adherence technologies?

b)     How should measurement of adherence be described?

In this group of attributes, we aimed to capture all measurement methods and targets that technologies could include. Some panellists generally mentioned additional methods to include, e.g. ‘bloodspot assays’, ‘dose counters for inhalers’, ‘electronic recordings of dose intake’, ‘weighting of returned creams’ or ‘self-report via apps’. Some panellists questioned the inclusion of measurement targets other than adherence within the adherence measurement group and suggested ways of keeping them separate. Several additional targets were proposed, e.g. clinical outcome, patient reported and economic outcome measures, as well as making further distinctions to describe some methods and targets. Some panellists recommended the use of more accessible language. How should we best capture adherence measurement by technologies? We are grateful for further recommendations.

c)     How are interventions delivered and how they support behaviour?

In these groups of attributes, we aimed to capture the characteristics of interventions as described by available classifications. Several suggestions for improvement referred to missing attributes (e.g. self-enrolment programs), use of technical terms (e.g. capability versus literacy), examples used (e.g. smart boxes as not ‘wearable’, brochure as ‘printed media’). Some proposed ways in which to specify if several attributes, or none, apply to specific technologies., and how these terms should be introduced and further explained to users to ensure clarify. These attributes are the core of what makes adherence technologies useful to users – we are keen to hear your views and suggestions on this!

Discussions on these topics continue to inform necessary improvements on several attribute groups, so that they are more appropriate and better understood by stakeholders with different backgrounds – which will be our focus in the next steps of this process. Please consider adding your views and supporting with examples or references to policy or research documents, to help us understand your perspective.

Thank you for reading this bulletin. We continue to review comments and summarize them in the remaining bulletins.

 Best regards,

Frederik Haupenthal and Alexandra Dima

## ENABLE Delphi bulletin week 13 - 19 December 2021

***Progress overview:***
Participation continues to increase, so far 113 panellists from 31 countries have initiated the survey and 64 have completed it at least once. Several participants told us they aim to visit the platform over the winter break. We will keep the survey active until early 2022 to give everyone time to connect and reflect on the proposed repository structure and the ongoing discussions. For any questions, you can reach us at wg2enablecost@gmail.com.

***Discussion points:***
In this bulletin we move to the 3_rd_ and last domain, `Evaluation and Implementation` (D3), and summarize your comments on ‘Quality indicators´ (D3.1.).

a) Is ISO-certification relevant?

For several panellists ISO-certification is not sufficient to indicate quality. Panellists indicated that research on health technology should address `quality, security and efficacy` and that ISO may be at company-level, not product-level, and may be addressed after a MATech has been ‘found fit for purpose’. Where do you see ISO-certification in the context of MATech evaluation?

b) What evidence of scientific evaluation should be available?

Most panellists indicated that this attribute group is most relevant. Additional attributes were suggested to capture effectiveness, outcome measures, safety, context of scientific evaluation. One panellist proposed to capture links of publications (PubMed-IDs) or clinical trial numbers.  Do you have any further suggestions for this section?

c) How should development standards be described?

While most panellists agreed on the importance of these attributes, several pointed to the need to simplify the language, particularly from patient´s perspective. One panellist noted the need for ‘iterative improvement’ given the constantly changing standards. One proposal was to consider ‘principles of implementation science’ (contextual analysis, stakeholder involvement) in this attribute group. Implementation attributes are described separately in D3.2., but should we already refer to such indicators in this section? We are open to your opinions on these points.

d) How should technical standards be described?

Data protection was a crucial and sensitive topic for the panellists. Suggestions of additional attributes referred to describing the country of data storage, data sharing features, the capacity of systems for future (automatic) updates. One panellist proposed considering features assessed in DOI:10.1136/bmj.n1248. All suggestions on relevant technical standards are welcome!

e) Which policy-related quality indicators should be assessed?

Some panellists proposed to specify the ‘perspective’ (e.g. a payer or societal perspective) of cost-effectiveness analyses, the analytic period, and the measures for evaluation. One panellist indicated that cost-effectiveness should be performed against ‘no’ or ‘another intervention’. Another comment referred to either also detailing cost-utility, cost-benefit, etc., or providing only higher-level descriptions. One panellist questioned whether this type of information, although clear and relevant, should be included in the repository. What are your thoughts on these matters?

f) Which use-related quality indicators should be assessed?

Suggestions for additional attributes referred to size of writing or culturally sensitive validated pictograms within readability, choice of colour and language simplification within customization, accessibility, and user experience dimensions. One panellist noted that ‘usability’ is conceptually close to ‘usability tests’ in the ‘User-centred design process’ and proposed grouping them together. We remain open to other suggestions.

Your input is key for obtaining a meaningful set of quality indicators for adherence technologies! Please continue to think along with us and consider adding your views and supporting with examples or references to policy or research documents, to help us understand your perspective on these topics.

Thank you for time reading this bulletin. We will be reviewing new input over the next 2 weeks and summarize them in the next (and last) bulletin early January.

Have a safe and peaceful holiday season, and all the best for 2022!

Frederik Haupenthal and Alexandra Dima

## ENABLE Delphi bulletin January 2022

***Progress Overview:***

Until now, 120 participants from 31 countries have initiated the survey and 72 have already completed it at least once. A big thank you to those of you who dedicated time in the past two weeks to this consultation!
We are very thankful for any further contributions and appreciate the guidance you have provided so far for this ENABLE initiative. Participants who already accepted, or will accept, our invitation and access the online platform until January 15 will be given the option (via a separate message) to register their name and professional affiliation via a separate link, should they wish to be acknowledged in the dissemination of the results of this work.
For any last questions about this consultation, or if you are interested in the next steps of the repository development, please contact us at wg2enablecost@gmail.com.

***Discussion points:***

In this last bulletin we continue to review the discussion on the 3rd and last domain `**Evaluation and Implementation**` (D3) and summarize your comments on `**Implementation Outcomes and Strategies`(D3.2)**:

a) How should implementability of technology be characterised?

Several panellists indicated several additional implementation outcomes that should be considered, e.g. readiness, affordability, and confidence of users/providers about MATechs. It was also noted that descriptions about the `reachability` of a technology should be added to indicate whether the technology is actually received by populations that may benefit. A panellist proposed to evaluate ‘reproducibility’ in different patient cohorts as implementation outcome. What is your opinion about these attributes? Please provide further suggestions of potentially missing implementation outcomes.

b) What are strategies for the implementation of MATechs?

To add to the proposed strategies, panellists argued that maintenance of a product or technology requires a ‘continued evaluation’ and ‘access to technology updates’, and that implementation of technology needs to be connected conceptually with the Learning Health System approach as it is an iterative process. A panellist pointed out that implementation is also influenced by the relationship between care provider and patient. Do you think that all strategies relevant for implementing MATechs are included and adequately defined? We remain open for suggestions.

We also reviewed your comments on ‘**’completeness of the proposed framework**’ which we summarized in the first bulletins. In general, the framework was perceived as complete, despite the graphical illustration remaining complex to navigate. However, several panellists mentioned one important missing descriptor: the ‘digital health - or eHealth literacy’ of patients; this factor was seen as crucial for engagement with digital solutions. What do think about this point? Please provide any last suggestions about missing descriptors.

Until now, your commitment to this consultation and the on-going discussions have been instrumental for the success of this survey and for improving the repository. Please continue to consider adding your perspectives during these last days of consultation and support your opinions with references to policy or research documents.

Thank you for your time reading this bulletin. We will be reviewing the input during the next days and conduct a detailed analysis in the following weeks, which will be described in openly accessible dissemination materials and represent the basis of the repository soon to be accessible at https://enableadherence.eu/.

All the best for 2022!

Frederik Haupenthal and Alexandra Dima

On behalf of the ENABLE WG2 Repository Development Team

Example layout


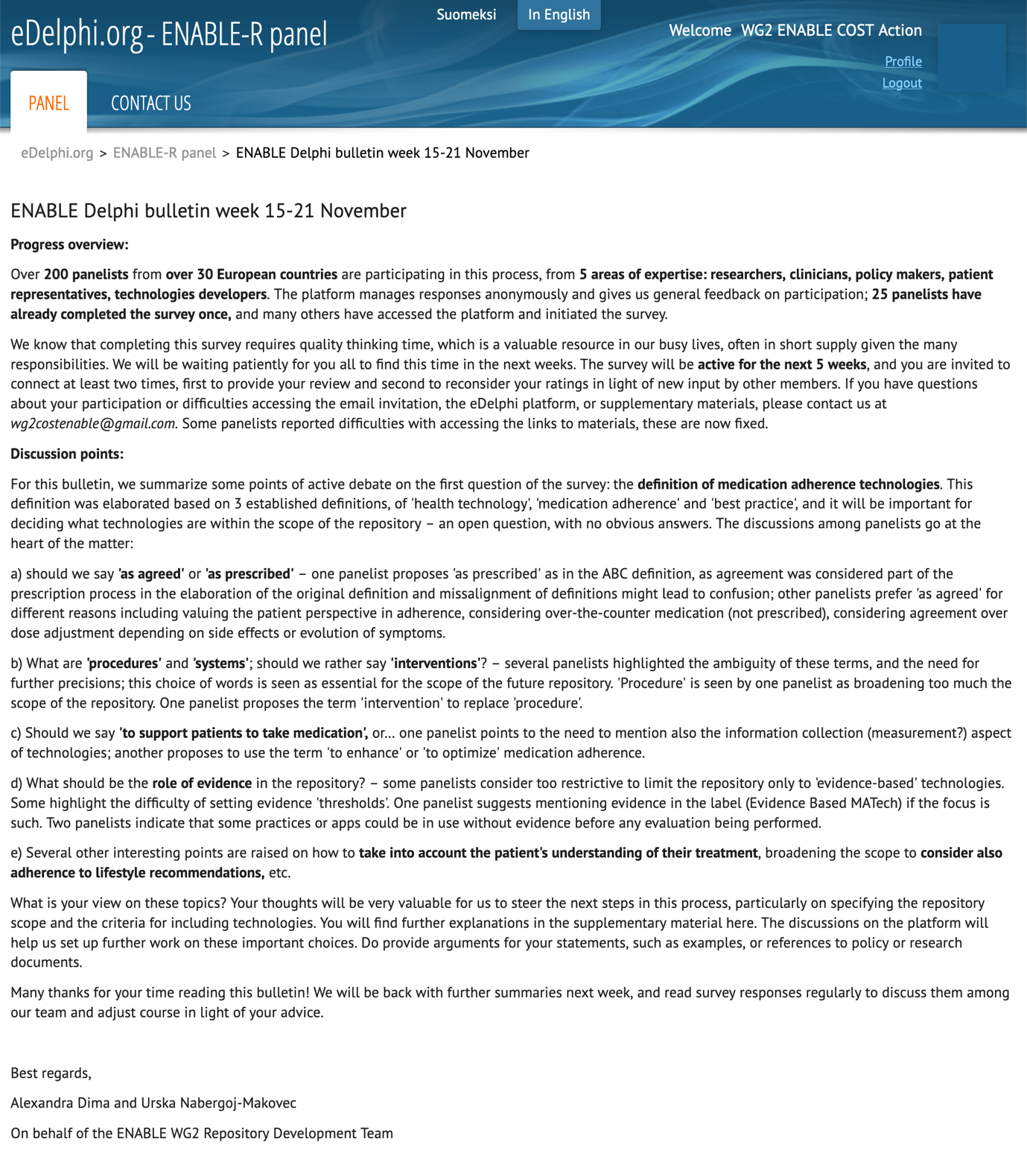

Supplement: Multimedia Appendix 1 [file jmir_v27i1e59738_app1.doc]
