# Supplementary material for: Stakeholder Consensus on an Interdisciplinary Terminology to Enable the Development and Uptake of Medication Adherence Technologies Across Health Systems: Web-Based Real-Time Delphi Study
Source: J Med Internet Res. 2025 Mar 25;27:e59738. doi: 10.2196/59738 (PMC11979531; doi:10.2196/59738)

Stakeholder consensus on an interdisciplinary terminology to enable development and uptake of medication adherence technologies across health systems: an online real-time Delphi study

# Multimedia Appendix 1. Relevance and clarity ratings for full sample and stakeholder subgroups

Table S1. Descriptive statistics for relevance ratings (N=83)

| Attribute | Median | 30-70th percentile | IPR | IPRAS | DI |
| --- | --- | --- | --- | --- | --- |
| Product and provider information | 7.04 | 6.55 - 7.70 | 1.15 | 5.54 | 0.21 |
| Target use scenario | 7.66 | 6.99 - 8.01 | 1.02 | 6.10 | 0.17 |
| Target health conditions | 7.11 | 6.44 - 7.97 | 1.54 | 5.66 | 0.27 |
| Medication regimen | 7.34 | 7.02 - 7.76 | 0.74 | 5.93 | 0.13 |
| Medication adherence phase | 7.44 | 6.93 - 7.85 | 0.93 | 5.93 | 0.16 |
| Medication adherence measurement | 7.36 | 6.99 - 7.76 | 0.78 | 5.91 | 0.13 |
| Medication adherence intervention | 7.02 | 6.50 - 7.62 | 1.12 | 5.44 | 0.21 |
| Intervention mode of delivery | 7.39 | 6.72 - 7.96 | 1.24 | 5.85 | 0.21 |
| Target behaviour determinants | 7.46 | 6.79 - 7.76 | 0.97 | 5.77 | 0.17 |
| Behaviour change techniques | 7.49 | 7.12 - 7.91 | 0.79 | 6.12 | 0.13 |
| Intervention provider | 7.35 | 7.00 - 7.86 | 0.86 | 6.00 | 0.14 |
| Intervention setting | 7.25 | 6.75 - 7.65 | 0.90 | 5.65 | 0.16 |
| ISO certification | 6.34 | 5.50 - 7.24 | 1.74 | 4.41 | 0.39 |
| Scientific evaluation | 7.20 | 6.95 - 7.86 | 0.91 | 5.96 | 0.15 |
| Development standards | 7.05 | 6.56 - 7.43 | 0.87 | 5.34 | 0.16 |
| Technological standards | 7.25 | 6.91 - 7.54 | 0.63 | 5.68 | 0.11 |
| Research-related quality | 7.40 | 6.99 - 7.62 | 0.62 | 5.81 | 0.11 |
| Policy-related quality | 7.23 | 6.91 - 7.67 | 0.76 | 5.78 | 0.13 |
| Use-related quality | 7.49 | 7.01 - 8.02 | 1.01 | 6.12 | 0.17 |
| Implementation outcomes | 7.58 | 7.11 - 7.98 | 0.87 | 6.16 | 0.14 |
| Implementation strategies | 7.55 | 7.05 - 7.95 | 0.89 | 6.10 | 0.15 |

Table S2. Descriptive statistics for clarity ratings (N=83)

| Attribute | Median | 30-70th percentile | IPR | IPRAS | DI |
| --- | --- | --- | --- | --- | --- |
| Product and provider information | 7.03 | 6.48 - 7.58 | 1.10 | 5.39 | 0.20 |
| Target use scenario | 6.98 | 6.07 - 7.56 | 1.49 | 5.07 | 0.29 |
| Target health conditions | 7.01 | 6.35 - 7.78 | 1.44 | 5.45 | 0.26 |
| Medication regimen | 6.94 | 6.10 - 7.56 | 1.46 | 5.09 | 0.29 |
| Medication adherence phase | 6.07 | 4.86 - 7.17 | 2.30 | 3.87 | 0.59 |
| Medication adherence measurement | 6.37 | 4.80 - 6.97 | 2.18 | 3.68 | 0.59 |
| Medication adherence intervention | 5.67 | 4.66 - 6.61 | 1.95 | 3.30 | 0.59 |
| Intervention mode of delivery | 6.92 | 6.11 - 7.52 | 1.41 | 5.07 | 0.28 |
| Target behaviour determinants | 6.61 | 5.97 - 7.19 | 1.22 | 4.72 | 0.26 |
| Behaviour change techniques | 6.93 | 5.86 - 7.40 | 1.54 | 4.8 | 0.32 |
| Intervention provider | 7.00 | 6.24 - 7.57 | 1.33 | 5.21 | 0.25 |
| Intervention setting | 7.17 | 6.48 - 7.67 | 1.19 | 5.47 | 0.22 |
| ISO certification | 7.46 | 6.16 - 8.03 | 1.87 | 5.49 | 0.34 |
| Scientific evaluation | 7.52 | 7.01 - 7.96 | 0.95 | 6.07 | 0.16 |
| Development standards | 7.17 | 6.82 - 7.64 | 0.82 | 5.70 | 0.14 |
| Technological standards | 7.54 | 7.06 - 7.82 | 0.76 | 6.01 | 0.13 |
| Research-related quality | 7.42 | 6.91 - 7.84 | 0.92 | 5.91 | 0.16 |
| Policy-related quality | 7.33 | 6.75 - 7.74 | 0.99 | 5.72 | 0.17 |
| Use-related quality | 7.65 | 7.12 - 8.15 | 1.03 | 6.30 | 0.16 |
| Implementation outcomes | 7.67 | 7.20 - 8.06 | 0.85 | 6.30 | 0.14 |
| Implementation strategies | 7.58 | 7.19 - 8.02 | 0.84 | 6.26 | 0.13 |

Figure S1. Median ratings with Interpercentile Range for relevance and clarity for attribute clusters per stakeholder subgroup: 2.a. Research and Education (N=50); 2.b. Healthcare Professional (N=45); 2.c. Policy and Decision Making (N=19); 2.d. Patient representative (N=18); 2.e. Technology developer (N=24).


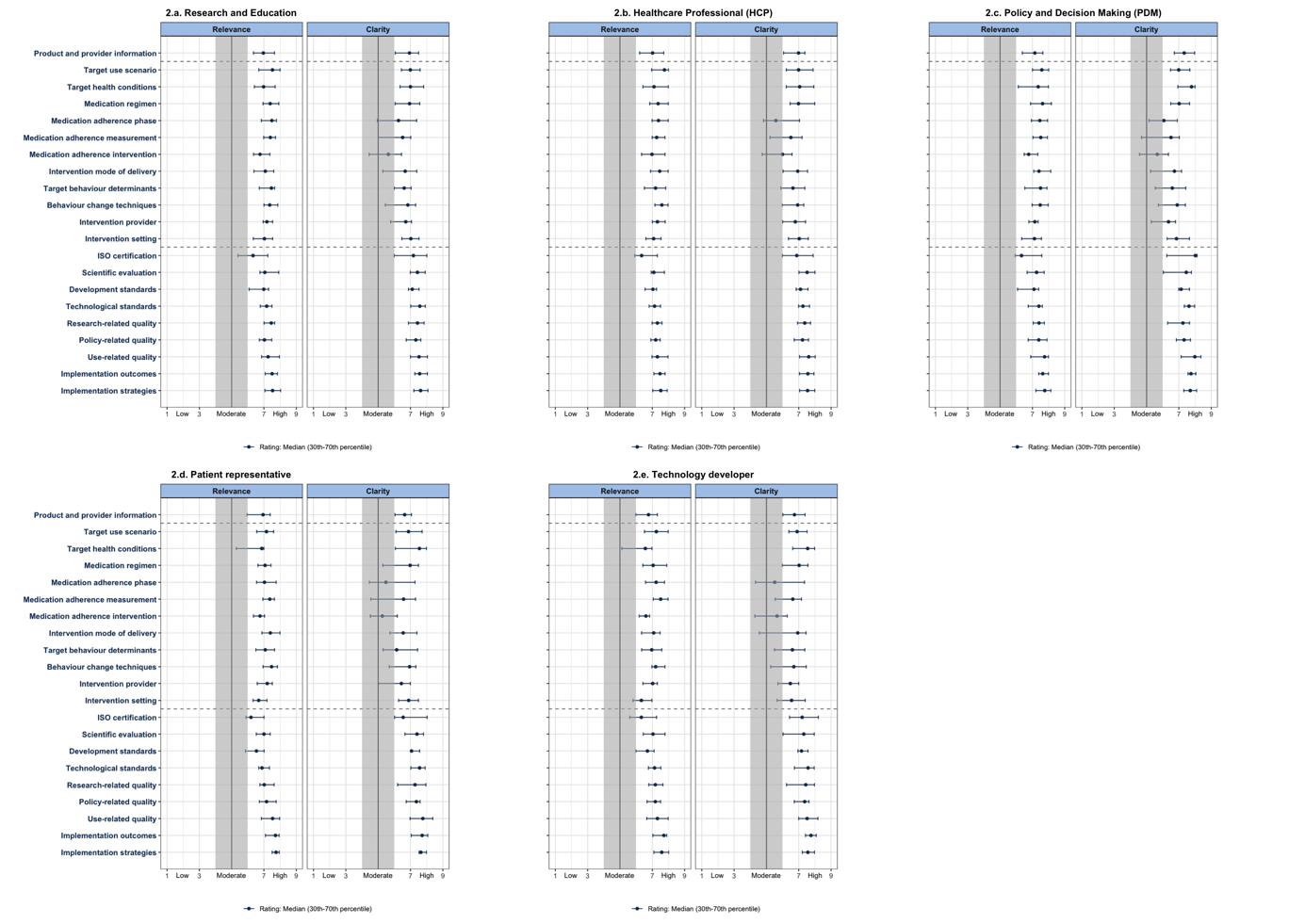

Supplement: Multimedia Appendix 3 [file jmir_v27i1e59738_app3.docx]
