# Supplementary material for: Stakeholder Consensus on an Interdisciplinary Terminology to Enable the Development and Uptake of Medication Adherence Technologies Across Health Systems: Web-Based Real-Time Delphi Study
Source: J Med Internet Res. 2025 Mar 25;27:e59738. doi: 10.2196/59738 (PMC11979531; doi:10.2196/59738)
Supplement: Multimedia Appendix 4 [file jmir_v27i1e59738_app4.zip › ENABLE RT Delphi MA3 - quantitative data report.html]

ENABLE Delphi study analysis


# ENABLE Delphi study analysis

#### 2024-04-28

This report presents the analysis of the ENABLE Delphi survey conducted between October 2021 and January 2022 within the Working Group 2 of the ENABLE COST action. The survey aimed to collect stakeholders feedback on the proposed structure for an online repository of medication adherence technologies. The report includes the following analyses:

1. **Descriptive statistics** were performed to characterize the sample (and each stakeholder subgroup) regarding profession, years of experience, age, gender and country.
2. The **level of agreement** on relevance, clarity and completeness was described based on several criteria representing different levels of agreement and consequently carrying different weights in these decisions.

- the Interpercentile Range Adjusted for Symmetry (IPRAS), an analysis technique from the RAND-UCLA Appropriateness Method (RAM).
- the disagreement index (DI) was calculated as a ratio between the Interpercentile Range (IPR) and IPRAS. A DI above 1 (i.e., IPR above IPRAS) indicates that disagreement exists. The IPR was calculated using the 30th to 70th percentile. IPRAS for the 9-points Likert scale was calculated according to the formula presented in the RAM User Manual.
- the median and DI defined different levels of agreement and steer the decisions about the repository structure. For relevance ratings:
- items with the median of 7-9 and no disagreement were considered as relevant and mandatory.
- items with the median of 4-6 or disagreement were considered as optional.
- items with the median of 1-3 and no disagreement, were considered not relevant and candidates for exclusion. Median ratings were rounded to the nearest integers (e.g., 6.5 or 3.5 or above were assigned to 7 or 4). Stakeholders’ responses per question were summarized using descriptive statistics. For clarity ratings, the above criteria were applied as (i) sufficiently clear to remain unchanged; (ii) optional changes and (iii) candidates for rephrasing. Panellist comments in the open text fields were analysed qualitatively in a separate document, using content analysis.

2. **Subgroup analyses** per stakeholder group examined variation in opinions and potential differences among subgroups. The same agreement criteria were applied and descriptive statistics were stratified by stakeholder group. The reliability of ratings per question within stakeholder group was reported by calculating the intraclass correlation coefficient (ICC). The ICC calculation was based on the two-way random model, considering type (average measures) and definition of relationship (consistency). ICC > 0.70 indicated moderate to good reliability.
3. An **analysis of process indicators** described how stakeholders’ responses evolved through iterations and how consensus or certain level of agreement has formed. Stability of response presented the consistency of responses within the study period and between respondent group stability, which is considered a necessary precondition for determining the level of agreement or if consensus was achieved. We used the coefficient of quartile variation (CQV) as a descriptive measure of response stability. CQV was calculated over all participants (CQVtotal) and within the same stakeholder group (CQVsub) to account for expected higher variation in response between different stakeholder groups. A CQVtotal less than 30% and CQVsub less than 15% was considered as stable response.

# Participation rates, sample size and missing data analysis

The final sample includes 174 participants from 32 countries who connected at least once, of which 117 participants started the survey. Of these, 83 participants from 27 countries have completed sociodemographic data and at least 75 percent of the rating questions. The descriptive statistics are presented for the latter group in Table 1, and for all participants who started the survey in Table 2.

Table 1: Descriptive statistics of sample characteristics

|  | Values | Frequencies (%) |
| --- | --- | --- |
| n |  | 83 |
| Q1.Gender (%) | Female | 54 (65.1) |
|  | Male | 29 (34.9) |
| Q2.Age (%) | 18-30 | 2 ( 2.4) |
|  | 31-40 | 22 (26.5) |
|  | 41-50 | 28 (33.7) |
|  | 51-60 | 26 (31.3) |
|  | 61-70 | 5 ( 6.0) |
| Q3.Country (%) | Albania | 4 ( 4.8) |
|  | Belgium | 2 ( 2.4) |
|  | Bosnia and Herzegovina | 5 ( 6.0) |
|  | Bulgaria | 2 ( 2.4) |
|  | Croatia | 4 ( 4.8) |
|  | Cyprus | 1 ( 1.2) |
|  | Czech Republic | 1 ( 1.2) |
|  | Denmark | 1 ( 1.2) |
|  | Finland | 1 ( 1.2) |
|  | France | 1 ( 1.2) |
|  | Germany | 2 ( 2.4) |
|  | Greece | 3 ( 3.6) |
|  | Hungary | 2 ( 2.4) |
|  | Italy | 9 (10.8) |
|  | Latvia | 6 ( 7.2) |
|  | Malta | 2 ( 2.4) |
|  | Netherlands | 3 ( 3.6) |
|  | Norway | 4 ( 4.8) |
|  | Poland | 2 ( 2.4) |
|  | Portugal | 2 ( 2.4) |
|  | Romania | 3 ( 3.6) |
|  | Serbia | 3 ( 3.6) |
|  | Slovenia | 1 ( 1.2) |
|  | Spain | 6 ( 7.2) |
|  | Sweden | 4 ( 4.8) |
|  | Switzerland | 6 ( 7.2) |
|  | United Kingdom | 3 ( 3.6) |
| Q4.Education (%) | Bachelor | 3 ( 3.6) |
|  | Doctorate (PhD) | 42 (50.6) |
|  | High school diploma | 3 ( 3.6) |
|  | Master | 23 (27.7) |
|  | Speciality Degree (healthcare) | 12 (14.5) |
| Q5.Expertise (%) | Computer Science / Software Engineering | 4 ( 4.8) |
|  | Data Science / Statistics | 4 ( 4.8) |
|  | Economy / Management | 8 ( 9.6) |
|  | Medicine | 16 (19.3) |
|  | Nursing | 11 (13.3) |
|  | Other | 5 ( 6.0) |
|  | Pharmacy | 33 (39.8) |
|  | Psychology | 2 ( 2.4) |
| research.education (%) |  | 33 (39.8) |
|  | 10 to <15 years experience | 7 ( 8.4) |
|  | 15 to <20 years experience | 8 ( 9.6) |
|  | 20 to <30 years experience | 12 (14.5) |
|  | 5 to <10 years experience | 11 (13.3) |
|  | less than 5 years experience | 10 (12.0) |
|  | more than 30 years experience | 2 ( 2.4) |
| healthcare.practitioner (%) |  | 38 (45.8) |
|  | 10 to <15 years experience | 10 (12.0) |
|  | 15 to <20 years experience | 8 ( 9.6) |
|  | 20 to <30 years experience | 16 (19.3) |
|  | 5 to <10 years experience | 4 ( 4.8) |
|  | less than 5 years experience | 4 ( 4.8) |
|  | more than 30 years experience | 3 ( 3.6) |
| policy.decision.maker (%) |  | 64 (77.1) |
|  | 10 to <15 years experience | 3 ( 3.6) |
|  | 15 to <20 years experience | 2 ( 2.4) |
|  | 5 to <10 years experience | 1 ( 1.2) |
|  | less than 5 years experience | 13 (15.7) |
| patient.perspective (%) |  | 65 (78.3) |
|  | 10 to <15 years experience | 2 ( 2.4) |
|  | 15 to <20 years experience | 1 ( 1.2) |
|  | 20 to <30 years experience | 2 ( 2.4) |
|  | 5 to <10 years experience | 3 ( 3.6) |
|  | less than 5 years experience | 6 ( 7.2) |
|  | more than 30 years experience | 4 ( 4.8) |
| Health.IT.specialist (%) |  | 59 (71.1) |
|  | 10 to <15 years experience | 4 ( 4.8) |
|  | 15 to <20 years experience | 2 ( 2.4) |
|  | 20 to <30 years experience | 3 ( 3.6) |
|  | 5 to <10 years experience | 7 ( 8.4) |
|  | less than 5 years experience | 8 ( 9.6) |
| res.ed.nr (%) | 0 | 33 (39.8) |
|  | 1 | 50 (60.2) |
| hcp.nr (%) | 0 | 38 (45.8) |
|  | 1 | 45 (54.2) |
| policy.nr (%) | 0 | 64 (77.1) |
|  | 1 | 19 (22.9) |
| patient.nr (%) | 0 | 65 (78.3) |
|  | 1 | 18 (21.7) |
| healthIT.nr (%) | 0 | 59 (71.1) |
|  | 1 | 24 (28.9) |
| exp.nr (%) | 1 | 49 (59.0) |
|  | 2 | 16 (19.3) |
|  | 3 | 7 ( 8.4) |
|  | 4 | 1 ( 1.2) |
|  | 5 | 10 (12.0) |

Table 2: Descriptive statistics of sample characteristics

|  | Values | Frequencies (%) |
| --- | --- | --- |
| n |  | 117 |
| Q1.Gender (%) | Female | 74 (63.2) |
|  | Male | 43 (36.8) |
| Q2.Age (%) | 18-30 | 4 ( 3.4) |
|  | 31-40 | 28 (23.9) |
|  | 41-50 | 43 (36.8) |
|  | 51-60 | 35 (29.9) |
|  | 61-70 | 7 ( 6.0) |
| Q3.Country (%) | Albania | 5 ( 4.3) |
|  | Belgium | 3 ( 2.6) |
|  | Bosnia and Herzegovina | 6 ( 5.1) |
|  | Bulgaria | 2 ( 1.7) |
|  | Croatia | 4 ( 3.4) |
|  | Cyprus | 3 ( 2.6) |
|  | Czech Republic | 2 ( 1.7) |
|  | Denmark | 3 ( 2.6) |
|  | Finland | 4 ( 3.4) |
|  | France | 2 ( 1.7) |
|  | Germany | 3 ( 2.6) |
|  | Greece | 4 ( 3.4) |
|  | Hungary | 3 ( 2.6) |
|  | Ireland | 1 ( 0.9) |
|  | Italy | 10 ( 8.5) |
|  | Latvia | 8 ( 6.8) |
|  | Luxembourg | 1 ( 0.9) |
|  | Malta | 3 ( 2.6) |
|  | Netherlands | 4 ( 3.4) |
|  | Norway | 7 ( 6.0) |
|  | Poland | 3 ( 2.6) |
|  | Portugal | 5 ( 4.3) |
|  | Romania | 3 ( 2.6) |
|  | Serbia | 4 ( 3.4) |
|  | Slovenia | 4 ( 3.4) |
|  | Spain | 6 ( 5.1) |
|  | Sweden | 4 ( 3.4) |
|  | Switzerland | 7 ( 6.0) |
|  | United Kingdom | 3 ( 2.6) |
| Q4.Education (%) | Bachelor | 4 ( 3.4) |
|  | Doctorate (PhD) | 64 (54.7) |
|  | High school diploma | 4 ( 3.4) |
|  | Master | 28 (23.9) |
|  | Speciality Degree (healthcare) | 17 (14.5) |
| Q5.Expertise (%) | Computer Science / Software Engineering | 5 ( 4.3) |
|  | Data Science / Statistics | 5 ( 4.3) |
|  | Economy / Management | 11 ( 9.4) |
|  | Medicine | 26 (22.2) |
|  | Nursing | 11 ( 9.4) |
|  | Other | 7 ( 6.0) |
|  | Pharmacy | 48 (41.0) |
|  | Psychology | 3 ( 2.6) |
|  | Sociology | 1 ( 0.9) |
| research.education (%) |  | 43 (36.8) |
|  | 10 to <15 years experience | 10 ( 8.5) |
|  | 15 to <20 years experience | 15 (12.8) |
|  | 20 to <30 years experience | 17 (14.5) |
|  | 5 to <10 years experience | 14 (12.0) |
|  | less than 5 years experience | 14 (12.0) |
|  | more than 30 years experience | 4 ( 3.4) |
| healthcare.practitioner (%) |  | 55 (47.0) |
|  | 10 to <15 years experience | 10 ( 8.5) |
|  | 15 to <20 years experience | 12 (10.3) |
|  | 20 to <30 years experience | 21 (17.9) |
|  | 5 to <10 years experience | 8 ( 6.8) |
|  | less than 5 years experience | 6 ( 5.1) |
|  | more than 30 years experience | 5 ( 4.3) |
| policy.decision.maker (%) |  | 88 (75.2) |
|  | 10 to <15 years experience | 6 ( 5.1) |
|  | 15 to <20 years experience | 5 ( 4.3) |
|  | 5 to <10 years experience | 3 ( 2.6) |
|  | less than 5 years experience | 15 (12.8) |
| patient.perspective (%) |  | 91 (77.8) |
|  | 10 to <15 years experience | 4 ( 3.4) |
|  | 15 to <20 years experience | 1 ( 0.9) |
|  | 20 to <30 years experience | 2 ( 1.7) |
|  | 5 to <10 years experience | 5 ( 4.3) |
|  | less than 5 years experience | 8 ( 6.8) |
|  | more than 30 years experience | 6 ( 5.1) |
| Health.IT.specialist (%) |  | 85 (72.6) |
|  | 10 to <15 years experience | 4 ( 3.4) |
|  | 15 to <20 years experience | 3 ( 2.6) |
|  | 20 to <30 years experience | 3 ( 2.6) |
|  | 5 to <10 years experience | 11 ( 9.4) |
|  | less than 5 years experience | 11 ( 9.4) |
| res.ed.nr (%) | 0 | 43 (36.8) |
|  | 1 | 74 (63.2) |
| hcp.nr (%) | 0 | 55 (47.0) |
|  | 1 | 62 (53.0) |
| policy.nr (%) | 0 | 88 (75.2) |
|  | 1 | 29 (24.8) |
| patient.nr (%) | 0 | 91 (77.8) |
|  | 1 | 26 (22.2) |
| healthIT.nr (%) | 0 | 85 (72.6) |
|  | 1 | 32 (27.4) |
| exp.nr (%) | 1 | 65 (55.6) |
|  | 2 | 27 (23.1) |
|  | 3 | 9 ( 7.7) |
|  | 4 | 3 ( 2.6) |
|  | 5 | 13 (11.1) |

# Descriptive graphs ratings

Here is an example of scatterplot for the MATech definition ratings. Should look just like the PDF report of the last day extraction.

# Analysis ExpertLens

Descriptives rating variables, including participants who answered all questions are presented in Figure 1.

Table with the agreement indices for the definition ratings:

Table 3: Summary statistics definition ratings

| Question | Outcome | median | LowerIPR | UpperIPR | IPRCP | AssymIndex | IPRAS | IPR | DisagIndex | Disagreement |
| --- | --- | --- | --- | --- | --- | --- | --- | --- | --- | --- |
| Definition | Agreement | 7.02454 | 6.102282 | 7.685385 | 6.893834 | 1.893834 | 5.190750 | 1.583103 | 0.3049855 | FALSE |
| Definition | Clarity | 7.26393 | 6.734114 | 7.904322 | 7.319218 | 2.319218 | 5.828827 | 1.170208 | 0.2007621 | FALSE |

Table with the agreement indices:

Table 4: Summary statistics descriptor group ratings

| Question | Outcome | median | LowerIPR | UpperIPR | IPRCP | AssymIndex | IPRAS | IPR | DisagIndex | Disagreement |
| --- | --- | --- | --- | --- | --- | --- | --- | --- | --- | --- |
| Implementation strategies | Relevance | 7.545454 | 7.053557 | 7.948266 | 7.500912 | 2.5009116 | 6.101367 | 0.8947099 | 0.1466409 | FALSE |
| Implementation strategies | Clarity | 7.583851 | 7.185429 | 8.021858 | 7.603643 | 2.6036434 | 6.255465 | 0.8364297 | 0.1337118 | FALSE |
| Implementation outcomes | Relevance | 7.583621 | 7.107131 | 7.975716 | 7.541423 | 2.5414235 | 6.162135 | 0.8685848 | 0.1409552 | FALSE |
| Implementation outcomes | Clarity | 7.667678 | 7.202857 | 8.057721 | 7.630289 | 2.6302894 | 6.295434 | 0.8548639 | 0.1357911 | FALSE |
| Use-related quality | Relevance | 7.490798 | 7.005607 | 8.015636 | 7.510622 | 2.5106216 | 6.115932 | 1.0100282 | 0.1651470 | FALSE |
| Use-related quality | Clarity | 7.654719 | 7.122954 | 8.148044 | 7.635499 | 2.6354990 | 6.303248 | 1.0250894 | 0.1626288 | FALSE |
| Policy-related quality | Relevance | 7.231106 | 6.905521 | 7.669473 | 7.287497 | 2.2874972 | 5.781246 | 0.7639515 | 0.1321431 | FALSE |
| Policy-related quality | Clarity | 7.328446 | 6.754119 | 7.739414 | 7.246767 | 2.2467667 | 5.720150 | 0.9852947 | 0.1722498 | FALSE |
| Research-related quality | Relevance | 7.404908 | 6.993135 | 7.615951 | 7.304543 | 2.3045430 | 5.806815 | 0.6228157 | 0.1072560 | FALSE |
| Research-related quality | Clarity | 7.416422 | 6.914212 | 7.836470 | 7.375341 | 2.3753411 | 5.913012 | 0.9222588 | 0.1559711 | FALSE |
| Technological standards | Relevance | 7.251663 | 6.909006 | 7.535783 | 7.222394 | 2.2223944 | 5.683592 | 0.6267775 | 0.1102784 | FALSE |
| Technological standards | Clarity | 7.538811 | 7.064480 | 7.819704 | 7.442092 | 2.4420921 | 6.013138 | 0.7552244 | 0.1255957 | FALSE |
| Development standards | Relevance | 7.049080 | 6.558282 | 7.425629 | 6.991956 | 1.9919557 | 5.337934 | 0.8673471 | 0.1624874 | FALSE |
| Development standards | Clarity | 7.170088 | 6.821510 | 7.639296 | 7.230403 | 2.2304032 | 5.695605 | 0.8177859 | 0.1435819 | FALSE |
| Scientific evaluation | Relevance | 7.196966 | 6.950118 | 7.858979 | 7.404548 | 2.4045481 | 5.956822 | 0.9088610 | 0.1525748 | FALSE |
| Scientific evaluation | Clarity | 7.515643 | 7.008132 | 7.957498 | 7.482815 | 2.4828150 | 6.074223 | 0.9493652 | 0.1562941 | FALSE |
| ISO certification | Relevance | 6.338491 | 5.502355 | 7.242945 | 6.372650 | 1.3726499 | 4.408975 | 1.7405897 | 0.3947833 | FALSE |
| ISO certification | Clarity | 7.456592 | 6.158582 | 8.026393 | 7.092487 | 2.0924872 | 5.488731 | 1.8678115 | 0.3402993 | FALSE |
| Intervention setting | Relevance | 7.252632 | 6.748837 | 7.645309 | 7.197073 | 2.1970731 | 5.645610 | 0.8964717 | 0.1587910 | FALSE |
| Intervention setting | Clarity | 7.174216 | 6.482927 | 7.672065 | 7.077496 | 2.0774958 | 5.466244 | 1.1891379 | 0.2175421 | FALSE |
| Intervention provider | Relevance | 7.353303 | 7.003241 | 7.858896 | 7.431069 | 2.4310686 | 5.996603 | 0.8556542 | 0.1426898 | FALSE |
| Intervention provider | Clarity | 6.995526 | 6.243402 | 7.568915 | 6.906158 | 1.9061584 | 5.209238 | 1.3255132 | 0.2544544 | FALSE |
| Behaviour change techniques | Relevance | 7.490798 | 7.122467 | 7.907976 | 7.515221 | 2.5152211 | 6.122832 | 0.7855087 | 0.1282917 | FALSE |
| Behaviour change techniques | Clarity | 6.931973 | 5.860909 | 7.402368 | 6.631638 | 1.6316383 | 4.797458 | 1.5414584 | 0.3213074 | FALSE |
| Target behaviour determinants | Relevance | 7.463158 | 6.792619 | 7.760736 | 7.276677 | 2.2766774 | 5.765016 | 0.9681176 | 0.1679297 | FALSE |
| Target behaviour determinants | Clarity | 6.611222 | 5.968105 | 7.185567 | 6.576836 | 1.5768360 | 4.715254 | 1.2174619 | 0.2581965 | FALSE |
| Intervention mode of delivery | Relevance | 7.388501 | 6.715207 | 7.955240 | 7.335224 | 2.3352236 | 5.852835 | 1.2400327 | 0.2118687 | FALSE |
| Intervention mode of delivery | Clarity | 6.919091 | 6.110217 | 7.517646 | 6.813932 | 1.8139318 | 5.070898 | 1.4074288 | 0.2775502 | FALSE |
| Medication adherence intervention | Relevance | 7.019672 | 6.499386 | 7.622817 | 7.061102 | 2.0611019 | 5.441653 | 1.1234308 | 0.2064503 | FALSE |
| Medication adherence intervention | Clarity | 5.665474 | 4.656626 | 6.609384 | 5.633005 | 0.6330049 | 3.299507 | 1.9527586 | 0.5918334 | FALSE |
| Medication adherence measurement | Relevance | 7.364341 | 6.986551 | 7.761847 | 7.374199 | 2.3741990 | 5.911298 | 0.7752958 | 0.1311549 | FALSE |
| Medication adherence measurement | Clarity | 6.369021 | 4.795562 | 6.973418 | 5.884490 | 0.8844902 | 3.676735 | 2.1778563 | 0.5923343 | FALSE |
| Medication adherence phase | Relevance | 7.435165 | 6.925126 | 7.853220 | 7.389173 | 2.3891730 | 5.933759 | 0.9280943 | 0.1564091 | FALSE |
| Medication adherence phase | Clarity | 6.070938 | 4.864469 | 7.165396 | 6.014932 | 1.0149323 | 3.872399 | 2.3009271 | 0.5941866 | FALSE |
| Medication regimen | Relevance | 7.338218 | 7.016583 | 7.758168 | 7.387375 | 2.3873753 | 5.931063 | 0.7415847 | 0.1250340 | FALSE |
| Medication regimen | Clarity | 6.938988 | 6.101845 | 7.557963 | 6.829904 | 1.8299038 | 5.094856 | 1.4561174 | 0.2858015 | FALSE |
| Target health conditions | Relevance | 7.106610 | 6.435583 | 7.972973 | 7.204278 | 2.2042779 | 5.656417 | 1.5373902 | 0.2717958 | FALSE |
| Target health conditions | Clarity | 7.009274 | 6.346405 | 7.782609 | 7.064507 | 2.0645070 | 5.446760 | 1.4362035 | 0.2636803 | FALSE |
| Target use scenario | Relevance | 7.662577 | 6.990857 | 8.006135 | 7.498496 | 2.4984959 | 6.097744 | 1.0152781 | 0.1665006 | FALSE |
| Target use scenario | Clarity | 6.975000 | 6.068392 | 7.560208 | 6.814300 | 1.8143003 | 5.071451 | 1.4918161 | 0.2941597 | FALSE |
| Product and provider information | Relevance | 7.038379 | 6.550489 | 7.701422 | 7.125955 | 2.1259552 | 5.538933 | 1.1509332 | 0.2077897 | FALSE |
| Product and provider information | Clarity | 7.029325 | 6.478261 | 7.580645 | 7.029453 | 2.0294530 | 5.394179 | 1.1023843 | 0.2043655 | FALSE |

## Subgroup analyses

### Descriptives stratified by stakeholder group.

#### Healthcare practitioners

Table with the agreement indices for the definition ratings:

Table 5: Summary statistics definition ratings HCP

| Question | Outcome | median | LowerIPR | UpperIPR | IPRCP | AssymIndex | IPRAS | IPR | DisagIndex | Disagreement |
| --- | --- | --- | --- | --- | --- | --- | --- | --- | --- | --- |
| Definition | Agreement | 7.005873 | 6.107313 | 7.448124 | 6.777718 | 1.777718 | 5.016578 | 1.340811 | 0.2672761 | FALSE |
| Definition | Clarity | 7.382274 | 6.512543 | 7.857585 | 7.185064 | 2.185064 | 5.627597 | 1.345042 | 0.2390082 | FALSE |

Table with the agreement indices:

Table 6: Summary statistics descriptor group ratings HCP

| Question | Outcome | median | LowerIPR | UpperIPR | IPRCP | AssymIndex | IPRAS | IPR | DisagIndex | Disagreement |
| --- | --- | --- | --- | --- | --- | --- | --- | --- | --- | --- |
| Implementation strategies | Relevance | 7.520801 | 7.026032 | 7.921428 | 7.473730 | 2.4737302 | 6.060595 | 0.8953960 | 0.1477406 | FALSE |
| Implementation strategies | Clarity | 7.544622 | 7.052840 | 7.989787 | 7.521314 | 2.5213138 | 6.131971 | 0.9369469 | 0.1527970 | FALSE |
| Implementation outcomes | Relevance | 7.473650 | 7.095842 | 7.787462 | 7.441652 | 2.4416518 | 6.012478 | 0.6916196 | 0.1150307 | FALSE |
| Implementation outcomes | Clarity | 7.561316 | 7.041011 | 7.939207 | 7.490109 | 2.4901090 | 6.085164 | 0.8981966 | 0.1476043 | FALSE |
| Use-related quality | Relevance | 7.312830 | 6.979567 | 7.971323 | 7.475445 | 2.4754452 | 6.063168 | 0.9917560 | 0.1635706 | FALSE |
| Use-related quality | Clarity | 7.615836 | 7.045455 | 8.014230 | 7.529843 | 2.5298426 | 6.144764 | 0.9687752 | 0.1576586 | FALSE |
| Policy-related quality | Relevance | 7.209086 | 6.910537 | 7.486918 | 7.198727 | 2.1987274 | 5.648091 | 0.5763804 | 0.1020487 | FALSE |
| Policy-related quality | Clarity | 7.230767 | 6.718857 | 7.604565 | 7.161711 | 2.1617107 | 5.592566 | 0.8857077 | 0.1583723 | FALSE |
| Research-related quality | Relevance | 7.311301 | 6.987024 | 7.597198 | 7.292111 | 2.2921109 | 5.788166 | 0.6101738 | 0.1054175 | FALSE |
| Research-related quality | Clarity | 7.369502 | 6.923290 | 7.730435 | 7.326862 | 2.3268621 | 5.840293 | 0.8071453 | 0.1382029 | FALSE |
| Technological standards | Relevance | 7.137184 | 6.802390 | 7.506516 | 7.154453 | 2.1544534 | 5.581680 | 0.7041260 | 0.1261495 | FALSE |
| Technological standards | Clarity | 7.260870 | 6.994135 | 7.666978 | 7.330556 | 2.3305563 | 5.845834 | 0.6728429 | 0.1150978 | FALSE |
| Development standards | Relevance | 7.037113 | 6.548466 | 7.261054 | 6.904760 | 1.9047603 | 5.207141 | 0.7125881 | 0.1368483 | FALSE |
| Development standards | Clarity | 7.107209 | 6.838454 | 7.571261 | 7.204857 | 2.2048574 | 5.657286 | 0.7328071 | 0.1295333 | FALSE |
| Scientific evaluation | Relevance | 7.089542 | 6.930410 | 7.744648 | 7.337529 | 2.3375289 | 5.856293 | 0.8142373 | 0.1390363 | FALSE |
| Scientific evaluation | Clarity | 7.517356 | 7.012667 | 8.000293 | 7.506480 | 2.5064802 | 6.109720 | 0.9876262 | 0.1616484 | FALSE |
| ISO certification | Relevance | 6.338491 | 5.915421 | 7.307544 | 6.611483 | 1.6114827 | 4.767224 | 1.3921232 | 0.2920197 | FALSE |
| ISO certification | Clarity | 6.876833 | 5.986492 | 7.888906 | 6.937699 | 1.9376987 | 5.256548 | 1.9024143 | 0.3619132 | FALSE |
| Intervention setting | Relevance | 7.082192 | 6.600968 | 7.535791 | 7.068379 | 2.0683794 | 5.452569 | 0.9348228 | 0.1714463 | FALSE |
| Intervention setting | Clarity | 7.031042 | 6.365054 | 7.590029 | 6.977542 | 1.9775419 | 5.316313 | 1.2249749 | 0.2304181 | FALSE |
| Intervention provider | Relevance | 7.311168 | 7.000463 | 7.792638 | 7.396551 | 2.3965507 | 5.944826 | 0.7921747 | 0.1332545 | FALSE |
| Intervention provider | Clarity | 6.786521 | 6.001645 | 7.419050 | 6.710348 | 1.7103479 | 4.915522 | 1.4174051 | 0.2883529 | FALSE |
| Behaviour change techniques | Relevance | 7.588957 | 7.157862 | 7.972390 | 7.565126 | 2.5651259 | 6.197689 | 0.8145285 | 0.1314245 | FALSE |
| Behaviour change techniques | Clarity | 6.931973 | 5.979381 | 7.308504 | 6.643943 | 1.6439429 | 4.815914 | 1.3291230 | 0.2759856 | FALSE |
| Target behaviour determinants | Relevance | 7.196319 | 6.506773 | 7.829448 | 7.168111 | 2.1681105 | 5.602166 | 1.3226748 | 0.2361006 | FALSE |
| Target behaviour determinants | Clarity | 6.639752 | 5.896849 | 7.375091 | 6.635970 | 1.6359703 | 4.803955 | 1.4782418 | 0.3077135 | FALSE |
| Intervention mode of delivery | Relevance | 7.454976 | 6.890742 | 7.980799 | 7.435771 | 2.4357710 | 6.003656 | 1.0900572 | 0.1815655 | FALSE |
| Intervention mode of delivery | Clarity | 6.937888 | 6.030839 | 7.545157 | 6.787998 | 1.7879982 | 5.031997 | 1.5143183 | 0.3009378 | FALSE |
| Medication adherence intervention | Relevance | 6.977310 | 6.334584 | 7.783983 | 7.059284 | 2.0592836 | 5.438925 | 1.4493984 | 0.2664862 | FALSE |
| Medication adherence intervention | Clarity | 5.997067 | 4.738869 | 6.584321 | 5.661595 | 0.6615948 | 3.342392 | 1.8454522 | 0.5521351 | FALSE |
| Medication adherence measurement | Relevance | 7.268027 | 6.990953 | 7.779263 | 7.385108 | 2.3851078 | 5.927662 | 0.7883103 | 0.1329884 | FALSE |
| Medication adherence measurement | Clarity | 6.514347 | 5.218182 | 7.201643 | 6.209913 | 1.2099126 | 4.164869 | 1.9834616 | 0.4762363 | FALSE |
| Medication adherence phase | Relevance | 7.380368 | 6.987878 | 7.990353 | 7.489115 | 2.4891152 | 6.083673 | 1.0024750 | 0.1647812 | FALSE |
| Medication adherence phase | Clarity | 5.585567 | 4.819355 | 7.046087 | 5.932721 | 0.9327210 | 3.749082 | 2.2267324 | 0.5939408 | FALSE |
| Medication regimen | Relevance | 7.356848 | 6.841230 | 7.989423 | 7.415326 | 2.4153265 | 5.972990 | 1.1481937 | 0.1922310 | FALSE |
| Medication regimen | Clarity | 6.982405 | 6.466289 | 7.986231 | 7.226260 | 2.2262597 | 5.689389 | 1.5199420 | 0.2671538 | FALSE |
| Target health conditions | Relevance | 7.106610 | 6.422128 | 7.992894 | 7.207511 | 2.2075111 | 5.661267 | 1.5707662 | 0.2774584 | FALSE |
| Target health conditions | Clarity | 7.058957 | 6.226117 | 7.928004 | 7.077060 | 2.0770602 | 5.465590 | 1.7018874 | 0.3113822 | FALSE |
| Target use scenario | Relevance | 7.748466 | 6.956302 | 8.003681 | 7.479991 | 2.4799914 | 6.069987 | 1.0473792 | 0.1725505 | FALSE |
| Target use scenario | Clarity | 6.991266 | 6.230316 | 7.885271 | 7.057793 | 2.0577935 | 5.436690 | 1.6549542 | 0.3044047 | FALSE |
| Product and provider information | Relevance | 7.014598 | 6.215423 | 7.702622 | 6.959023 | 1.9590228 | 5.288534 | 1.4871988 | 0.2812119 | FALSE |
| Product and provider information | Clarity | 6.987578 | 6.064429 | 7.379487 | 6.721958 | 1.7219580 | 4.932937 | 1.3150583 | 0.2665873 | FALSE |

#### Policy and decision makers

Table with the agreement indices for the definition ratings:

Table 7: Summary statistics definition ratings PDM

| Question | Outcome | median | LowerIPR | UpperIPR | IPRCP | AssymIndex | IPRAS | IPR | DisagIndex | Disagreement |
| --- | --- | --- | --- | --- | --- | --- | --- | --- | --- | --- |
| Definition | Agreement | 6.860140 | 6.189029 | 7.404741 | 6.796885 | 1.796885 | 5.045328 | 1.2157120 | 0.240958 | FALSE |
| Definition | Clarity | 7.545454 | 7.139064 | 7.974709 | 7.556887 | 2.556887 | 6.185330 | 0.8356445 | 0.135101 | FALSE |

Table with the agreement indices:

Table 8: Summary statistics descriptor group ratings PDM

| Question | Outcome | median | LowerIPR | UpperIPR | IPRCP | AssymIndex | IPRAS | IPR | DisagIndex | Disagreement |
| --- | --- | --- | --- | --- | --- | --- | --- | --- | --- | --- |
| Implementation strategies | Relevance | 7.758294 | 7.208321 | 8.142774 | 7.675548 | 2.6755475 | 6.363321 | 0.9344540 | 0.1468500 | FALSE |
| Implementation strategies | Clarity | 7.703976 | 7.302884 | 8.097093 | 7.699988 | 2.6999885 | 6.399983 | 0.7942100 | 0.1240957 | FALSE |
| Implementation outcomes | Relevance | 7.634016 | 7.385933 | 7.997054 | 7.691493 | 2.6914935 | 6.387240 | 0.6111207 | 0.0956784 | FALSE |
| Implementation outcomes | Clarity | 7.750733 | 7.568276 | 8.047077 | 7.807677 | 2.8076765 | 6.561515 | 0.4788013 | 0.0729712 | FALSE |
| Use-related quality | Relevance | 7.749141 | 6.894888 | 7.995765 | 7.445326 | 2.4453261 | 6.017989 | 1.1008772 | 0.1829311 | FALSE |
| Use-related quality | Clarity | 7.986768 | 7.138199 | 8.361415 | 7.749807 | 2.7498072 | 6.474711 | 1.2232161 | 0.1889221 | FALSE |
| Policy-related quality | Relevance | 7.392638 | 6.735108 | 7.903870 | 7.319489 | 2.3194892 | 5.829234 | 1.1687616 | 0.2005000 | FALSE |
| Policy-related quality | Clarity | 7.322581 | 6.840869 | 7.716716 | 7.278792 | 2.2787925 | 5.768189 | 0.8758461 | 0.1518408 | FALSE |
| Research-related quality | Relevance | 7.404908 | 7.045252 | 7.732940 | 7.389096 | 2.3890961 | 5.933644 | 0.6876875 | 0.1158963 | FALSE |
| Research-related quality | Clarity | 7.252199 | 6.306668 | 7.659864 | 6.983266 | 1.9832659 | 5.324899 | 1.3531965 | 0.2541262 | FALSE |
| Technological standards | Relevance | 7.398104 | 6.736590 | 7.610108 | 7.173349 | 2.1733488 | 5.610023 | 0.8735186 | 0.1557068 | FALSE |
| Technological standards | Clarity | 7.627566 | 7.335616 | 7.973813 | 7.654714 | 2.6547143 | 6.332071 | 0.6381972 | 0.1007881 | FALSE |
| Development standards | Relevance | 7.096473 | 6.076270 | 7.384377 | 6.730324 | 1.7303237 | 4.945486 | 1.3081063 | 0.2645051 | FALSE |
| Development standards | Clarity | 7.138648 | 6.989761 | 7.653951 | 7.321856 | 2.3218559 | 5.832784 | 0.6641905 | 0.1138720 | FALSE |
| Scientific evaluation | Relevance | 7.257669 | 6.668468 | 7.723815 | 7.196142 | 2.1961419 | 5.644213 | 1.0553469 | 0.1869786 | FALSE |
| Scientific evaluation | Clarity | 7.456554 | 6.038923 | 7.773021 | 6.905972 | 1.9059719 | 5.208958 | 1.7340973 | 0.3329068 | FALSE |
| ISO certification | Relevance | 6.325153 | 5.929273 | 7.575951 | 6.752612 | 1.7526121 | 4.978918 | 1.6466776 | 0.3307300 | FALSE |
| ISO certification | Clarity | 8.017013 | 6.252786 | 8.130866 | 7.191826 | 2.1918257 | 5.637739 | 1.8780796 | 0.3331264 | FALSE |
| Intervention setting | Relevance | 7.122699 | 6.334883 | 7.554590 | 6.944737 | 1.9447366 | 5.267105 | 1.2197074 | 0.2315708 | FALSE |
| Intervention setting | Clarity | 6.853372 | 6.273693 | 7.652481 | 6.963087 | 1.9630870 | 5.294630 | 1.3787879 | 0.2604125 | FALSE |
| Intervention provider | Relevance | 7.147239 | 6.772994 | 7.348475 | 7.060734 | 2.0607340 | 5.441101 | 0.5754810 | 0.1057655 | FALSE |
| Intervention provider | Clarity | 6.360825 | 5.291379 | 6.790507 | 6.040943 | 1.0409430 | 3.911415 | 1.4991282 | 0.3832701 | FALSE |
| Behaviour change techniques | Relevance | 7.476954 | 6.980515 | 7.974233 | 7.477374 | 2.4773741 | 6.066061 | 0.9937182 | 0.1638160 | FALSE |
| Behaviour change techniques | Clarity | 6.897210 | 5.737757 | 7.400086 | 6.568922 | 1.5689217 | 4.703383 | 1.6623285 | 0.3534325 | FALSE |
| Target behaviour determinants | Relevance | 7.501718 | 6.528184 | 7.900614 | 7.214399 | 2.2143988 | 5.671598 | 1.3724294 | 0.2419828 | FALSE |
| Target behaviour determinants | Clarity | 6.595308 | 5.544595 | 7.422987 | 6.483791 | 1.4837909 | 4.575686 | 1.8783922 | 0.4105159 | FALSE |
| Intervention mode of delivery | Relevance | 7.404908 | 7.080982 | 8.136412 | 7.608697 | 2.6086969 | 6.263045 | 1.0554306 | 0.1685172 | FALSE |
| Intervention mode of delivery | Clarity | 6.724340 | 5.253372 | 7.167801 | 6.210587 | 1.2105866 | 4.165880 | 1.9144282 | 0.4595496 | FALSE |
| Medication adherence intervention | Relevance | 6.766871 | 6.487117 | 7.328823 | 6.907970 | 1.9079696 | 5.211955 | 0.8417062 | 0.1614953 | FALSE |
| Medication adherence intervention | Clarity | 5.665474 | 4.564490 | 6.356012 | 5.460251 | 0.4602508 | 3.040376 | 1.7915218 | 0.5892434 | FALSE |
| Medication adherence measurement | Relevance | 7.515337 | 7.030148 | 7.927014 | 7.478581 | 2.4785810 | 6.067871 | 0.8968664 | 0.1478058 | FALSE |
| Medication adherence measurement | Clarity | 6.513197 | 4.690323 | 7.028469 | 5.859396 | 0.8593958 | 3.639094 | 2.3381465 | 0.6425079 | FALSE |
| Medication adherence phase | Relevance | 7.453988 | 6.931037 | 7.938389 | 7.434713 | 2.4347130 | 6.002070 | 1.0073513 | 0.1678340 | FALSE |
| Medication adherence phase | Clarity | 6.070938 | 5.154839 | 6.907331 | 6.031085 | 1.0310850 | 3.896628 | 1.7524927 | 0.4497460 | FALSE |
| Medication regimen | Relevance | 7.625767 | 6.886666 | 8.176702 | 7.531684 | 2.5316842 | 6.147526 | 1.2900358 | 0.2098463 | FALSE |
| Medication regimen | Clarity | 7.017595 | 6.499780 | 7.660076 | 7.079928 | 2.0799276 | 5.469891 | 1.1602960 | 0.2121241 | FALSE |
| Target health conditions | Relevance | 7.355828 | 6.122038 | 7.991353 | 7.056695 | 2.0566954 | 5.435043 | 1.8693150 | 0.3439375 | FALSE |
| Target health conditions | Clarity | 7.782609 | 6.937798 | 7.997615 | 7.467707 | 2.4677066 | 6.051560 | 1.0598171 | 0.1751312 | FALSE |
| Target use scenario | Relevance | 7.578199 | 7.000884 | 8.010985 | 7.505934 | 2.5059343 | 6.108901 | 1.0101009 | 0.1653490 | FALSE |
| Target use scenario | Clarity | 6.988658 | 6.482698 | 7.678099 | 7.080399 | 2.0803985 | 5.470598 | 1.1954012 | 0.2185138 | FALSE |
| Product and provider information | Relevance | 7.147750 | 6.373528 | 7.642671 | 7.008100 | 2.0080996 | 5.362149 | 1.2691437 | 0.2366856 | FALSE |
| Product and provider information | Clarity | 7.328446 | 6.717434 | 7.973151 | 7.345292 | 2.3452924 | 5.867939 | 1.2557167 | 0.2139962 | FALSE |

#### Patient representatives

Table with the agreement indices for the definition ratings:

Table 9: Summary statistics definition ratings PRP

| Question | Outcome | median | LowerIPR | UpperIPR | IPRCP | AssymIndex | IPRAS | IPR | DisagIndex | Disagreement |
| --- | --- | --- | --- | --- | --- | --- | --- | --- | --- | --- |
| Definition | Agreement | 6.742813 | 5.994091 | 7.197503 | 6.595797 | 1.595797 | 4.743696 | 1.203412 | 0.2536865 | FALSE |
| Definition | Clarity | 7.093795 | 6.627809 | 7.742994 | 7.185402 | 2.185402 | 5.628103 | 1.115185 | 0.1981457 | FALSE |

Table with the agreement indices:

Table 10: Summary statistics descriptor group ratings PRP

| Question | Outcome | median | LowerIPR | UpperIPR | IPRCP | AssymIndex | IPRAS | IPR | DisagIndex | Disagreement |
| --- | --- | --- | --- | --- | --- | --- | --- | --- | --- | --- |
| Implementation strategies | Relevance | 7.748514 | 7.509423 | 7.939877 | 7.724650 | 2.7246500 | 6.436975 | 0.4304545 | 0.0668722 | FALSE |
| Implementation strategies | Clarity | 7.641187 | 7.524027 | 7.982054 | 7.753041 | 2.7530409 | 6.479561 | 0.4580268 | 0.0706879 | FALSE |
| Implementation outcomes | Relevance | 7.714286 | 7.091172 | 7.935402 | 7.513287 | 2.5132869 | 6.119930 | 0.8442300 | 0.1379477 | FALSE |
| Implementation outcomes | Clarity | 7.718535 | 7.042979 | 8.066081 | 7.554530 | 2.5545300 | 6.181795 | 1.0231016 | 0.1655023 | FALSE |
| Use-related quality | Relevance | 7.537085 | 6.845465 | 7.968496 | 7.406981 | 2.4069807 | 5.960471 | 1.1230311 | 0.1884132 | FALSE |
| Use-related quality | Clarity | 7.761676 | 6.973149 | 8.379807 | 7.676478 | 2.6764781 | 6.364717 | 1.4066575 | 0.2210086 | FALSE |
| Policy-related quality | Relevance | 7.161136 | 6.714208 | 7.755910 | 7.235059 | 2.2350591 | 5.702589 | 1.0417011 | 0.1826716 | FALSE |
| Policy-related quality | Clarity | 7.363636 | 6.730367 | 7.592391 | 7.161379 | 2.1613790 | 5.592068 | 0.8620234 | 0.1541511 | FALSE |
| Research-related quality | Relevance | 7.018234 | 6.747351 | 7.631548 | 7.189449 | 2.1894494 | 5.634174 | 0.8841968 | 0.1569346 | FALSE |
| Research-related quality | Clarity | 7.278258 | 6.201831 | 7.951396 | 7.076614 | 2.0766136 | 5.464920 | 1.7495642 | 0.3201445 | FALSE |
| Technological standards | Relevance | 6.878359 | 6.676302 | 7.339505 | 7.007904 | 2.0079037 | 5.361855 | 0.6632036 | 0.1236892 | FALSE |
| Technological standards | Clarity | 7.559154 | 7.020532 | 7.900788 | 7.460660 | 2.4606603 | 6.040990 | 0.8802559 | 0.1457138 | FALSE |
| Development standards | Relevance | 6.533742 | 5.851978 | 7.011561 | 6.431770 | 1.4317699 | 4.497655 | 1.1595829 | 0.2578195 | FALSE |
| Development standards | Clarity | 7.064272 | 7.029325 | 7.567011 | 7.298168 | 2.2981681 | 5.797252 | 0.5376851 | 0.0927483 | FALSE |
| Scientific evaluation | Relevance | 7.001647 | 6.522872 | 7.385250 | 6.954061 | 1.9540614 | 5.281092 | 0.8623779 | 0.1632954 | FALSE |
| Scientific evaluation | Clarity | 7.398627 | 6.657359 | 7.804692 | 7.231026 | 2.2310257 | 5.696539 | 1.1473327 | 0.2014087 | FALSE |
| ISO certification | Relevance | 6.195547 | 5.887804 | 7.004332 | 6.446068 | 1.4460676 | 4.519101 | 1.1165278 | 0.2470685 | FALSE |
| ISO certification | Clarity | 6.545777 | 6.014651 | 8.025455 | 7.020053 | 2.0200527 | 5.380079 | 2.0108045 | 0.3737500 | FALSE |
| Intervention setting | Relevance | 6.672570 | 6.323246 | 7.184298 | 6.753772 | 1.7537719 | 4.980658 | 0.8610521 | 0.1728792 | FALSE |
| Intervention setting | Clarity | 6.883534 | 6.263134 | 7.491551 | 6.877343 | 1.8773427 | 5.166014 | 1.2284173 | 0.2377882 | FALSE |
| Intervention provider | Relevance | 7.203532 | 6.578341 | 7.520013 | 7.049177 | 2.0491771 | 5.423766 | 0.9416714 | 0.1736195 | FALSE |
| Intervention provider | Clarity | 6.437011 | 5.017934 | 6.992311 | 6.005123 | 1.0051228 | 3.857684 | 1.9743769 | 0.5118037 | FALSE |
| Behaviour change techniques | Relevance | 7.471606 | 6.947696 | 7.834300 | 7.390998 | 2.3909979 | 5.936497 | 0.8866042 | 0.1493480 | FALSE |
| Behaviour change techniques | Clarity | 6.941771 | 5.684871 | 7.332775 | 6.508823 | 1.5088226 | 4.613234 | 1.6479041 | 0.3572123 | FALSE |
| Target behaviour determinants | Relevance | 7.082084 | 6.496068 | 7.656392 | 7.076230 | 2.0762301 | 5.464345 | 1.1603249 | 0.2123447 | FALSE |
| Target behaviour determinants | Clarity | 6.143695 | 5.303812 | 7.435659 | 6.369736 | 1.3697356 | 4.404603 | 2.1318465 | 0.4840042 | FALSE |
| Intervention mode of delivery | Relevance | 7.396794 | 6.880793 | 7.988059 | 7.434426 | 2.4344261 | 6.001639 | 1.1072665 | 0.1844940 | FALSE |
| Intervention mode of delivery | Clarity | 6.548387 | 5.726049 | 7.398777 | 6.562413 | 1.5624129 | 4.693619 | 1.6727275 | 0.3563833 | FALSE |
| Medication adherence intervention | Relevance | 6.756234 | 6.348914 | 7.040546 | 6.694730 | 1.6947298 | 4.892095 | 0.6916315 | 0.1413774 | FALSE |
| Medication adherence intervention | Clarity | 5.257877 | 4.517902 | 6.185251 | 5.351577 | 0.3515767 | 2.877365 | 1.6673494 | 0.5794710 | FALSE |
| Medication adherence measurement | Relevance | 7.363256 | 6.935484 | 7.647853 | 7.291668 | 2.2916684 | 5.787503 | 0.7123687 | 0.1230874 | FALSE |
| Medication adherence measurement | Clarity | 6.571847 | 4.552722 | 7.310126 | 5.931424 | 0.9314239 | 3.747136 | 2.7574035 | 0.7358696 | FALSE |
| Medication adherence phase | Relevance | 7.034314 | 6.538650 | 7.756721 | 7.147686 | 2.1476859 | 5.571529 | 1.2180711 | 0.2186242 | FALSE |
| Medication adherence phase | Clarity | 5.480913 | 4.456764 | 7.278006 | 5.867385 | 0.8673852 | 3.651078 | 2.8212414 | 0.7727147 | FALSE |
| Medication regimen | Relevance | 7.070297 | 6.622021 | 7.436810 | 7.029415 | 2.0294154 | 5.394123 | 0.8147888 | 0.1510512 | FALSE |
| Medication regimen | Clarity | 6.974179 | 5.288341 | 7.492748 | 6.390544 | 1.3905445 | 4.435817 | 2.2044075 | 0.4969564 | FALSE |
| Target health conditions | Relevance | 6.864876 | 5.291599 | 6.998012 | 6.144806 | 1.1448056 | 4.067208 | 1.7064126 | 0.4195538 | FALSE |
| Target health conditions | Clarity | 7.550196 | 6.063715 | 7.988517 | 7.026116 | 2.0261162 | 5.389174 | 1.9248023 | 0.3571609 | FALSE |
| Target use scenario | Relevance | 7.154194 | 6.548524 | 7.598924 | 7.073724 | 2.0737239 | 5.460586 | 1.0504006 | 0.1923604 | FALSE |
| Target use scenario | Clarity | 6.875515 | 6.093255 | 7.717951 | 6.905603 | 1.9056033 | 5.208405 | 1.6246964 | 0.3119374 | FALSE |
| Product and provider information | Relevance | 6.948038 | 5.950920 | 7.387488 | 6.669204 | 1.6692042 | 4.853806 | 1.4365679 | 0.2959673 | FALSE |
| Product and provider information | Clarity | 6.637986 | 6.040131 | 7.059298 | 6.549714 | 1.5497142 | 4.674571 | 1.0191670 | 0.2180236 | FALSE |

#### Health IT specialists

Table with the agreement indices for the definition ratings:

Table 11: Summary statistics definition ratings HIT

| Question | Outcome | median | LowerIPR | UpperIPR | IPRCP | AssymIndex | IPRAS | IPR | DisagIndex | Disagreement |
| --- | --- | --- | --- | --- | --- | --- | --- | --- | --- | --- |
| Definition | Agreement | 6.744023 | 6.013883 | 7.412166 | 6.713025 | 1.713025 | 4.919537 | 1.398284 | 0.2842307 | FALSE |
| Definition | Clarity | 7.598220 | 7.008109 | 8.011144 | 7.509626 | 2.509626 | 6.114439 | 1.003035 | 0.1640436 | FALSE |

Table with the agreement indices:

Table 12: Summary statistics descriptor group ratings HIT

| Question | Outcome | median | LowerIPR | UpperIPR | IPRCP | AssymIndex | IPRAS | IPR | DisagIndex | Disagreement |
| --- | --- | --- | --- | --- | --- | --- | --- | --- | --- | --- |
| Implementation strategies | Relevance | 7.581551 | 7.092220 | 8.016296 | 7.554258 | 2.5542584 | 6.181388 | 0.9240763 | 0.1494933 | FALSE |
| Implementation strategies | Clarity | 7.561926 | 7.218299 | 7.968362 | 7.593330 | 2.5933304 | 6.239996 | 0.7500640 | 0.1202026 | FALSE |
| Implementation outcomes | Relevance | 7.714286 | 7.031110 | 7.894751 | 7.462931 | 2.4629309 | 6.044396 | 0.8636411 | 0.1428829 | FALSE |
| Implementation outcomes | Clarity | 7.756598 | 7.413381 | 8.077538 | 7.745460 | 2.7454596 | 6.468189 | 0.6641563 | 0.1026804 | FALSE |
| Use-related quality | Relevance | 7.312830 | 6.656116 | 7.989916 | 7.323016 | 2.3230157 | 5.834524 | 1.3338001 | 0.2286048 | FALSE |
| Use-related quality | Clarity | 7.517529 | 7.000421 | 8.188948 | 7.594684 | 2.5946841 | 6.242026 | 1.1885268 | 0.1904072 | FALSE |
| Policy-related quality | Relevance | 7.183460 | 6.672063 | 7.512883 | 7.092473 | 2.0924731 | 5.488710 | 0.8408207 | 0.1531910 | FALSE |
| Policy-related quality | Clarity | 7.363636 | 6.723549 | 7.632258 | 7.177904 | 2.1779035 | 5.616855 | 0.9087091 | 0.1617825 | FALSE |
| Research-related quality | Relevance | 7.184336 | 6.777504 | 7.653854 | 7.215679 | 2.2156789 | 5.673518 | 0.8763501 | 0.1544633 | FALSE |
| Research-related quality | Clarity | 7.432774 | 6.242971 | 7.968106 | 7.105538 | 2.1055385 | 5.508308 | 1.7251341 | 0.3131877 | FALSE |
| Technological standards | Relevance | 7.137184 | 6.755569 | 7.526183 | 7.140876 | 2.1408762 | 5.561314 | 0.7706140 | 0.1385669 | FALSE |
| Technological standards | Clarity | 7.574374 | 6.731067 | 7.952227 | 7.341647 | 2.3416472 | 5.862471 | 1.2211596 | 0.2083012 | FALSE |
| Development standards | Relevance | 6.696429 | 5.980403 | 7.110926 | 6.545664 | 1.5456643 | 4.668496 | 1.1305235 | 0.2421601 | FALSE |
| Development standards | Clarity | 7.170088 | 6.951015 | 7.573352 | 7.262183 | 2.2621833 | 5.743275 | 0.6223370 | 0.1083592 | FALSE |
| Scientific evaluation | Relevance | 7.041954 | 6.439529 | 7.783481 | 7.111505 | 2.1115048 | 5.517257 | 1.3439525 | 0.2435907 | FALSE |
| Scientific evaluation | Clarity | 7.311883 | 6.027007 | 7.956661 | 6.991834 | 1.9918339 | 5.337751 | 1.9296546 | 0.3615108 | FALSE |
| ISO certification | Relevance | 6.321345 | 5.601783 | 7.261350 | 6.431566 | 1.4315665 | 4.497350 | 1.6595664 | 0.3690099 | FALSE |
| ISO certification | Clarity | 7.208946 | 6.431085 | 8.211794 | 7.321440 | 2.3214398 | 5.832160 | 1.7807095 | 0.3053259 | FALSE |
| Intervention setting | Relevance | 6.324324 | 5.805387 | 6.975944 | 6.390665 | 1.3906655 | 4.435998 | 1.1705563 | 0.2638766 | FALSE |
| Intervention setting | Clarity | 6.557587 | 5.664954 | 7.396918 | 6.530936 | 1.5309359 | 4.646404 | 1.7319635 | 0.3727535 | FALSE |
| Intervention provider | Relevance | 7.020731 | 6.420139 | 7.307109 | 6.863624 | 1.8636240 | 5.145436 | 0.8869699 | 0.1723799 | FALSE |
| Intervention provider | Clarity | 6.478006 | 5.714941 | 7.002806 | 6.358873 | 1.3588734 | 4.388310 | 1.2878644 | 0.2934762 | FALSE |
| Behaviour change techniques | Relevance | 7.206493 | 6.973967 | 7.780066 | 7.377016 | 2.3770162 | 5.915524 | 0.8060990 | 0.1362684 | FALSE |
| Behaviour change techniques | Clarity | 6.696522 | 5.263442 | 7.458776 | 6.361109 | 1.3611088 | 4.391663 | 2.1953339 | 0.4998867 | FALSE |
| Target behaviour determinants | Relevance | 6.963190 | 6.342223 | 7.584049 | 6.963136 | 1.9631362 | 5.294704 | 1.2418258 | 0.2345411 | FALSE |
| Target behaviour determinants | Clarity | 6.607717 | 5.499707 | 7.373720 | 6.436713 | 1.4367135 | 4.505070 | 1.8740134 | 0.4159787 | FALSE |
| Intervention mode of delivery | Relevance | 7.085890 | 6.337442 | 7.479621 | 6.908531 | 1.9085315 | 5.212797 | 1.1421786 | 0.2191105 | FALSE |
| Intervention mode of delivery | Clarity | 6.937348 | 4.562463 | 7.449267 | 6.005865 | 1.0058651 | 3.858798 | 2.8868035 | 0.7481096 | FALSE |
| Medication adherence intervention | Relevance | 6.602390 | 6.185274 | 6.831002 | 6.508138 | 1.5081375 | 4.612206 | 0.6457281 | 0.1400042 | FALSE |
| Medication adherence intervention | Clarity | 5.657680 | 4.280682 | 6.291496 | 5.286089 | 0.2860887 | 2.779133 | 2.0108137 | 0.7235399 | FALSE |
| Medication adherence measurement | Relevance | 7.515337 | 7.059748 | 7.974081 | 7.516915 | 2.5169145 | 6.125372 | 0.9143337 | 0.1492699 | FALSE |
| Medication adherence measurement | Clarity | 6.630498 | 5.544282 | 7.167241 | 6.355761 | 1.3557612 | 4.383642 | 1.6229594 | 0.3702308 | FALSE |
| Medication adherence phase | Relevance | 7.233138 | 6.577914 | 7.749887 | 7.163901 | 2.1639007 | 5.595851 | 1.1719732 | 0.2094361 | FALSE |
| Medication adherence phase | Clarity | 5.512492 | 4.323197 | 7.355932 | 5.839564 | 0.8395645 | 3.609347 | 3.0327351 | 0.8402449 | FALSE |
| Medication regimen | Relevance | 7.048467 | 6.410496 | 7.894595 | 7.152545 | 2.1525452 | 5.578818 | 1.4840988 | 0.2660239 | FALSE |
| Medication regimen | Clarity | 7.024767 | 5.980991 | 7.570839 | 6.775915 | 1.7759151 | 5.013873 | 1.5898488 | 0.3170900 | FALSE |
| Target health conditions | Relevance | 6.569143 | 5.112861 | 6.975914 | 6.044387 | 1.0443872 | 3.916581 | 1.8630535 | 0.4756837 | FALSE |
| Target health conditions | Clarity | 7.550196 | 6.627250 | 7.980136 | 7.303693 | 2.3036925 | 5.805539 | 1.3528859 | 0.2330337 | FALSE |
| Target use scenario | Relevance | 7.247308 | 6.512929 | 7.988451 | 7.250690 | 2.2506899 | 5.726035 | 1.4755228 | 0.2576867 | FALSE |
| Target use scenario | Clarity | 6.898600 | 6.393548 | 7.514496 | 6.954022 | 1.9540223 | 5.281033 | 1.1209478 | 0.2122592 | FALSE |
| Product and provider information | Relevance | 6.765048 | 5.976679 | 7.309970 | 6.643324 | 1.6433244 | 4.814987 | 1.3332912 | 0.2769044 | FALSE |
| Product and provider information | Clarity | 6.735532 | 6.015939 | 7.388810 | 6.702375 | 1.7023749 | 4.903562 | 1.3728713 | 0.2799743 | FALSE |

#### Research and education

Table with the agreement indices for the definition ratings:

Table 13: Summary statistics definition ratings REP

| Question | Outcome | median | LowerIPR | UpperIPR | IPRCP | AssymIndex | IPRAS | IPR | DisagIndex | Disagreement |
| --- | --- | --- | --- | --- | --- | --- | --- | --- | --- | --- |
| Definition | Agreement | 7.048083 | 6.099569 | 7.773204 | 6.936387 | 1.936387 | 5.254580 | 1.673635 | 0.3185097 | FALSE |
| Definition | Clarity | 7.477962 | 6.909730 | 8.005908 | 7.457819 | 2.457819 | 6.036728 | 1.096178 | 0.1815849 | FALSE |

Table with the agreement indices:

Table 14: Summary statistics descriptor group ratings REP

| Question | Outcome | median | LowerIPR | UpperIPR | IPRCP | AssymIndex | IPRAS | IPR | DisagIndex | Disagreement |
| --- | --- | --- | --- | --- | --- | --- | --- | --- | --- | --- |
| Implementation strategies | Relevance | 7.535885 | 7.060438 | 8.030679 | 7.545558 | 2.5455583 | 6.168337 | 0.9702411 | 0.1572938 | FALSE |
| Implementation strategies | Clarity | 7.624146 | 7.205279 | 8.074329 | 7.639804 | 2.6398036 | 6.309705 | 0.8690500 | 0.1377323 | FALSE |
| Implementation outcomes | Relevance | 7.503068 | 7.077945 | 7.833742 | 7.455844 | 2.4558435 | 6.033765 | 0.7557970 | 0.1252613 | FALSE |
| Implementation outcomes | Clarity | 7.565517 | 7.265144 | 8.040469 | 7.652807 | 2.6528067 | 6.329210 | 0.7753251 | 0.1224995 | FALSE |
| Use-related quality | Relevance | 7.257669 | 6.858973 | 7.952141 | 7.405557 | 2.4055572 | 5.958336 | 1.0931680 | 0.1834687 | FALSE |
| Use-related quality | Clarity | 7.526767 | 6.998755 | 8.042301 | 7.520528 | 2.5205282 | 6.130792 | 1.0435465 | 0.1702140 | FALSE |
| Policy-related quality | Relevance | 7.029941 | 6.710895 | 7.481549 | 7.096222 | 2.0962219 | 5.494333 | 0.7706545 | 0.1402635 | FALSE |
| Policy-related quality | Clarity | 7.328446 | 6.733070 | 7.637435 | 7.185252 | 2.1852523 | 5.627878 | 0.9043653 | 0.1606938 | FALSE |
| Research-related quality | Relevance | 7.455480 | 7.006152 | 7.663804 | 7.334978 | 2.3349779 | 5.852467 | 0.6576516 | 0.1123717 | FALSE |
| Research-related quality | Clarity | 7.432774 | 6.871770 | 7.835620 | 7.353695 | 2.3536951 | 5.880543 | 0.9638494 | 0.1639048 | FALSE |
| Technological standards | Relevance | 7.168421 | 6.770172 | 7.495705 | 7.132939 | 2.1329388 | 5.549408 | 0.7255334 | 0.1307407 | FALSE |
| Technological standards | Clarity | 7.574374 | 7.010557 | 7.914077 | 7.462317 | 2.4623169 | 6.043475 | 0.9035195 | 0.1495033 | FALSE |
| Development standards | Relevance | 6.990755 | 6.076614 | 7.287692 | 6.682153 | 1.6821530 | 4.873229 | 1.2110782 | 0.2485166 | FALSE |
| Development standards | Clarity | 7.107209 | 6.871422 | 7.526031 | 7.198726 | 2.1987261 | 5.648089 | 0.6546087 | 0.1158991 | FALSE |
| Scientific evaluation | Relevance | 7.057786 | 6.751715 | 7.915149 | 7.333432 | 2.3334318 | 5.850148 | 1.1634339 | 0.1988726 | FALSE |
| Scientific evaluation | Clarity | 7.428153 | 6.988270 | 7.907729 | 7.447999 | 2.4479991 | 6.021999 | 0.9194587 | 0.1526833 | FALSE |
| ISO certification | Relevance | 6.321345 | 5.390021 | 7.240491 | 6.315256 | 1.3152561 | 4.322884 | 1.8504693 | 0.4280636 | FALSE |
| ISO certification | Clarity | 7.178886 | 5.992882 | 8.007157 | 7.000019 | 2.0000192 | 5.350029 | 2.0142752 | 0.3764980 | FALSE |
| Intervention setting | Relevance | 7.030552 | 6.334223 | 7.542949 | 6.938586 | 1.9385859 | 5.257879 | 1.2087255 | 0.2298884 | FALSE |
| Intervention setting | Clarity | 7.021945 | 6.465423 | 7.525299 | 6.995361 | 1.9953614 | 5.343042 | 1.0598758 | 0.1983656 | FALSE |
| Intervention provider | Relevance | 7.181229 | 6.952237 | 7.538521 | 7.245379 | 2.2453791 | 5.718069 | 0.5862841 | 0.1025318 | FALSE |
| Intervention provider | Clarity | 6.706745 | 5.771566 | 7.063099 | 6.417332 | 1.4173321 | 4.475998 | 1.2915334 | 0.2885464 | FALSE |
| Behaviour change techniques | Relevance | 7.358306 | 7.003776 | 7.855552 | 7.429664 | 2.4296642 | 5.994496 | 0.8517756 | 0.1420929 | FALSE |
| Behaviour change techniques | Clarity | 6.835920 | 5.443249 | 7.317889 | 6.380569 | 1.3805687 | 4.420853 | 1.8746398 | 0.4240448 | FALSE |
| Target behaviour determinants | Relevance | 7.463158 | 6.706925 | 7.661726 | 7.184325 | 2.1843254 | 5.626488 | 0.9548010 | 0.1696975 | FALSE |
| Target behaviour determinants | Clarity | 6.607717 | 5.998240 | 7.039540 | 6.518890 | 1.5188904 | 4.628336 | 1.0412998 | 0.2249836 | FALSE |
| Intervention mode of delivery | Relevance | 7.085890 | 6.376166 | 7.608589 | 6.992377 | 1.9923774 | 5.338566 | 1.2324231 | 0.2308528 | FALSE |
| Intervention mode of delivery | Clarity | 6.670533 | 5.286956 | 7.383779 | 6.335368 | 1.3353676 | 4.353051 | 2.0968221 | 0.4816902 | FALSE |
| Medication adherence intervention | Relevance | 6.756234 | 6.353510 | 7.363741 | 6.858625 | 1.8586255 | 5.137938 | 1.0102317 | 0.1966220 | FALSE |
| Medication adherence intervention | Clarity | 5.629928 | 4.442356 | 6.445093 | 5.443724 | 0.4437243 | 3.015586 | 2.0027368 | 0.6641285 | FALSE |
| Medication adherence measurement | Relevance | 7.397394 | 6.986645 | 7.716952 | 7.351798 | 2.3517983 | 5.877698 | 0.7303071 | 0.1242506 | FALSE |
| Medication adherence measurement | Clarity | 6.513197 | 5.002346 | 7.015822 | 6.009084 | 1.0090838 | 3.863626 | 2.0134754 | 0.5211363 | FALSE |
| Medication adherence phase | Relevance | 7.490078 | 6.838078 | 7.772906 | 7.305492 | 2.3054920 | 5.808238 | 0.9348284 | 0.1609487 | FALSE |
| Medication adherence phase | Clarity | 6.259756 | 4.948257 | 7.377966 | 6.163111 | 1.1631112 | 4.094667 | 2.4297095 | 0.5933839 | FALSE |
| Medication regimen | Relevance | 7.386278 | 6.945812 | 7.916974 | 7.431393 | 2.4313932 | 5.997090 | 0.9711618 | 0.1619388 | FALSE |
| Medication regimen | Clarity | 6.939742 | 6.048808 | 7.572320 | 6.810564 | 1.8105641 | 5.065846 | 1.5235125 | 0.3007420 | FALSE |
| Target health conditions | Relevance | 6.980119 | 6.402130 | 7.692951 | 7.047541 | 2.0475405 | 5.421311 | 1.2908204 | 0.2381012 | FALSE |
| Target health conditions | Clarity | 6.995717 | 6.354389 | 7.809231 | 7.081810 | 2.0818099 | 5.472715 | 1.4548424 | 0.2658356 | FALSE |
| Target use scenario | Relevance | 7.527687 | 6.677570 | 7.993865 | 7.335717 | 2.3357173 | 5.853576 | 1.3162954 | 0.2248703 | FALSE |
| Target use scenario | Clarity | 6.989962 | 6.434204 | 7.595512 | 7.014858 | 2.0148577 | 5.372287 | 1.1613084 | 0.2161665 | FALSE |
| Product and provider information | Relevance | 6.963190 | 6.348562 | 7.655366 | 7.001964 | 2.0019638 | 5.352946 | 1.3068041 | 0.2441280 | FALSE |
| Product and provider information | Clarity | 6.934066 | 6.057615 | 7.499432 | 6.778524 | 1.7785237 | 5.017786 | 1.4418167 | 0.2873412 | FALSE |

### Reliability of ratings per group

The reliability of ratings per question across stakeholder groups was tested via intraclass correlation coefficient (ICC) based on two-way random model, considering type (average measures) and definition of relationship (consistency); median rating per subgroup taken as one rater.

Table 15: Median descriptor groups ratings for all subgroups

| Question | Outcome | REP.median | HCP.median | PDM.median | PRP.median | HIT.median |
| --- | --- | --- | --- | --- | --- | --- |
| Implementation strategies | Relevance | 7.535885 | 7.520801 | 7.758294 | 7.748514 | 7.581551 |
| Implementation strategies | Clarity | 7.624146 | 7.544622 | 7.703976 | 7.641187 | 7.561926 |
| Implementation outcomes | Relevance | 7.503068 | 7.473650 | 7.634016 | 7.714286 | 7.714286 |
| Implementation outcomes | Clarity | 7.565517 | 7.561316 | 7.750733 | 7.718535 | 7.756598 |
| Use-related quality | Relevance | 7.257669 | 7.312830 | 7.749141 | 7.537085 | 7.312830 |
| Use-related quality | Clarity | 7.526767 | 7.615836 | 7.986768 | 7.761676 | 7.517529 |
| Policy-related quality | Relevance | 7.029941 | 7.209086 | 7.392638 | 7.161136 | 7.183460 |
| Policy-related quality | Clarity | 7.328446 | 7.230767 | 7.322581 | 7.363636 | 7.363636 |
| Research-related quality | Relevance | 7.455480 | 7.311301 | 7.404908 | 7.018234 | 7.184336 |
| Research-related quality | Clarity | 7.432774 | 7.369502 | 7.252199 | 7.278258 | 7.432774 |
| Technological standards | Relevance | 7.168421 | 7.137184 | 7.398104 | 6.878359 | 7.137184 |
| Technological standards | Clarity | 7.574374 | 7.260870 | 7.627566 | 7.559154 | 7.574374 |
| Development standards | Relevance | 6.990755 | 7.037113 | 7.096473 | 6.533742 | 6.696429 |
| Development standards | Clarity | 7.107209 | 7.107209 | 7.138648 | 7.064272 | 7.170088 |
| Scientific evaluation | Relevance | 7.057786 | 7.089542 | 7.257669 | 7.001647 | 7.041954 |
| Scientific evaluation | Clarity | 7.428153 | 7.517356 | 7.456554 | 7.398627 | 7.311883 |
| ISO certification | Relevance | 6.321345 | 6.338491 | 6.325153 | 6.195547 | 6.321345 |
| ISO certification | Clarity | 7.178886 | 6.876833 | 8.017013 | 6.545777 | 7.208946 |
| Intervention setting | Relevance | 7.030552 | 7.082192 | 7.122699 | 6.672570 | 6.324324 |
| Intervention setting | Clarity | 7.021945 | 7.031042 | 6.853372 | 6.883534 | 6.557587 |
| Intervention provider | Relevance | 7.181229 | 7.311168 | 7.147239 | 7.203532 | 7.020731 |
| Intervention provider | Clarity | 6.706745 | 6.786521 | 6.360825 | 6.437011 | 6.478006 |
| Behaviour change techniques | Relevance | 7.358306 | 7.588957 | 7.476954 | 7.471606 | 7.206493 |
| Behaviour change techniques | Clarity | 6.835920 | 6.931973 | 6.897210 | 6.941771 | 6.696522 |
| Target behaviour determinants | Relevance | 7.463158 | 7.196319 | 7.501718 | 7.082084 | 6.963190 |
| Target behaviour determinants | Clarity | 6.607717 | 6.639752 | 6.595308 | 6.143695 | 6.607717 |
| Intervention mode of delivery | Relevance | 7.085890 | 7.454976 | 7.404908 | 7.396794 | 7.085890 |
| Intervention mode of delivery | Clarity | 6.670533 | 6.937888 | 6.724340 | 6.548387 | 6.937348 |
| Medication adherence intervention | Relevance | 6.756234 | 6.977310 | 6.766871 | 6.756234 | 6.602390 |
| Medication adherence intervention | Clarity | 5.629928 | 5.997067 | 5.665474 | 5.257877 | 5.657680 |
| Medication adherence measurement | Relevance | 7.397394 | 7.268027 | 7.515337 | 7.363256 | 7.515337 |
| Medication adherence measurement | Clarity | 6.513197 | 6.514347 | 6.513197 | 6.571847 | 6.630498 |
| Medication adherence phase | Relevance | 7.490078 | 7.380368 | 7.453988 | 7.034314 | 7.233138 |
| Medication adherence phase | Clarity | 6.259756 | 5.585567 | 6.070938 | 5.480913 | 5.512492 |
| Medication regimen | Relevance | 7.386278 | 7.356848 | 7.625767 | 7.070297 | 7.048467 |
| Medication regimen | Clarity | 6.939742 | 6.982405 | 7.017595 | 6.974179 | 7.024767 |
| Target health conditions | Relevance | 6.980119 | 7.106610 | 7.355828 | 6.864876 | 6.569143 |
| Target health conditions | Clarity | 6.995717 | 7.058957 | 7.782609 | 7.550196 | 7.550196 |
| Target use scenario | Relevance | 7.527687 | 7.748466 | 7.578199 | 7.154194 | 7.247308 |
| Target use scenario | Clarity | 6.989962 | 6.991266 | 6.988658 | 6.875515 | 6.898600 |
| Product and provider information | Relevance | 6.963190 | 7.014598 | 7.147750 | 6.948038 | 6.765048 |
| Product and provider information | Clarity | 6.934066 | 6.987578 | 7.328446 | 6.637986 | 6.735532 |

Across items (relevance and clarity included), the ICC value across the median ratings of the 5 subgroups was 0.97, indicating good reliability. For Relevance, the ICC was 0.95 and for Clarity it was 0.97. These results show that, if we consider items as ‘subjects of rating’ and median value of groups as individual raters, inter-rater reliability is above the pre-established threshold of good reliability, meaning that stakeholder groups rated the relevance and clarity of the descriptor groups in similar ways.

```
  hcp  healthIT   patient    policy    res.ed
```

18.857143 12.571429 18.857143 8.380952 28.285714

DefinitionAgreement : Checking singularity: TRUE [1] NA

DefinitionClarity : Checking singularity: TRUE [1] NA

D1.1.Product.and.provider.information.Relevance : Checking singularity: FALSE # Intraclass Correlation Coefficient

```
 Adjusted ICC: 0.029
```

Conditional ICC: 0.029

D1.1.Product.and.provider.information.Clarity : Checking singularity: TRUE [1] NA

D2.1.Target.use.scenario.Relevance : Checking singularity: FALSE # Intraclass Correlation Coefficient

```
 Adjusted ICC: 0.037
```

Conditional ICC: 0.037

D2.1.Target.use.scenario.Clarity : Checking singularity: TRUE [1] NA

D2.2.Target.health.conditions.Relevance : Checking singularity: FALSE # Intraclass Correlation Coefficient

```
 Adjusted ICC: 0.191
```

Conditional ICC: 0.191

D2.2.Target.health.conditions.Clarity : Checking singularity: TRUE [1] NA

D2.3..Medication.regimen.Relevance : Checking singularity: TRUE [1] NA

D2.3..Medication.regimen.Clarity : Checking singularity: TRUE [1] NA

D2.4.1..Phase.of.medication.adherence.Relevance : Checking singularity: TRUE [1] NA

D2.4.1..Phase.of.medication.adherence.Clarity : Checking singularity: TRUE [1] NA

D2.4.2.A.Monitoring.measurement.type.of.management.Relevance : Checking singularity: TRUE [1] NA

D2.4.2.A.Monitoring.measurement.type.of.management.Clarity : Checking singularity: TRUE [1] NA

D2.4.2.B.Support.intervention.type.of.management.Relevance : Checking singularity: FALSE # Intraclass Correlation Coefficient

```
 Adjusted ICC: 0.046
```

Conditional ICC: 0.046

D2.4.2.B.Support.intervention.type.of.management.Clarity : Checking singularity: FALSE # Intraclass Correlation Coefficient

```
 Adjusted ICC: 0.009
```

Conditional ICC: 0.009

D2.4.2.B.1.Intervention.modes.of.delivery.Relevance : Checking singularity: FALSE # Intraclass Correlation Coefficient

```
 Adjusted ICC: 0.112
```

Conditional ICC: 0.112

D2.4.2.B.1.Intervention.modes.of.delivery.Clarity : Checking singularity: TRUE [1] NA

D2.4.2.B.2.Target.behaviour.determinants..Relevance : Checking singularity: TRUE [1] NA

D2.4.2.B.2.Target.behaviour.determinants..Clarity : Checking singularity: TRUE [1] NA

D2.4.2.B.3.Behaviour.change.techniques.Relevance : Checking singularity: TRUE [1] NA

D2.4.2.B.3.Behaviour.change.techniques.Clarity : Checking singularity: FALSE # Intraclass Correlation Coefficient

```
 Adjusted ICC: 0.025
```

Conditional ICC: 0.025

D2.4.2.B.4.Intervention.provider.Relevance : Checking singularity: FALSE # Intraclass Correlation Coefficient

```
 Adjusted ICC: 0.098
```

Conditional ICC: 0.098

D2.4.2.B.4.Intervention.provider.Clarity : Checking singularity: FALSE # Intraclass Correlation Coefficient

```
 Adjusted ICC: 0.111
```

Conditional ICC: 0.111

D2.4.2.B.5.Intervention.setting..Relevance : Checking singularity: FALSE # Intraclass Correlation Coefficient

```
 Adjusted ICC: 0.205
```

Conditional ICC: 0.205

D2.4.2.B.5.Intervention.setting..Clarity : Checking singularity: FALSE # Intraclass Correlation Coefficient

```
 Adjusted ICC: 0.069
```

Conditional ICC: 0.069

D3.1.1.A.ISO.certification.Relevance : Checking singularity: TRUE [1] NA

D3.1.1.A.ISO.certification.Clarity : Checking singularity: TRUE [1] NA

D3.1.1.B.Evidence.from.scientific.evaluation.Relevance : Checking singularity: FALSE # Intraclass Correlation Coefficient

```
 Adjusted ICC: 0.085
```

Conditional ICC: 0.085

D3.1.1.B.Evidence.from.scientific.evaluation.Clarity : Checking singularity: FALSE # Intraclass Correlation Coefficient

```
 Adjusted ICC: 0.044
```

Conditional ICC: 0.044

D3.1.1.C.Development.standards.Relevance : Checking singularity: FALSE # Intraclass Correlation Coefficient

```
 Adjusted ICC: 0.050
```

Conditional ICC: 0.050

D3.1.1.C.Development.standards.Clarity : Checking singularity: TRUE [1] NA

D3.1.1.D.Technological.standards.Relevance : Checking singularity: FALSE # Intraclass Correlation Coefficient

```
 Adjusted ICC: 0.039
```

Conditional ICC: 0.039

D3.1.1.D.Technological.standards.Clarity : Checking singularity: TRUE [1] NA

D3.1.2.Research.related.quality.indicators.Relevance : Checking singularity: FALSE # Intraclass Correlation Coefficient

```
 Adjusted ICC: 0.023
```

Conditional ICC: 0.023

D3.1.2.Research.related.quality.indicators.Clarity : Checking singularity: TRUE [1] NA

D3.1.3.Policy.related.quality.indicators.Relevance : Checking singularity: TRUE [1] NA

D3.1.3.Policy.related.quality.indicators.Clarity : Checking singularity: TRUE [1] NA

D3.1.4.Use.related.quality.indicators.Relevance : Checking singularity: TRUE [1] NA

D3.1.4.Use.related.quality.indicators.Clarity : Checking singularity: FALSE # Intraclass Correlation Coefficient

```
 Adjusted ICC: 0.006
```

Conditional ICC: 0.006

D3.2.1.Implementation.outcomes.Relevance : Checking singularity: TRUE [1] NA

D3.2.1.Implementation.outcomes.Clarity : Checking singularity: TRUE [1] NA

To examine whether there are any differences specific to each item, 2-level models were performed for each item, considering respondents nested within stakeholder group. Since 34 of 83 respondents indicated more than 1 group, it is necessary to allocate one single group for each of of the 34 respondents. One option is to maximize the equal distribution of participants in groups by allocating those with >2 groups to the one least represented. This decision resulted in 27 respondents in the research and education group, 18 in the HCP group, 12 in the healthIT group, 8 in the policy group, and 18 in the patient group.

The proportion of variance explained by the second level (the stakeholder group) was for most items either 0 (model almost/near singular) or below 0.1, with only two items with ICC above 0.15, i.e. 0.19 (target health conditions relevance) and 0.205 (intervention setting relevance). This indicates that >/= 80 percent of variance is within groups and therefore the differences across groups do not justify further investigation per item.

# Temporal analysis of response rates and stability (analysis of process indicators)

This analysis examined how stakeholders’ responses evolved through iterations and if and how consensus or certain level of agreement has formed.

Stability of response refers to the consistency of responses within the study period and to between respondent group stability, and has been assessed by the coefficient of quartile variation (CQV).

We have explored visually the evolution during the 78 survey days with two types of visualisations: for each question, the evolution of each respondent ratings color-coded by stakeholder group, and for each stakeholder group the evolution of median values of ratings.

CQV was calculated with data from the last day of survey over all participants (CQVtotal) and within each stakeholder group (CQVsub) to account for expected higher variation in response between different stakeholder groups.

A CQVtotal less than 30% and CQVsub less than 15% was considered as stable response.

The visualisation of individual ratings shows that most respondents did not change their ratings throughout the Delphi survey, and stakeholder groups had similar distribution of ratings across the 1 to 9 scale.

The visualisation of the evolution across the survey period of the median responses for each item per stakeholder group shows that most items converge towards the last day on median ratings between 5 and 8 on a scale from 1 to 9. Therefore, across time we can see a stabilisation of the median values in the last half of the RT Delphi period.

Table 16: CQV values at closing of survey

| item | CQVtotal | hcp | healthIT | patient | policy | res.ed |
| --- | --- | --- | --- | --- | --- | --- |
| D1.1.Product.and.provider.information.Clarity | 13.65 | 8.68 | 11.43 | 10.03 | 4.06 | 12.53 |
| D1.1.Product.and.provider.information.Relevance | 11.14 | 6.99 | 9.77 | 13.75 | 6.54 | 10.09 |
| D2.1.Target.use.scenario.Clarity | 13.10 | 13.83 | 10.61 | 15.62 | 8.69 | 13.18 |
| D2.1.Target.use.scenario.Relevance | 7.88 | 6.66 | 10.62 | 8.63 | 6.72 | 8.69 |
| D2.2.Target.health.conditions.Clarity | 13.14 | 7.69 | 21.60 | 16.37 | 13.84 | 8.74 |
| D2.2.Target.health.conditions.Relevance | 11.35 | 8.42 | 13.03 | 18.47 | 4.29 | 12.62 |
| D2.3..Medication.regimen.Clarity | 12.77 | 9.02 | 14.91 | 18.72 | 7.95 | 12.75 |
| D2.3..Medication.regimen.Relevance | 7.38 | 6.57 | 11.77 | 11.64 | 3.07 | 6.32 |
| D2.4.1..Phase.of.medication.adherence.Clarity | 25.03 | 23.47 | 37.23 | 28.13 | 6.84 | 21.49 |
| D2.4.1..Phase.of.medication.adherence.Relevance | 8.47 | 6.68 | 8.16 | 10.64 | 5.44 | 8.07 |
| D2.4.2.A.Monitoring.measurement.type.of.management.Clarity | 21.26 | 12.31 | 14.92 | 29.43 | 18.74 | 20.51 |
| D2.4.2.A.Monitoring.measurement.type.of.management.Relevance | 6.73 | 4.61 | 8.28 | 6.41 | 10.37 | 4.98 |
| D2.4.2.B.1.Intervention.modes.of.delivery.Clarity | 13.12 | 9.10 | 13.34 | 27.30 | 9.39 | 19.95 |
| D2.4.2.B.1.Intervention.modes.of.delivery.Relevance | 11.23 | 5.01 | 19.95 | 9.19 | 9.56 | 9.56 |
| D2.4.2.B.2.Target.behaviour.determinants..Clarity | 12.61 | 11.27 | 14.21 | 19.54 | 11.65 | 7.90 |
| D2.4.2.B.2.Target.behaviour.determinants..Relevance | 10.08 | 7.34 | 8.96 | 10.65 | 8.17 | 6.47 |
| D2.4.2.B.3.Behaviour.change.techniques.Clarity | 14.24 | 6.15 | 20.96 | 14.64 | 11.75 | 14.34 |
| D2.4.2.B.3.Behaviour.change.techniques.Relevance | 7.08 | 2.88 | 3.42 | 7.06 | 8.05 | 6.33 |
| D2.4.2.B.4.Intervention.provider.Clarity | 11.97 | 9.32 | 8.38 | 19.29 | 13.19 | 12.49 |
| D2.4.2.B.4.Intervention.provider.Relevance | 6.71 | 6.72 | 11.32 | 10.05 | 5.29 | 6.04 |
| D2.4.2.B.5.Intervention.setting..Clarity | 10.75 | 7.23 | 14.64 | 14.55 | 6.57 | 8.42 |
| D2.4.2.B.5.Intervention.setting..Relevance | 8.38 | 4.92 | 8.74 | 6.94 | 3.71 | 4.84 |
| D2.4.2.B.Support.intervention.type.of.management.Clarity | 23.72 | 20.55 | 26.34 | 18.61 | 14.68 | 22.19 |
| D2.4.2.B.Support.intervention.type.of.management.Relevance | 10.55 | 7.42 | 7.88 | 8.84 | 7.73 | 8.97 |
| D3.1.1.A.ISO.certification.Clarity | 14.91 | 12.40 | 9.73 | 17.57 | 6.45 | 18.71 |
| D3.1.1.A.ISO.certification.Relevance | 18.84 | 18.55 | 31.10 | 11.64 | 19.64 | 19.62 |
| D3.1.1.B.Evidence.from.scientific.evaluation.Clarity | 7.41 | 7.60 | 14.30 | 12.40 | 12.20 | 6.64 |
| D3.1.1.B.Evidence.from.scientific.evaluation.Relevance | 7.52 | 7.55 | 7.53 | 12.28 | 5.44 | 9.32 |
| D3.1.1.C.Development.standards.Clarity | 7.77 | 8.33 | 8.41 | 4.19 | 8.17 | 9.27 |
| D3.1.1.C.Development.standards.Relevance | 10.99 | 6.67 | 9.74 | 11.23 | 3.03 | 8.89 |
| D3.1.1.D.Technological.standards.Clarity | 6.33 | 3.25 | 12.32 | 6.69 | 2.58 | 6.70 |
| D3.1.1.D.Technological.standards.Relevance | 6.17 | 4.06 | 6.47 | 5.24 | 3.61 | 6.37 |
| D3.1.2.Research.related.quality.indicators.Clarity | 10.02 | 4.35 | 7.54 | 14.35 | 5.02 | 8.59 |
| D3.1.2.Research.related.quality.indicators.Relevance | 5.87 | 5.43 | 5.01 | 8.45 | 3.35 | 6.02 |
| D3.1.3.Policy.related.quality.indicators.Clarity | 8.28 | 4.17 | 8.40 | 9.42 | 8.59 | 8.64 |
| D3.1.3.Policy.related.quality.indicators.Relevance | 7.00 | 5.10 | 5.93 | 9.26 | 6.89 | 6.08 |
| D3.1.4.Use.related.quality.indicators.Clarity | 7.90 | 4.22 | 8.04 | 10.33 | 4.84 | 8.02 |
| D3.1.4.Use.related.quality.indicators.Relevance | 7.61 | 6.86 | 9.40 | 9.19 | 4.44 | 7.25 |
| D3.2.1.Implementation.outcomes.Clarity | 6.97 | 7.66 | 5.35 | 7.45 | 3.87 | 6.54 |
| D3.2.1.Implementation.outcomes.Relevance | 6.41 | 5.31 | 9.54 | 6.28 | 4.66 | 6.46 |
| D3.2.2.Implementation.strategies.Clarity | 6.87 | 6.20 | 7.30 | 3.83 | 6.57 | 7.28 |
| D3.2.2.Implementation.strategies.Relevance | 6.78 | 4.96 | 6.05 | 6.19 | 7.37 | 7.76 |
| DefinitionAgreement | 13.23 | 16.54 | 17.62 | 12.05 | 6.05 | 14.15 |
| DefinitionClarity | 12.74 | 13.76 | 14.50 | 10.43 | 6.58 | 12.01 |

All items showed stability across groups and the great majority also within groups, as per the thresholds established a priori. Some notable exceptions are the higher CQV values for Relevance on ISO Certification (all groups except patients), Intervention modes of delivery (healthIT), and target health conditions (patients). Clarity ratings for several attribute groups have been a matter of controversy within stakeholder groups. These results, together with the qualitative feedback received, would justify the improvement of the definitions and further development of extended explanations on the source of these terms and definitions and their practical applications.
