# Supplementary material for: Stakeholder Consensus on an Interdisciplinary Terminology to Enable the Development and Uptake of Medication Adherence Technologies Across Health Systems: Web-Based Real-Time Delphi Study
Source: J Med Internet Res. 2025 Mar 25;27:e59738. doi: 10.2196/59738 (PMC11979531; doi:10.2196/59738)
Supplement: Multimedia Appendix 5 [file jmir_v27i1e59738_app5.docx]

**ENABLE MATech Repository DELPHI study - Qualitative analysis report.**

Table S1: Coding tree and summary of themes with representative quotes:

| 1. **General feedback survey** | |
| --- | --- |
| Complexity of the format as barrier for participation | The complexity of the presentation, in particular the terminology tree and the 2D format, may represent a barrier to participation, especially for patient representatives.  It is complex and appears to be comprehensive and it appears that all the important and relevant domains or attributes are included - but there is a lot to consider so would be easy to miss something that is obvious.  The graph is comprehensive and all important and relevant domains, groups of attributes or attributes are included  In my opinion, this is too much information to asess as part of a Delphi panel. I cannot land in such a enormous amount of sub-categories and provide any valuable infomration. |
| Technical issues | The links to the support documents were initially not functioning; the technical problem was solved in the first days of the study.  Note that I am not able to open the support documents, as such, my comments may be obviated by what they contain  I could open the document - and the interactive version was it was quite fun! But I fear it is very complicated and I suspect that few responders will take time to explore it properly and give you a meaningful answer. It might be better to leave it to the end of the survey so you don't put people off |
| Text formatting suggestions | Participants commented on the use of parentheses, brackets, abbreviations and proposed improvements.  The text is difficult to understand if part of the information is in parentheses. To improve text comprehensibility, create the information in parentheses as a separate sentence |
| 1. **General feedback repository structure** | |
| Complexity of the structure | Participants commented on the complexity of the structure, which related to the complexity of the presentation; the structure was described as comprehensive.  Although there is too many information in this graph when you open all sub-categories I don't see how to simplify it  Busy and not easy to follow but actually complete.  Comprenhensive but not clear enough.  It is a comprehensive framework, and with previous comments in mind, I think it is complete. However, it is not so easy to get an overview of the graph |
| Repository development | Some participants commented on the use of the attribute framework for the repository, for example mentioning the difference between attributes relevant for research or practice and the need for adapting requirements to the type of technology, anticipating the need for ‘other’ options in the data collection tools or links to other resources, or pointing to challenges regarding the feasibility of use for data collection from developers and maintenance of repository.  Relevance for research world or real world settings/clinical practice? The reflection is different if reference frame is research world or trial world.  One should not expect the same regulation/requirements for a simple reminder app than for an active coaching application  Despite the scheme is too complex for any practical use, at the same time it does not reserve any "other" option to include similar yet not known yet items.  I think there are links to other reference databases missing, for example, which WHO ATC code does the product refer to? Is the product registered as a medical device, if yes, which class etc? Is there an app connected to the product, if yes, link to app store  Please, be realistic about the entering all suggested data. Tools providers may be asked to enter the data about their product and make the product more visible?!  Not clear to me who evaluates these criteria and how you conclude that the categories are fulfilled  A repository is key but its maintenance will be challenging. Given the rapid evolution of technologies, I would use the repository only if its content is up-to-date. The author should thus be responsible for reviewing the content of the entry he/she created every year. In absence of review, the entry should be disabled. |
| Missing attributes | Some participants mentioned attributes they considered missing and necessary, of which some could be mapped on attributes proposed and some were added to the framework (see Justification for changes). Some examples are shown here:  D3.1.4: not only customization of language; could also be a customization of pages of an App as example  I suggest considering adding "and outcomes" to the definition of quality indicators in the Excel document ("Quality indicators are standardized, evidence-based, and measurable items for monitoring and evaluating the quality of healthcare performance AND OUTCOMES")  Consider adaptability in D3.2 Implementation outcomes |
| Patient perspectives | Some comments highlighted the importance of capturing patient perspectives in the framework, such as patient understanding, remembering, preparedness, the meaning attributed to medication, coping strategies, desires and expectations, affordability and inequities of access.  what happens if the patient did not understand what he/she was told by the healthcare provider; what if the patient does not remember? The patient may have misunderstood the healthcare provider; where is this captured  Patient factors are missing eg: fear of side-effects, medication burden  the affordability, the need for ehealth training, and the unintentional non adherence due to inequities in access, cost  As expert Patient EUPATI, I think that the coping strategies, desires, expectations are aspects very important to improve the quality of treatment and therapeutic adherence. I believe to add in the framework this point is ethically correct |
| 1. **MATech definition** | |
| Health technologies | The term ‘technologies’ was interpreted in several different ways by participants. Some considered that it is strictly related to digital tools, some considered it limited to technical solutions, while others supported a broader meaning of this term, i.e. to include analogue tools and services. Variation in meaning across languages was highlighted. Some comments highlighted the need to further clarify the examples of technologies included in the definition. Others referred to the better fit of the attribute framework for describing products, to the detriment of other types of technologies mentioned in the definition.  I agree with another comment that technology first led to the idea that we only speak from technical solutions. This misunderstanding is based on my mother tongue. For me, techniques would be better  I think "procedure" and "system" should be explained  I find the procedure part very confusing: what is a procedure? If it is a broad definition then almost everything is a technology. I would not go that direction. For me the procedure part could be excluded. Or it should be better explained  The term "procedures" is of wide scope, and thus may be subject to several interpretations. It is alluded to include, for example, procedures (and from the explanation we know that they include those "delivered through human interaction")  "devices, procedures or systems developed" not all procedures and systems are technology based, so procedures and systems should focus more on technology  The definition of medication adherence technologies is not fully in line with this framework. In this framework you talk about products, in the definition about devices, procedures or systems  I do agree that the current focus is on "products", and I am not sure how "procedures" and "systems" would fit in. Given that these are very broad categories (which is highly adequate) I am not quite sure whether they can be systematized in a fashion similar to the "products".  I think that the proposed definition is broader than what I understand from the Momerandum of Understanding of COST ENABLE. In my opinion, technology refers to technical solutions and tools (i. e. mobile apps, algorithms etc.) in the MoU  "devices, procedures or systems developed" not all procedures and systems are technology based, so procedures and systems should focus more on technology  All of this sound like you equate "technology" with "electronics" or "information technology". Even though in D1.1 there is an "material" component which captures "product (component) consisting of physical substances or equipment other than electronic", this sounds like a "misc." class to capture all. IMO, we will in the future see a lot of adherence technologies which are based on bio-technology, nano-technology, and other types of technologies |
| Medication adherence | Participants expressed different opinions regarding whether ‘medication adherence’ means taking medicines as prescribed by or as agreed with the healthcare team. Some supported maintaining the ‘prescribed’ term in line with the ABC taxonomy. Among arguments mentioned: agreement is part of the prescription process, thus before the adherence process; agreement, although desirable, is unrealistic and unnecessary for the taking of medication. Others preferred the term ‘agreed’; arguments expressed were: ‘prescribed’ is based on paternalistic model and not patient-centered, and is linked to compliance, while ‘agreed’ suggests patient activation; using the term ‘agreed’ would encourage the community to strive towards agreement as a necessary condition of treatment; ‘agreed’ is used in the WHO definition of adherence; ‘agreed’ captures also situations in which agreement is reached on dose adjustment after prescription. Other comments pointed to exceptions, such as ‘over-the-counter’ medications who may not need prescription. Others highlighted the need for evidence-based prescription as a precondition, since agreement might be reached on a non-evidence-based treatment.  Discussion ensued about focusing only on medication adherence, versus extending the scope to include adherence to other healthcare recommendations.  Some comments pointed to different ways to express the goals of the technologies in relation to adherence, such as enhancing, optimizing, preventing, maintaining, monitoring, and more distal outcomes such as facilitating coping, improving health status, creating a treatment alliance.  "as agreed" should be replaced by "as prescribed".  As defined in the ABC taxonomy, the process of medication adherence starts with an appropriate prescription. There are thus two processes: 1) the process of prescription, and then followed by 2) the process of medication adherence. The notion of agreement is key but is part of the prescription process. Therefore, it should not be included in the definition of "medication adherence technologies"! This principle has been extensively discussed during the elaboration of the ABC taxonomy and should remain aligned to avoid confusion  It is utopian to believe that most prescriptions across the world are "agreed". More specifically, how do you propose to define agreement (need a scale) and what do you do when there is no agreement?  Patients are unlikely to take a prescribed drug if they don't agree with it, so i would see 'agreement' as the first stage in prescribing  If a patient does not agree with what was prescribed, for me their is no common ground for a treatment. I think as an adherence community we should look at models where the patient perspective is included and adherence can only be adherence if a patient agrees on the treatment  It is also possible that the medication might be 'over-the-counter' so that I don't need to prescribe (low dose aspirin is a good example as it is cheaper OTC than on prescription).  I prefer "agreed". To me, this refers more to adherence, whereas "prescribed" refers more to compliance  Also, agreed is used in the WHO definition of adherence  This implies that the patient is an active participant rather than a passive recipient in the process  "As agreed with the health care providers" implies the prerequisite that patient and health care provider both agree with the medication, something which is not always the truth!  "As agreed with the health care providers" implies the prerequisite that patient and health care provider both agree with the medication, something which is not always the truth! On the other hand, "agree" might mean that a health care provider proposed and the patient agrees to take the medicine without this practice covering the evidence based behaviour. I think this should be written as follows: "...patients to take their medications as evidence based prescribed by the healthcare providers".  I would also prefer agreed since a medication can be prescribed with a certain dose, but the doctor and patient might have agreed in advance that the patient can lower the dose considering side effects that the patient might experience. There could also be an agreement between the patient and the doctor that the patient can increase the dose if the disered effect is not reached  it may be pertinent as well to consider adherence to healthcare recommendations that are not strictly about medication adherence. For example, changes in lifestyle  Interesting point, and I would agree. In my experience patients who have problems adhering to lifestyle changes, often have problems adhering to medication as well  Although adherence to changes in lifestyle etc. may overlap medication adherence somehow, I would suggest restricting this repository only on medication adherence. Otherwise there is a risk of the repository broadening too much to be handled  I recommend restricting ENABLE-R to cover only medication adherence tools. Adherence to other HCP advices or lifestyle changes is out of scope, in my opinion  In my opinion the definition should emphasize that these technologies aim to enhance medication adherence / to optimize medication adherence  I would not emphasize only enhancing adherence. A technology may also be provided to a patient when initiating treatment to prevent poor adherence.  definition should also encompass monitoring of adherence  why to support patients only pehaps also to support families, health care providers, I would add to improve the patients health status, pr facilitate theri lives, interactions with health care providers or indicating better ways of copying with the disease |
| Evidence base | The term ‘evidence’ generated debate. Some participants raised questions on the strength of the evidence needed and finding a balance between inclusiveness and evidence standards. Some preferred including technologies under development for which limited, or no, evidence is available. Arguments for this preference were for example encouraging innovation. Others agreed with limiting the repository to technologies that meet some thresholds of evidence. Some comments pointed at the need to decide on concrete rules on the types and amount of evidence necessary.  iI agree that it is a good choice to avoid to be overwhelmed with a lot of tech that have no evidence. We have however to recognize that many technologies are out there  ‘developed based on evidence" might be too restrictive - what about evidence that arises after development  I agree that "developed under evidence" implies that the evidence was available before (or anyway at the time of) development, whcih would exclude enything for which evidence has been compiled only after development. It might be better to separate requirements for development and evidence  evidence (i.e. evidence derived from empirical studies, stakeholder input, theory and contextual information)  empirical evidence alone is not enough. We need to have also include stakeholder input, theory and contextual information to allow to develop solutions for real world settings  What if an unknown party enters the "adherence market" with a new app, which is not based on evidence, but patients love this app and this helps them to take their medication as agreed with the healthcare provider. There is no previous evidence, but I think we can safely say the the app will be a huge success. There is no agreement with the current definition, regarding the evidence  I would also be for keeping the evidence. When (and if) evidence becomes available it should be entered (…)to include every single pill-taking reminder app in one of the app stores would just clutter up the database without any benefit.  Evidence does not have a clear cut off. It sounds ok, however, what it means in practice? This can be a variety of studies/evidence from complete garbage to real quality research. Howe do you distinguish what is "good" evidence from "any" evidence. In a way you can always do something for "any" evidence, which will not be of a standard worth further consideration.  I agree. There are many commercial providers of solutions that argue they are evidence based, but not being it in the proper meaning of evidence based  the word evidence seems strange and unnecessary in this context  If everybody copies evidence based technologies, which ar far from optimal, new techniques or "out of the box" interventions will be neglected  Of course all these criteria are important, but we also need new unvalidated techniques that may improve adherence in the future, even if they are not rigorously tested, nor have a theoretical framework |
| General comments | Some participated expressed agreement to the definition proposed, or provided suggestions for more clarity or precision  The definition is clear yet could reflect more the 'real world setting' where they will be applied  To improve the clarity of the definition it would be better to divide the long sentence in two shorter. This makes it easier to read and understand and improve comprehension  The "detailed explanation" refers to "users" [of the database] and their needs. For this it would be good to describe hwat the target groups are, othrwise it is difficult to cater for their needs  Clear and impactful  The definition is clear |
| 1. **Product and provider information (D1)** | |
| Product | Some participants were concerned about how the product maturity (versioning) would be handled in the repository, and how products which are combination of technologies could be described. Some comments referred to the need to consider the intended use of the product as a first question and adapt the description conditional on the response to this question  Intended use of the product is a key information to start with  product may be a combination of device, procedure or system. this information is missed  First, the "product" may be a combination of items (e.g. a device, an app, and a procedure to use it in care). Therefore, I would change the product definition into: "Product is a device, procedure, system, or a combination thereof, ..." Most of the time, I expect the product to be a combination  When we are talking about "procedures" (or "interventions", which I prefer), I don't think that "name, brand, type, release date," are appropriate  Some indicator of the maturity of the product would help  Maybe "ancestry" of a product would be interesting? E.g., "Product X is an improved version of product Y, which it supersedes" or "Product X is a fusion of products Y and Z".  I would rephrase Product is a branded device, procedure or system, or an app that could be used to facilitate process in order to achieve better adherence to medication. I would delete the described .... as I dont see the value... |
| Provider | Some comments pointed to the difficulty of using the attributes proposed to describe technologies that are developed and distributed by multiple organisations working together, or by groups of persons not forming an organisation.  Provider organization is the organisation that produces and/or makes the product available for users described by its name, type, domain activity, contact details...  There may be providers that are a group working in partnership - how will this be accounted for, particularly when each of these partners might have differing characteristics  Second, I see a potential issue with the label "provider organization", in particular for combination products. For example, in the case of a combination product (e.g. device + system), is the provider organization, the institution that puts the combination in place or the manufacturer of the device? This point should be crystal clear to avoid confusion  i am not sure how partnerships or consortium would be listed among provider organisations. other than that, I see no major elements missing  What if there is no organisation behind the technology and it is just a "private person"? How do you describe the developers of the technology?  The provider type currently caters only for "organisations" of various kinds. Community-driven products (e.g., open source) do not necessarily come from an "organisation". |
| Author and entry | Participants provided suggestions on attribute reformulation and how repository entries would need to be updated by authors.  Author of the product description is a a person or group of persons who enters information about at least one MATech in the ENABLE-R database (ID, name, date)  Given the rapid evolution of technologies, I would use the repository only if its content is up-to-date. The author should thus be responsible for reviewing the content of the entry he/she created every year. In absence of review, the entry should be disabled |
| 1. **Medication adherence descriptors (D2)** | |
| Target use scenarios | Participants pointed to clarifications considered necessary, and potential missing attributes.  "Adherence support use" is not clear for the caregivers: what is his/her role?  In Adherence support use it is nor clear who are professional health and social care provider and health (system) manager  Consider to add category that explicitly refers if development / use was for real world settings  I don't quite understand the term "Person in the healthcare environment (patient or caregiver)". Could it be just "user" or something similar? Because this category is under self-management, I think that the technology is used outside the healthcare environment  "Professional health and social care provider" is very broad "Health (system) manager" should be specified  Patient literacy I miss the eHealth literacy  Patient polypharmacy should be divided in subgroups (count of drugs) |
| Target health conditions | Participants indicated missing attributes and raised questions about the feasibility of using these general categories for very diverse diseases. The ATC and ICD-11 classifications were proposed as alternative sources for these attributes.  Pregnancy can be added separately  a characterisation of chronic conditions vs acute conditions is missing  There should be a way of specifying if the MAT customiseable to diffrent conditions  I am not convinced that this classification is relevant for medication adherence technologies. Most cases will be confronted with multiple diseases. Cardiovascular is a mixed bag of many diseases ranging from benign conditions or preventive medicine to life-threatening ones. Oncology is not on the list as such. From experience, I know that it is extremely hard to make sense of those categories in adherence research as they are more suited to uniquely classify medications. ATC or BNF classifications are good options but they have many levels  Agreed, but many of the interventions created are very disease-specific (even if it would objectively be better to have them specific to the patient as opposed to one of their potentially multiple conditions). As such, I suggest that it's relevant to indicate where that is the case  Too many categories. Overlaps . Many systems have non specific targets  I suggest the possibility to choose multiple conditions for a technology in addition to the "general" category  How has ICD-11 been used? It seems that the conditions are taken directly from HRCS. Personally, I don't like the title "Generic health relevance". If the conditions are "the type of diseases or health problems the technology is intended for", could it be just "Generic"?  Do not understand these categories - why not ATC classification?  type of diseases or health problems: s I struggle as you only mention disease categories but some diseases are responsible for several health problems. But in the description, you mention both  If for medications ATC classification  I wonder if there shouldn't be a specific category for vaccines. While we're not talking about a chronic condition, adherence in vaccine mandates or suggestions is an (increasingly pertinent) topic. Furthermore, in the case of vaccines that require multiple doses, such as most covid vaccines, as well as HPV, drop-off between injections is a serious issue  There would be a better overview and less overlap if the ICD classification is used and the ATC classification for medicines  I miss systems taht can be used for dementia and for addicted. Boxes that only opens at the right time. This factor should be displayed somewhere |
| Medication regimen | Participants commented on several attributes, proposing operational definitions (e.g. cut-off agreed for short and long-term therapy), or additional subcategories (e.g. palliative or symptomatic as type of intention). Some noted inconsistencies with other definitions (as prescribed vs as agreed in the definition of adherence), highlighted the necessity of simple language, and the importance of capturing dose adjustment and as needed use, as well as other recommendations for effective medication intake.  You should define short and long term therapy. What is the cut-off (1 week, 1 month 1 year)?  Number of monitored medications should be revised as all other information are medication specific. I am not fully convinced that we need so detailed information on medication regimen. Most interventions do not focus on a specific medication.  Type of intention as the purpose for which the medication is prescribed (e.g.preventive or therapeutic). Palliative and compassionate use?  I am not convinced that type of intention should be listed under the medication regimen. Sometimes it may be tricky  Medication can also be used according to cyclic courses Preventive, therapeutic, symptomatic  Management of side effects is missed in type of intnetion  Type of intention is missing "prophylactic" as the purpose of prescribing the drug  Here is defined: Medication regimen attributes are the prescribed schematic form/therapeutic plan of medication therapy... But the definition of the intervention (see at the beggining) comprise agreed, which is ok, but does not correspond to D2.3. as well as ABC Taxonomy  The definition uses professional terminology that is difficult to understand for people with poor language skills, seniors, people with a low level of education  or for which self-management action plans enable the patient to adjust their dosage. This needs to be possible within the form  It should be possible to specify if the MAT supports - titration of drugdose. -real time change in the prescription (dosage, frequency etc) - changes made by the patient (for example if the patient takes a diffrent dose) The above is important to get valuable data for the clinical descionmaking, but also for resarch purposes in RealWorldStudies  but there are drugs that are used 'as required'  There are additional recommendations that need to be taken into account, like for example, with food, or without food |
| Medication adherence management | There was debate among participants on the distinction between monitoring and support as elements of adherence management. Some comments recommended following the ABC taxonomy without adaptations. Others questioned whether it would be possible for a monitoring MATech not to intervene and affect the behaviour of patients.  Some participants questioned definitions of measurement, intervention, or measurement targets and noted the need for clearer and simpler definitions.  Inclusion of behaviour change techniques following the Capability-Opportunity-Motivation-Behaviour model was viewed positively by some participants, while others questioned if these models are widely known and simple enough for the use in practice, for example by HCPs  Suggestions were given on clarifying how certain types of MATech could be described according to the proposed attributes (e.g. modes of delivery, provider and setting, etc.), or by expanding the list of attributes or modifying the structure of these attributes.  Not sure if the naming and explanations is ok. "1. Initiation is the phase of adherence that covers the start of a prescribed treatment...." is a bit misleading as it seems they started the treatment. The explanations follows. However, it could be done directly e.g. 1. Initiation is the period from when the prescription is issued to the first dose taken (i.e. the initiation event).  Multiple imprecisions make this statement unclear. It should be a copy/paste of the ABC taxonomy paper.  Implementation should be defined based on the first dose taken or that you say it follows the initiaton. "from the initiation... " is not clear what is meant e.g. from the date of prescribing or from the first day taken or any time in between. Consider another name for "implementation" e.g. treatment, something that describes 2medicines use". Implementation does not sound as the right desciptor.  2.4.1 this sentence is not clear, the meaning is confusing: A medication adherence phase is a time interval between the prescription start and end dates that is behaviorally (i.e. linked with specific determinants and outcomes) and metrically (i.e. requires specific estimation methods) distinct  I agree with this definition in accordance with the ABC taxonomy proposed by Vrijens, but I would improve the definitions of discontinuation. Add specific examples to the definition to better understand the different time period  I am not sure that this is really "management of adherence" (Medication adherence monitoring, or measurement, is type of adherence management that refers to...). I would suppose e.g. the way of evaluating  Not clear, not direct... If you "support" you do not generate change. If you do, than it is an intervention  In the specified context, a list of barriers and enablers of medication adherence need to be linked, at personal level as well as healthcare delivery level. For example, in case of polypharmacy, the complexity may be a barrier. Existence of adverse events may be a barrier. When a multimorbid case is considered, conflicting information or lack of coordination, may be a barrier. Should we not express these relations ?  In the determinants, one should put a positive or negative sign, as the foreseen causal effect on adherence. Indirectly, these are linked to a barrier that we try to decrease, or an enabler that we try to increase  all part 2 is difficult to understand especially determinant measure (casual effects?) shall we use a questionnaire instead? and also difficult in practice to determine behavioral measures without consultation skills  I cannot grasp the convenience of mixing two different important concepts: adherence itself, and its determinants and outcomes. According to this line of reasoning, I would say that "Measurement target" is simply adherence itself. This is not incompatible with the fact that we are also interested in determinants and outcomes of nonadherence, but I would clearly differentiate both areas  I also think that we would need to elaborate more the second part, the measurement target. I doubt that many people behind interventions will be entirely at ease with the sentence "measurement targeting causal influences on the behaviour that can be modifiable"  What we want to change is adherence, and this issue can be addressed both directly to adherence behaviours, and to possible determinants idenfied in any particular patient  do we need a "change" if a patient is 100% adherennt, and we simply want him /her to continue that level of adherence?  Good point to include a behavioural theory COM-B as it will facilitate implementation.  Here you pick the COM-B model, but the most widely used categorization of determinants is the WHO classification in 5 categories. It is much easier for HCPs in general.  Interesting - I find COM-B very intuitive (and I would say more widely used than the WHO classification)  many interventions are explicitly built around ideas coming more from behavioral economics (notably, Kahneman or Thaler) which might be a little more difficult to classify according to the BCT work  many interventions are quite purposefully built around CBT, including concepts from motivational interviewing. These can be viewed through the COM-B framework, notably with respect to motivation, but it would be nice to be able to quickly identify those interventions that use these approaches. For example, a number of mental health programs in the United States have received Class II authorisation largely because they are explicitly built on CBT, which has solid evidence for impact in depression  A 2D organisation would be more clear, having the problems / barriers we want to tackle in X, and the methods for doing so in Y  I would say that COM-B is widely known and understood (including by byHCPs) - but BCTs is where it gets complicated. I can cope with COM-B, but if I need to think about BCTs I ask a Health Psychologist  I'm struggling to understand where smart pill dispensers fit in this scheme. Would they be a "Smart box"? A "Digital event record system"?  The notion of pill count needs to be extended to other delivery routes: weighting of returned cream in dermatology studies, mechanical/digital dose counter on some inhalers (without time stamping).  you cannot conflate these two domains. 1 above is reasonably clear although includes attributes of no validity ( pill count, self declared) but is incmpelte because admin technique is critical for some medications: topical, inhaled, injectable  A lot of electronic devices that are purpose-built for helping with medication adherence are not wearable, and would not fit in this sctucture  I don't think smart boxes etc. as wearable devices. Wearable devices refer to smart watches etc. In addition, there are also smart devices for blister packs.  Pill dispensers (filled by a patient or a caregiver) are another example of an intervention not fitting in these three groups  Electronic Mode needs more subcategories. An App with no interaction only used as a reminder should be categorised at a lower level then a tecnology with feedback possibility with the health care provider |
| 1. **Evaluation and implementation (D3)** | |
| Quality indicators | The need for an evidence base, especially scientific evidence, was supported by some comments and challenged by others. Some considered evaluation conditional on the intended use of the technology. Some highlighted the variation in quality standards across countries and types of technologies.  Some overlaps were noted in the proposed attributes.  Several comments referred to the lesser relevance of the ISO certification.  Suggestions were provided for various attributes, for modification, addition, or exclusion.  Very important facts for potential users.  Very important with user and use related quality indicators  Important but difficult to understand from the patient's point of view and probably also not relevant  Not very clear and relevant information for users/patients in this context. However important, so need through reconsideration on what and how to be explained.  "...standards established in the development of health technologies". Do these exist for all types of MATech? Will all companies be willing to share information on "activities related to preparation, development and optimization of product components as well as the manufacturing, validation and distribution process of the MATech"?  Also here including a contextual perspective might strengthen your criteria -- real world settings are not same as trial world settings  no point in measuring theoretical factors if this is to be a practical tool: surely this will be part of the certification process?  It is clear but should this be part of the repository? This information is required to get reimbursement in the different countries and level of requirement will vary across countries  There is an overlap between D3.1.1->Development Standards->User-centred process->Usability tests and D3.1.4->Usability. Even if the former is concerned with process quality and the latter with product quality, it will be hard to distinguish the two for the purposes of the database. Consider to put them together in one branch of the tree  can we see as attributes here the reproducibility in different patient groups / or otherwise the potential bias  Does some attributes of the previous "Use-related Qis" overlap with acceptability?  Why ISO? MDR has high relevance for digital solutions  Why must we use ISO standards? Others can serve the same purpose  ISO = quality certification (processes, good practice) CE certification is different (accreditation, safety of a medical device) What is needed here, ISO or CE? This point needs certainly more elaboration  Quality, security and efficacy are three aspect of research of new solutions in healthcare. ISo certification is not enough  Why ISO? ISO is of interest if the company factor in its ISO quality management system elements from regulatory frameworks? What about other geulatory framework? MDR, ICH  Certification is of value. However, why should we only/primarily think on ISO only? What about the CE mark, and the others?  ECO is not commonly used terminology. So health economic evaluation or cost-effectiveness indicators is much more widely used in the literature and public sources. Same for CUR, probably regulatory status is more useful.  Cost-effectiveness analyses may be performed independent of reimbursement decisions as well. I.e. not every CE study leads to a decision. There could also be "coverage with evidence development" as a decision  I suggest removing "vs no intervention" and "vs other interventions" regarding cost-effectiveness because if this sub-level is kept, it should be added also to cost-utility, cost-benefit etc. Also, many cost-utility analyses are titled as cost-effectiveness analyses or include also other, "natural" outcomes in addition to QALYs. This needs to be kept in mind when adding information to the repository. One option could be to merge these two categories as cost-utility analyses are a special case of cost-effectiveness analyses  two very important but entirely distinct domains. Standards for HTAs differ from country to country with differing terms of reference.  In case of economic and cost evaluations the perspective of the analysis is crucial (e.g. payer perspective, societal perspective)  The difusion of the thechnology is also important. How many careproviders are using it? How many patients? In which countries is it used  I am not sure from which perspective is this to be judged upon as design tastes may differ, also culturally. Is that only within the setting used or across multiple settings?  Consider to include UX (User Experience) dimensions  What's the link between Development standards and Conflict of interest? A conflict of interest might appear in the evaluation process. As a deleloper working for a private company, I don't recognize my activity in this type of description  Missing research on safety  The interactive graph is lacking the "legal" aspect, it just has "ethical", which is at best weakly connected  Why not capture relevant PubMed IDs of articles and clinical trial numbers linked to the technology directly |
| Implementation indicators | Participants highlighted the importance of implementation attributes, as well as of post-marketing surveillance and continuous evaluation of technologies. Some additional attributes were proposed  This 'hangs' somehow as only at end 'implementation' is positioned. Perhaps try to have an earlier focus on implementation in the document  Implementation also should include a post-implementation (post-marketing) surveillance to identify and report harm from the technology  it is imperative that such an intervention would form part of the Dr/patient relationship There is a need for continuous evaluation of the product  I miss a time factor. Nurse to Patient Ratio determines the time a nurse has for the Patient. She can be well educated, however if she has to work on the shift and has no time for motivational interviewing  Consider to include other dimensions as readiness and trust  I suggest to add 'reach': The degree to which a population that is elible to benefit from an intervention actually received it (focusing on individuals)  Affordability is also important |
| 1. **Needs and expectations** | |
|  | Several comments pointed to needs and expectations of participants regarding the terminology and repository scope, aims, functionality, and potential.  Some expected more detailed descriptions to provide comprehensive information for different potential users, while others preferred more succinct descriptions feasible for MATech providers to enter in the platform.  Some participants raised questions about how data on these attributes would be gathered, updated, verified. The value of continuous MATech evaluation was highlighted. Several comments expressed expectations for continued testing and adaptation of the terminology to adapt to evolving technologies.  In the specified context, a list of barriers and enablers of medication adherence need to be linked, at personal level as well as healthcare delivery level. For example, in case of polypharmacy, the complexity may me a barrier. Existence of adverse events may be a barrier. When a multimorbid case is considered, conflicting information or lack of coordination, may be a barrier. Should we not express these relations ?  PROM, PREMS and PROMP are tools very important in order to evaluate the impact on daily activity and in QoL. Patient perspective can be an excellent point of view in which the scientific approach can be integrated  no point in measuring theoretical factors if this is to be a practical tool: surely this will be part of the certification process?  Not clear to me who evaluates these criteria and how you conclude that the categories are fulfilled  it is imperative that such an intervention would form part of the Dr/patient relationship There is a need for continuous evaluation of the product  User-centered design process.... we should change the paradigm of research: patient as partner and not as end-users  we need to include the ability to evolve, add new concepts, support evolution in state of art, incorporation of new knowledge, etc. we need to add here the ability to incorporate fragmented evidence from diverse sources, because new trials and tests on application of technology for medication adherence are not really homogeneous |
